# Cannabis- and Substance-Related Epidemiological Patterns of Chromosomal Congenital Anomalies in Europe: Geospatiotemporal and Causal Inferential Study

**DOI:** 10.3390/ijerph191811208

**Published:** 2022-09-06

**Authors:** Albert Stuart Reece, Gary Kenneth Hulse

**Affiliations:** 1 Division of Psychiatry, University of Western Australia, Crawley, WA 6009, Australia; 2 School of Medical and Health Sciences, Edith Cowan University, Joondalup, WA 6027, Australia

**Keywords:** cannabis, cannabinoid, cancer, cancerogenesis, mutagenesis, genotoxicity, epigenotoxicity, transgenerational inheritance

## Abstract

Introduction: Laboratory data link cannabinoid exposure to chromosomal mis-segregation errors. Recent epidemiological reports confirm this link and raise concern that elevated chromosomal congenital anomaly rates (CCAR) may be occurring in Europe which is experiencing increased cannabis use, daily intensity of use and cannabinoid potency. Methods: CCAR data from Eurocat. Drug use data from the European Monitoring Centre for Drugs and Drug Addiction. Income from World Bank. Bivariate, multivariate, panel and geotemporospatial regressions analyzed. Inverse probability weighting of panel models and E-values used as major quantitative causal inferential methodologies. Results: In countries where daily cannabis use was rising the trend for CCA’s was upwards whereas in those where daily use was declining it was usually downwards (*p* = 0.0002). In inverse probability weighted panel models terms for cannabis metrics were significant for chromosomal disorders, trisomies 21 and 13 and Klinefelters syndrome from *p* < 2.2 × 10^−16^. In spatiotemporal models cannabis terms were positive and significant for chromosomal disorders, genetic disorders, trisomies 21, 18 and 13, Turners and Klinefelters syndromes from 4.28 × 10^−6^, 5.79 × 10^−12^, 1.26 × 10^−11^, 1.12 × 10^−7^, 7.52 × 10^−9^, 7.19 × 10^−7^ and 7.27 × 10^−7^. 83.7% of E-value estimates and 74.4% of minimum E-values (mEV) > 9 including four values each at infinity. Considering E-values: the sensitivity of the individual disorders was trisomy 13 > trisomy 21 > Klinefelters > chromosomal disorders > Turners > genetic syndromes > trisomy 18 with mEV’s 1.91 × 10^25^ to 59.31; and daily cannabis use was the most powerful covariate (median mEV = 1.91 × 10^25^). Conclusions: Data indicate that, consistent with reports from Hawaii, Canada, Colorado, Australia and USA, CCARs are causally and spatiotemporally related to metrics and intensity of cannabis exposure, directly impact 645 MB (21.5%) of the human genome and may implicate epigenomic-centrosomal mechanisms.

## 1. Introduction

Chromosomal congenital anomalies (CCA’s) have been an important feature of several recent epidemiological studies of the associations and causal links of various prenatal drug exposures especially cannabinoids. In reports from Hawaii, Colorado, Canada, Australia and USA congenital chromosomal anomalies have featured prominently on long lists of congenital anomalies elevated after prenatal or community cannabinoid exposure [1,2,3,4,5,6,7,8,9]. Given that most of Europe has recently experienced a triply convergent rise in the prevalence of cannabinoid use, the intensity of daily use and the Δ9-tetrahydrocannabinol (THC) content of available cannabis products [10,11], it was of interest to learn if trends observed elsewhere might be reflected in European data. Since all three prongs of this triple convergence tend to increase cannabinoid exposure it is likely that cannabinoid exposure has increased on the ground more than is expressed by any one of these common metrics [3,10]. Indeed recent studies have confirmed that compound metrics of cannabinoid exposure reflect patterns of genotoxic and congenital anomaly disorders better than single more traditionally employed measures.

Particularly concerning in this regard is the well documented exponential dose response of cannabis genotoxicity [12,13,14,15,16,17,18]. It might be reasonably expected that a marked jump in community cannabinoid exposure could be expressed as a switch like mechanism in epidemiological patterns of disease as indeed appears to have occurred recently in north-eastern France where both calves and human babies are suddenly being born without limbs at greatly elevated rates 60-times those of background [19,20,21]. There are indications that in these areas large crops of cannabis are being cultivated and food chain contamination seems likely. Since epidemiological studies have confirmed that the exponentiation of cannabinoid genotoxicity seen in the laboratory is also reflected in patterns of congenital anomaly incidence [1,3,4,8,22,23,24,25] a relatively abrupt rise in community cannabinoid exposure would be expected to be associated with a relatively sudden and abrupt step-wise rise in congenital anomaly rates. This issue seems to not be well understood in the public health community.

Many mechanisms of genotoxicity from cannabinoids are described including altered sperm morphology [26,27], disruption of oocyte division [28,29,30,31], chromosomal breaks [32,33], chromosomal fusions [26,29,30,31], chromosomal bridges [29,30,31], disruption of histone synthesis [34,35,36,37,38] and post-translational modification [35], impaired histone-protamine exchange in sperm [39], oxidation of DNA bases [40], single- and double- stranded DNA breaks [40], and major changes to the DNA methylome and histone post-translational modifications which are heritable to subsequent generations [41,42,43,44,45,46,47,48,49]. Changes in the genital tracts in both sexes [50,51] and alteration of the male and female endocrine milieu is also reported [52,53,54,55,56,57,58,59,60,61] which is known to rapidly modulate the epigenome generally [62].

Considerable strides have been made in the epigenomic understanding of drug addition and drug dependency in recent years with several multigenerational animal studies appearing [41,42,44,45,46] and several studies of alterations of the DNA methylation patterns of rat and human sperm [47,48]. Particularly fascinating in this regard was the recent elegant demonstration that altered patterns of chromatin looping and ‘gene melting’ define uniquely active gene cassettes and topologically defined transcriptional domains which may account for many of the features of addiction within the dopaminergic single cell nucleus of the ventral tegmental area [63].

The subject of this study is the seven chromosomal disorders tracked by the European Network of Population-Based Registries for the Epidemiological Surveillance of Congenital Anomalies (Eurocat) namely trisomies 13, 18, 21 (syndromes of Down, Edwards and Patau) along with Klinefelters syndrome (male XXY), genetic disorders including deletions and chromosomal disorders as a group [64]. One of the major advantages of the European data is that it is complete and records all cases including stillbirths and therapeutic terminations for anomaly. The Eurocat database also reports on more congenital anomalies (95) than the USA CDC (42) [65] which means that we are able to analyze the subject of chromosomal anomalies with greater completeness than from the USA data where only five anomalies are tracked.

One of the most intriguing things about the pattern of CCA disease to be discussed is the cellular mechanisms which might be responsible. Whilst various disruptions of normal chromosomal physiology were described several decades ago as noted above [16,27,66,67], major advances in our understanding of the machinery of mitosis and meiosis have occurred in the decades since that time [68,69,70,71,72,73] and it would seem obvious from the pairing of trisomic chromosomal disorders with the monosomy of Turners syndrome [2,8] that chromosomal mis-segregation must be involved by some mechanism. However, as explored in some detail in the Discussion section, the exact mechanism of this is not understood in molecular terms. Perhaps the most likely explanations relate to disruptions of the post-translational modification code on either the tubulin which comprise the microtubules along which the chromosomes are pulled by cellular kinesin motors [74,75,76], the 90 proteins of the mammalian kinetochore which bind the chromosomes to the microtubules [77], or to the centrosomal chromatin itself [77]. Chromosomal scission and one or two whole genome doubling events [78] are also described as part of cannabinoid genotoxicity [79] all of which suggest gross disruption of the meiotic I machinery of spermatid division. These fascinating mechanistic issues are explored in more detail below.

The objective of the present study was to examine the epidemiology of congenital anomalies across Europe with regard to population substance exposure. Use of inverse probability weighting transformed the analysis from a merely observational study to a pseudo-randomized study from which it is proper and appropriate to draw causal inferences. Space-time regression was also employed to examine these changes accounting for effects such as serial correlation, spatial correlation, spatial autocorrelation and random effects. The study builds on an earlier study [3] and explores the aspects of CCA’s in more detail than was possible previously.

## 2. Methods

### 2.1. Data

Data on all available congenital anomaly rates was downloaded by each individual year for each of 14 nations from the European Network of Population-Based Registries for the Epidemiological Surveillance of Congenital Anomalies (EUROCAT) website [64] and analyzed. EUROCAT total congenital anomaly rate includes anomaly rates amongst live births, stillbirths and cases where early termination for anomaly was practised all combined together so that it represents a total overall picture across all classes of births. The nations selected were chosen on the basis of the availability of their congenital anomaly data for most of the years 2010–2019. National tobacco (percent daily tobacco use prevalence) and alcohol (litres of pure alcohol consumed per capita annually) use data were downloaded from the World Health Organization [80]. Drug use data for cannabis, amphetamines and cocaine was taken from the European Monitoring Centre for Drugs and Drug Addiction (EMCDDA) [81]. Last month cannabis use data was also supplemented by data on the tetrahydrocannabinol (THC) content of cannabis herb and resin provided in recent published reports [11]. Data on daily cannabis use was also available from EMCDDA and was collated in recent reports [11]. Median household income data (in $USD) was taken from the World Bank [82].

### 2.2. National Assignment

Nations were categorized as being either high and rising daily cannabis use or low and/or falling daily cannabis use based on a recent European epidemiological study (see Supplementary Figure S4 in [11]). Thus Belgium, Croatia, France, Germany, Italy, The Netherlands, Norway, Portugal and Spain were categorized as nations experiencing increasing daily use, while Bulgaria, Finland, Hungary, Poland and Sweden were nations which were experiencing low or falling levels of daily cannabis use.

### 2.3. Derived Data

The availability of several metrics of cannabis use, exposure and consumption made it possible to calculate various derived metrics. Hence, last month cannabis use prevalence data was multiplied by the THC content of cannabis herb and resin to derive compound metrics. These metrics were also multiplied by imputed daily cannabis use prevalence rates to derive further compound metrics for both cannabis herb and resin.

### 2.4. Data Imputation

Missing data was completed by linear interpolation. This was particularly the case for daily cannabis use. 59 data points on daily cannabis use from EMCDDA were available for these 14 nations across this period. Linear interpolation expanded this dataset to 129 datapoints (further details provided in Results section). Data on cannabis resin THC concentration were not available for Sweden. However, it was noted that the resin to herb THC concentration was almost constant in nearby Norway at 17.7 so this ratio was applied to the Swedish cannabis herb THC concentration data to derive estimates of Swedish cannabis resin THC concentration. Similarly data for the cannabis resin THC concentration in Poland were not available. The resin to herb THC concentration ratio of nearby Germany was used to estimate the resin THC content in Poland from the known Polish herb THC concentrations. Since geospatial analytical techniques do not tolerate missing data the dataset was completed by the last observation carried forward or backwards for Croatia in 2018 and 2019 and The Netherlands in 2010. It was not appropriate to use multiple imputation methods for this dataset as multiple imputation cannot be applied in panel or spatial multivariable regression techniques.

### 2.5. Statistics

Data was processed in R Studio version 1.4.1717 based on R version 4.1.1 from the Comprehensive R Archive Network and the R Foundation for Statistical Computing [83]. The analysis was conducted in December 2021. Data was manipulated using dplyr from the tidyverse [84]. Data were log transformed where appropriate to improve compliance with normality assumptions based on the results of the Shapiro–Wilks test. Graphs were drawn in ggplot2 from tidyverse. Maps were drawn using ggplot2, sf (simple features) [85] and both custom colour palettes and palettes taken from the viridis and viridisLite packages [86].

Bivariate maps were drawn with package colorplaner [87]. All illustrations are original and have not been published previously. Linear regression was conducted in Base R. Mixed effects regression was performed using package nlme [88]. In all multivariable models model reduction was by the classical technique of serial deletion of the least significant term to yield a final reduced model which is the model presented. Multiple linear models were processed in a single pass using combined techniques from R packages purrr and broom [84,89,90]. The overall effect of covariates in multivariable models may be quantified as the marginal effect. In this case, the overall marginal effect was calculated using the R package margins [91].

### 2.6. Covariate Selection

The presence of multiple different metrics for cannabis consumption and exposure created a problem for analysis as it was not clear which was the most appropriate metric to employ for any particular model. Indiscriminate use of excessive covariates in a multivariable model would unnecessarily consume degrees of freedom and thereby restrict ability to assess interactions. This issue was formally addressed by the use of random forest regression using the R package ranger [92] with variable importance being formally assessed via the R package vip (variable importance plot) [93]. The most predictive covariates from this process were entered into the regression modelling equations. The tables from this analysis are presented in the Results section.

### 2.7. Panel and Geospatial Analysis

Panel analysis was conducted using R package plm [94] across both space and time simultaneously using the “twoways” effect. The spatial weights matrix was calculated using the edge and corner “queen” relationships using R package spdep (spatial dependency) [95]. Geospatial modelling was conducted using the spatial panel random effects maximum likelihood (spreml) function from the package spml which allows detailed modelling and correction of model error structures [96,97]. Such models may produce four model coefficients of interest which are useful in determining the most appropriate error structure for the model. These coefficients are phi the random error effect, psi the serial correlation effect, rho the spatial coefficient and theta the spatial autocorrelation coefficient. In each case the most appropriate error structure was chosen for each spatial model generally taking care to preserve the model error specification across related models. The appropriate error structure was determined by the backwards methods from the full general model to the most specific model as has been described [98]. Both panel and geospatial models were temporally lagged as indicated by one to two years.

### 2.8. Causal Inference

The formal tools of causal inference were used in this analysis. Inverse probability weighting (ipw) is the technique of choice to convert a purely observational study into a pseudo-randomized study from which it is appropriate to make causal inference [99]. All multivariable panel models presented herein were inverse probability weighted. Inverse probability weighting was performed using the R package ipw. Similarly E-values (expected values) quantify the correlation required of some hypothetical unmeasured confounder covariate with both the exposure of concern and the outcome of interest in order to explain away some apparently causal relationship [100,101,102]. It therefore provides a quantitative measure of the robustness of the model to extraneous covariates which have not been accounted for within the measured parameters. E-Values have a confidence interval associated with them and the 95% lower bound of this confidence interval is reported herein. E-Value estimates greater than 1.25 are said to indicate causality [103] with E-values greater than nine being described as high [104]. E-values were calculated from the R package EValue [105]. Both inverse probability weighting and E-values are foundational and pivotal techniques used in formal causal inferential methods in order to allow causal relationships to be assessed from real world observational studies.

### 2.9. Data Availability

Raw datasets including 3800 lines of computation code in R has been made freely available through the Mendeley data repository at the following URL’s: 10.17632/mvwvxk756z.1 and 10.17632/vd6mt5r5jm.1 (accessed on 22 March 2022).

### 2.10. Ethics

Ethical approval for this study was provided from the Human Research Ethics Committee of the University of Western Australia number RA/4/20/4724 on 24 September 2021.

## 3. Results

### 3.1. Data

As shown in Supplementary Table S1 data points on total rates of 854 chromosomal congenital anomalies (inclusive of stillbirths and therapeutic abortions) were downloaded from the Eurocat database. Data was from fourteen nations as listed. Data on chromosomal anomalies, Down syndrome (Trisomy 21), Edwards syndrome (trisomy 18), Patau syndrome (trisomy 13), genetic syndromes including microdeletions, and Turner and Klinefelters syndrome were obtained with 122 data points in each group. Details on drug use, cannabis exposure, compound metrics of cannabis exposure and median household income are also shown.

As shown in Supplementary Table S2 data on daily cannabis use obtained from EMCDDA was largely incomplete with only 59 points being available. These data were completed by linear interpolation with the addition of 70 datapoints as described in the Methods section so that a total of 129 data points were available for analysis (Supplementary Table S3).

### 3.2. Bivariate Analysis

**Figure 1:**
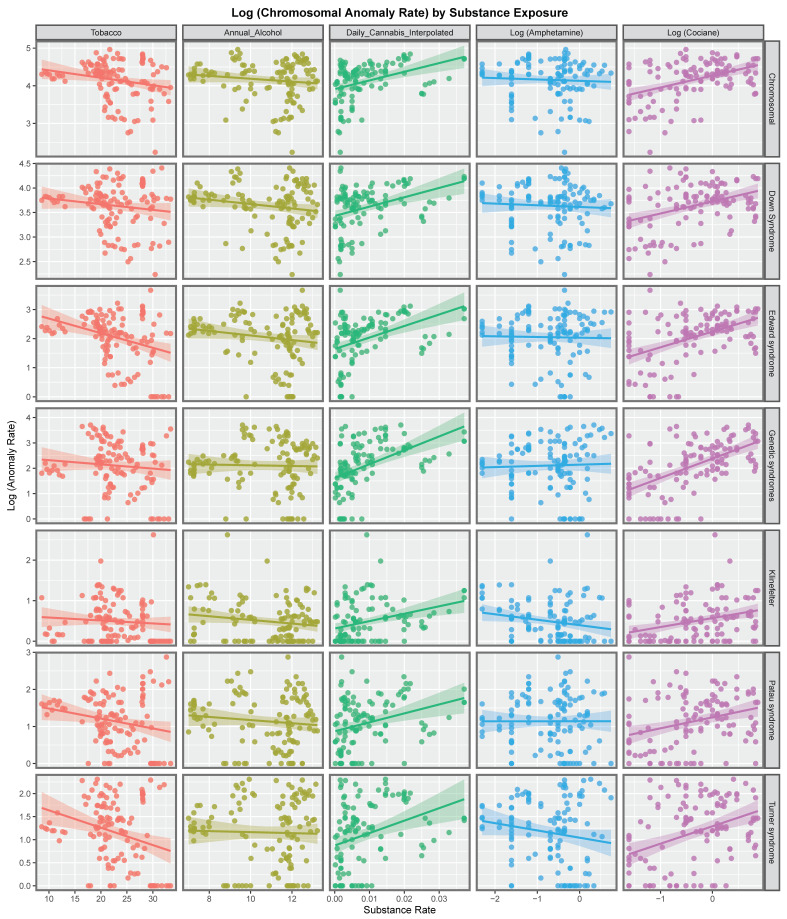
Panelled scatterplots of log (chromosomal and genetic disorder rates) by substance exposure rates.

Figure 1 shows the rate of the various chromosomal anomalies as a function of substance exposure. Interestingly the regression lines of best fit for tobacco, alcohol and amphetamine are either negatively sloping or flat. In contrast the regression lines for daily cannabis use and cocaine exposure are all positively sloping, sometimes quite steeply.

**Figure 2:**
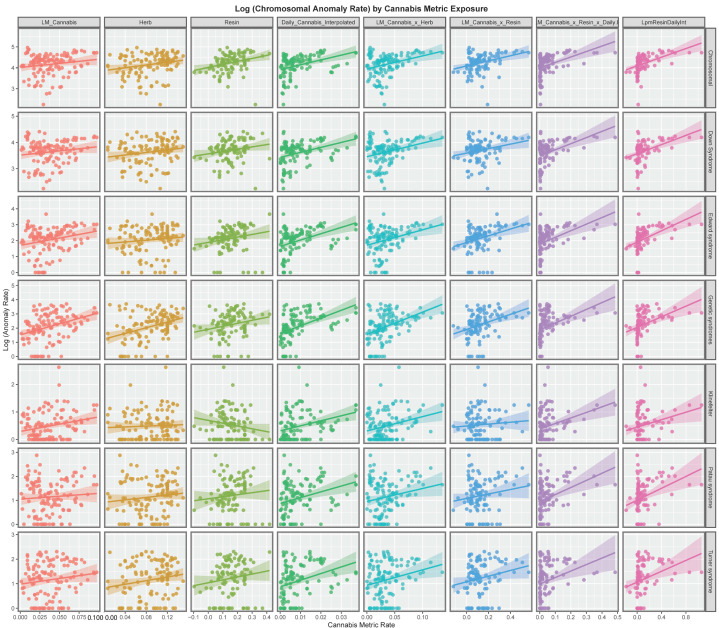
Panelled scatterplots of log (chromosomal and genetic disorder rates) by various cannabis metrics.

Figure 2 explores these relationships for each of the various metrics of cannabis exposure. The regression lines for daily cannabis use interpolated are consistently positive as are the lines for the metrics in the four columns to the right of this on the chart.

**Figure 3:**
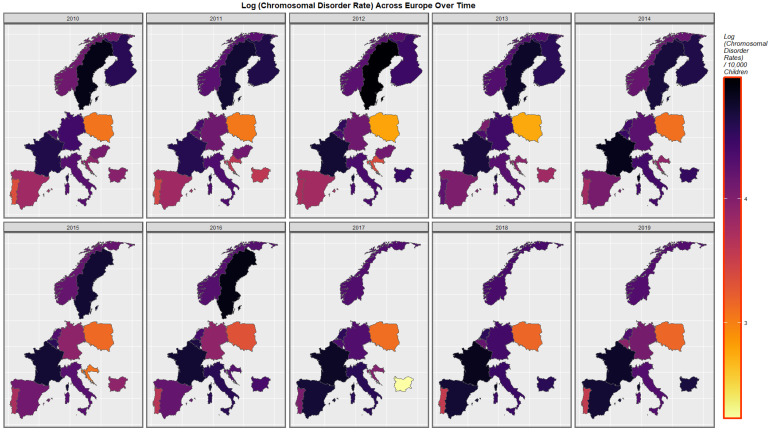
Sequential map-graph of chromosomal disorders over time in selected European nations 2010–2019.

Figure 3 presents the time course of the changes in chromosomal disorders across the European continent over this time period map graphically. High rates are observed in France, Spain, Bulgaria, Belgium and The Netherlands at different times and in Sweden until data ceased.

**Figure 4:**
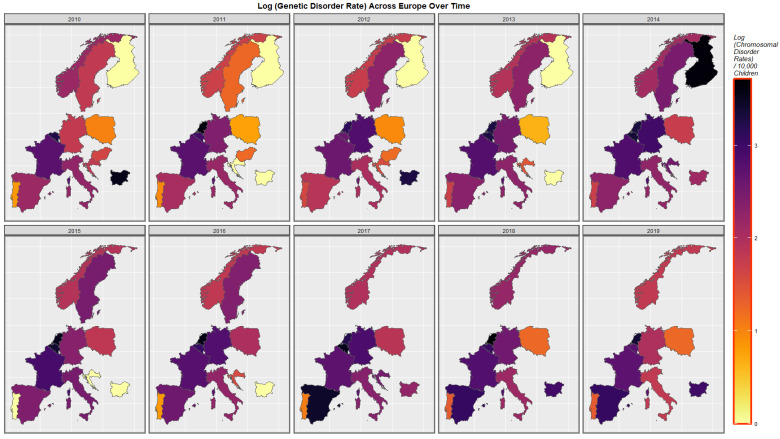
Sequential map-graph of genetic disorders over time in selected European nations 2010–2019.

Figure 4 represents the time course of genetic disorders and microdeletions. High rates are noted in The Netherlands, Belgium, Spain and Bulgaria at different periods.

**Figure 5:**
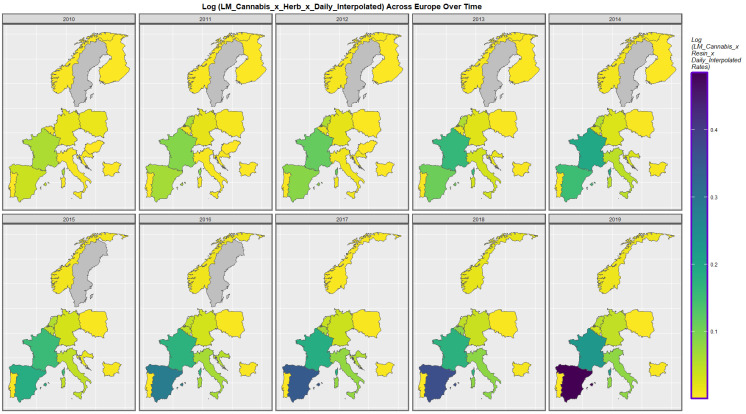
Sequential map-graph of last month cannabis use: cannabis herb THC concentration: daily cannabis use interpolated over time in selected European nations 2010–2019.

Figure 5 shows the time course of the compound cannabis exposure metric last month cannabis use x cannabis herb THC concentration x daily use interpolated. Highest rates are noted in Spain and France.

**Figure 6:**
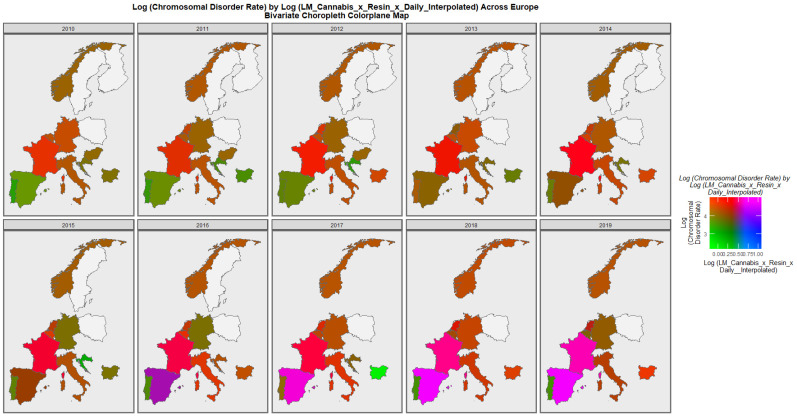
Bivariate choropleth sequential map-graph of log (chromosomal disorder rates) by last month cannabis use: cannabis resin THC concentration: daily cannabis use interpolated over time in selected European nations 2010–2019.

Figure 6 is a bivariate map of the chromosomal disorder rate as a function of the cannabis use x cannabis herb THC concentration x daily use interpolated compound metric. One notes that Spain turns from green to purple shading indicating that a nation that was once low in both the compound cannabis exposure metric and chromosomal disorders is now high in both metrics. Similarly the area of France has changed colour from red to bright pink again indicating that both the cannabis exposure metric and chromosomal disorders have now become elevated.

**Figure 7:**
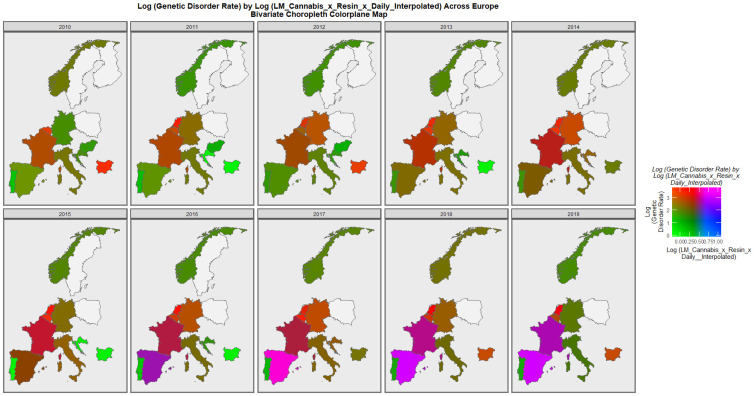
Bivariate choropleth sequential map-graph of log (genetic disorder rates) by last month cannabis use: cannabis resin THC concentration: daily cannabis use interpolated over time in selected European nations 2010–2019.

Figure 7 is a bivariate mapped plot of genetic disorders against the same compound cannabis exposure metric. Here, again a similar pattern is observed with Spain and France moving to a pattern where both exposure and outcome incidences are simultaneously high.

**Figure 8:**
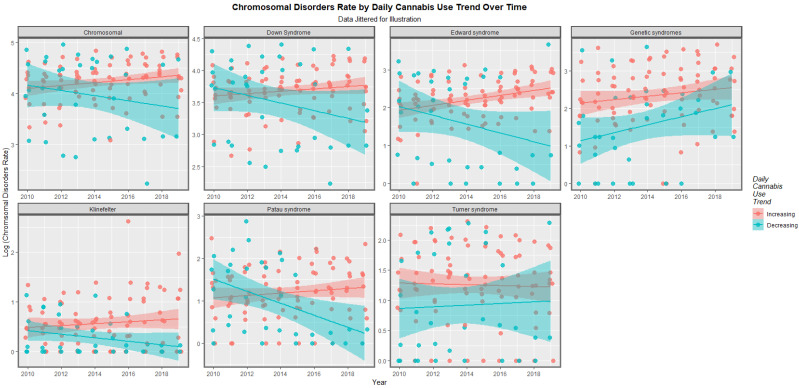
Log rates of chromosomal disorders over time by category of daily cannabis use by anomaly (see Methods section for details of assignment).

**Figure 9:**
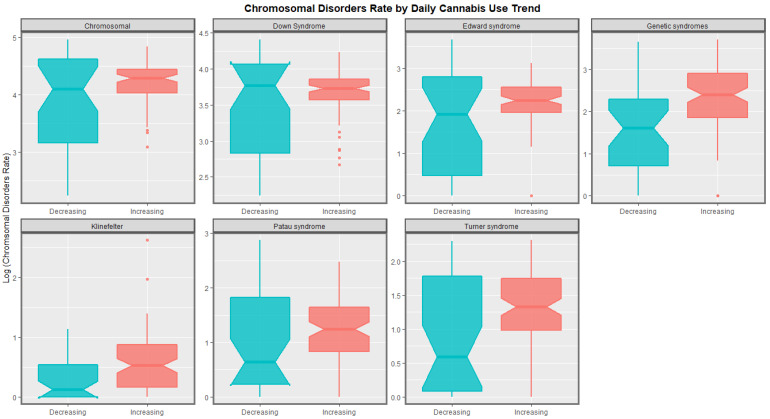
Aggregated log rates of chromosomal disorders over time by category of daily cannabis use by anomaly. Non-overlapping notches on the boxes indicate statistically significant differences.

The time trends for these seven chromosomal anomalies can also be considered dividing the nations into those where daily cannabis use is low or decreasing and those where it is rising based on recent published reports ([11] Figure 8). In most cases there was a clear temporal separation between the two groups. Indeed this is confirmed on formal testing. A mixed effects regression procedure with anomaly as the random effect found that indeed the congenital anomaly rates (CARs) were significantly higher in the group of nations where cannabis use was rising (β-est. = 0.688, t = 3.709, *p* = 2.29 × 10^−4^; model AIC = 1811.6, LogLik. = −899.798). Figure 9 charts this same data over aggregated time. The box plots are read by noting where the notches do not overlap which indicates a statistically significant difference (linear regression β-est. = 0.375, t = 3.48, *p* = 0.0005; model F = 12.1, df = 1, 852, *p* = 0.0005; E-value estimate = 1.86, minimum E-value = 1.46).

The slopes of the regression lines show in Figure 1 and Figure 2 were formally studied by linear regression in a single pass analysis using a combined purr-broom workflow for each anomaly and for each substance exposure. The results are presented in Supplementary Table S4.

When those slopes which were positive and statistically significant were extracted 51 bivariate relationships remained and these are shown in Table 1. The models are listed in descending order of minimum E-Value (mEV). It is noted that some of the E-value estimates and the mEV’s are very high. The first seven lines of the table are all occupied by daily cannabis use interpolated as the most closely associated cannabis exposure metric. Indeed the first seven lines of table list consecutively all seven of the chromosomal anomalies of interest. Indeed 24 of the 51 mEV’s reported (47.6%) are above nine and so are in the high range. All 51 mEV’s reported exceed 1.25 which is the generally accepted cut-off point for potentially causal relationships [103]. The only other covariate to appear in table is cocaine which fits with the data presented in Figure 1. Clearly these results demonstrate a very close bivariate relationship between cannabis metric exposure and the various different chromosomal pathologies.

### 3.3. Multivariable Analysis

#### 3.3.1. Random Forest Regression

The next question therefore is how would these various covariates behave in a multivariable analytical context. However, this question is not straightforward to answer due to the multiplicity of covariates listed. This issue was addressed with the use of random Forrest regression using the R package ranger together with the package vip to assess variable importance. These variable importance tables are shown as Supplementary Tables S5–S11 for the seven CAs respectively.

#### 3.3.2. Inverse Probability Weighted Panel Regression

Supplementary Table S12 presents final multivariable panel regression models (after model reduction) for chromosomal anomalies as a class. All panel models were inverse probability weighted allowing causality to be directly inferred. Selection of covariates was undertaken by the Random Forrest analysis described above. Additive and interactive models are presented together with interactive models lagged by one and two years. Confirming the bivariate results in each case daily cannabis use interpolated has invariably positive coefficients and is highly statistically significant (from *p* = 3.61 × 10^−5^ to *p* < 2.2 × 10^−16^) along with other cannabis exposure metrics.

Supplementary Tables S13–S18 repeat these procedure for the other six chromosomal anomalies. In each case metrics of cannabis exposure are positive and highly statistically significant. Since these models are all inverse probability weighted and convert the analysis from an observational study to a pseudorandomized framework they are reporting not just associations but causal inferences.

#### 3.3.3. Geospatiotemporal Regression

Data was also considered in a formal space-time framework that formally accounts for random effects, serial correlation, spatial correlation and spatial autocorrelation. Supplementary Figure S1 illustrates the derivation, editing and finalization of the spatial links between the various nations which were used to form the basis of the spatial weighting matrix used for formal space-time regression.

Table 2, Table 3, Table 4, Table 5 and Table 6, Tables S19 and S20 present the results of geospatial regression in additive, interactive and temporally lagged models. In each case one and sometimes several cannabis metrics are associated with positive regression coefficients are highly statistically significant.

The case of Klinefelter syndrome shown in Supplementary Table S20 is somewhat different. Although cannabis metrics are positive in the additive model they do not appear to be so in the interactive and lagged models. However, this dataset is deficient in that 30 of the results are artificially set to zero and are not true readings. As it has not been possible to correct this data by mathematical means the results are presented as they stand however they should only be interpreted as a result which is the least powerful of all the available scenarios (Supplementary Table S20).

### 3.4. Quantitative Causal Inference–E-Values

E-Values may be extracted from these panel (Supplementary Table S21) and geospatial models (Supplementary Table S22). When they are compiled into two lists (one for each of the E-value estimate and its mEV) it is noted that 72/86 (83.7%) E-value estimates exceed 9 an are therefore high, and all 86 (100%) exceed 1.25. When the mEV’s are considered it is noted that 64/86 (74.4%) are greater than 9 and all 86 (100%) are also above 1.25 (Table 7). Hence, this Table reports some of the most important findings in this study.

Supplementary Table S23 lists these E-values by anomaly. These results are considered collectively in using various summary statistics for the E-value estimates and the mEV’s in Table 8. In this table the various anomalies are listed in descending order of median minimum E-value. Table is notable for the remarkable size of many of these E-values.

Supplementary Table S24 lists these E-values again in order of the substance exposure described. In this table the substances are broadly grouped into the primary covariate as either herb or resin THC or daily cannabis consumption. The findings from this grouped analysis are collated into Supplementary Table S25 which presents summary statistics for these grouped exposures and again lists the cannabis exposures in descending order of median mEV. Once again daily cannabis consumption is conspicuously the most tightly related covariate.

These groups may be compared formally using Wilcoxson non-parametric tests. The results of these tests are shown in Supplementary Table S26. The E-value estimates are shown to be significantly different across all three primary exposure groups with the order of effect daily use interpolated > herb THC > resin THC as indicated in Supplementary Table S25. For the mEV’s the daily cannabis use interpolated exposure data is significantly more strongly associated than either the herb or resin groups (Supplementary Table S26).

## 4. Discussion

### 4.1. Main Results

All seven chromosomal disorders studied, both as a class and individually, are closely related to various metrics of cannabis exposure. This strong statistical signal assessed by various regression models is in close accord with recently reported results from other jurisdictions including Hawaii, Colorado, Canada, Australia and USA [1,2,3,4,5,6,7,8,9], and an earlier simpler study of the European dataset [3].

At bivariate analysis strongly positive trends for cannabis metrics, especially daily cannabis, and cannabis herb were noted. Contrariwise those for tobacco and alcohol are relatively weak (Figure 1 and Figure 2). Countries with rising levels of daily cannabis use had higher and rising levels of most chromosomal anomalies whereas the temporal trajectory of the trend in nations which did not was downwards for most anomalies (*p* = 0.0002) (Figure 8 and Figure 9).

Terms for cannabis metrics were positive and significant in inverse probability weighted panel models from *p* < 2.2 × 10^−16^ for chromosomal disorders at 1 and 2 temporal lags, Down syndrome in additive model, Patau syndrome in additive model, and Klinefelters syndrome at 2 lags (Supplementary Tables S12–S18).

Spatial regression terms including daily cannabis use interpolated were significant for chromosomal disorders from 6.13 × 10^−6^ in additive models, and in models lagged by two years 4.28 × 10^−6^ (Table 2). In an additive spatial model for genetic disorders daily cannabis use was from 5.79 × 10^−12^ (Table 3). Down syndrome assessed in an additive model was significant for cannabis herb THC concentration from 1.26 × 10^−11^ (Table 4). For trisomy 18 daily cannabis use was significant from 1.12 × 10^−7^ (Table 5). For trisomy 13 terms including cannabis herb THC concentration were significant from 7.52 × 10^−9^ (Table 6). Turner’s interactive model terms including daily use significant from 7.19 × 10^−7^ (Supplementary Table S19). Klinefelters syndrome terms including cannabis herb THC concentration significant in additive models from 7.27 × 10^−8^ (Supplementary Table S20).

83.7% of 86 E-value estimates exceeded 9 and were thus in the high range including four values at infinity. 74.4% of 86 minimum E-values exceeded 9 including four at infinity (Table 7). Daily cannabis use was shown to be a more powerful covariate than cannabis herb or resin THC concentration (Supplementary Tables S5–S11 and S26).

### 4.2. Choice of Anomalies

In view of the importance of accurately studying the detailed epidemiology of chromosomal disorders, which themselves chronicle direct genetic damage at the hundred megabase scale, it was important to consider all seven of the lesions by full and formal analysis.

### 4.3. Qualitative Causal Inference

In 1965 the renowned epidemiologist A.B. Hill described nine criteria which have become established as qualitative criteria for properly attributing a causal relationship to an association [106]. It is noted that the present analysis fulfils all nine of these criteria: strength of association, consistency amongst studies, specificity, temporality, coherence with known data, biological plausibility, dose–response effects, analogy with similar situations elsewhere, and experimental confirmation.

### 4.4. Quantitative Causal Inference

The methodologies adopted in this study formally address the two major analytical techniques employed by quantitative causal inference namely inverse probability weighting and E-values. Inverse probability weighting has the effect of evening out exposures across a whole groups of data thereby making the groups broadly comparable across the whole investigative domain and addressing the non-equivalence issue which is one of the primary criticisms levelled at observational studies. This procedure effectively transforms an observational dataset into a pseudorandomized analysis from which it is entirely appropriate to draw causal inferences [107].

E-values, or expected values take into account the degree of association required of some unidentified and uncontrolled confounding variable with both the exposure of concern and the outcome of interest to discount an apparently causal relationship [100,101,102,103]. It is noted that the very high magnitude even of the lower E-values in the present study (74.4% greater than 9 which is high [104]) ensures that discount of a causal relationship is excluded.

### 4.5. Mechanisms

#### 4.5.1. Chromosomal Overview

The fact that chromosomal anomalies have been positively identified in all six of the major epidemiological surveys of cannabis teratology to be conducted in recent years [1,2,3,4,5,6,7,8,9] clearly indicates that disturbances of chromosomal physiology must be one of the defining features of cannabinoid teratogenesis. This begs the intriguing mechanistic question as to the underlying cellular mechanisms which might be responsible for this broad spectrum of anomalies. Moreover, mechanistic considerations are central to the whole causal argument as outlined by Hill [106] including biological plausibility for the cannabis –congenital chromosomal anomaly association. For these reasons mechanistic considerations well merit careful consideration and detailed molecular dissection so that state-of-the-art insights and understandings can be brought to bear on this pivotal and fundamental question.

Considering the list of disorders presented by the rich European dataset two separate sets of processes appear to be operating when considered in the most general of terms. First, chromosomal mis-segregation likely attributable to disruptions of the mitotic machinery and/or the mitotic spindle (responsible for the series of chromosomal trisomic syndromes reported including Klinefelters disorder and the monosomy Turners syndrome). Second, genetic deletions and microdeletions which clearly involve DNA breaks and chromosomal breakage lesions. If one adds together the lengths of the chromosomes involved, here chromosomes 13, 18, 21 and X it is noted that this accounts for (113 + 76 + 46 + 153 = 388) megabases (12.9%) of the 3000 megabases in the human genome.

To this landscape of major chromosomal anomalies can be added two cancers with which cannabis is known to be associated namely testicular cancer and acute lymphoid leukaemia. Both of these malignancies are known to be associated with chromosomal breaks, chromosomal translocations and malignant chromosomal fusion events which give rise to oncogenes with constitutive function and conferring a growth advantage on the malignant clone/s. Chromosomes 1, 7, 8, 11, 12, 13, 18, 21, X and Y are implicated in testicular cancer [78] and together this accounts for (246 + 158 + 146 + 134 + 132 + 113 + 76 + 46 + 153 + 50 = 1254) of the 3000 megabases (41.8%) of the human DNA [108,109,110]. In the case of acute lymphoid leukaemia the chromosomes most commonly involved in B-cell ALL are 4, 9, 10, 11 and 22 which together account for (191 + 136 + 135 + 134 + 49 = 645) megabases (21.5%) of the human genome [111].

If the length of all of the chromosomes implicated in cannabis-related genotoxic disease is added together (excluding duplicates) it totals and impressive 1765 megabases (58.8%) of the human genome which by any measure is a sizeable segment to be registering direct genetic damage.

The case of the chromosomal pathologies in testicular cancer is particularly intriguing and its rapidity opens up special insights into chromosomal pathological mechanisms. Metanalysis has demonstrated that the incidence of testicular cancer is 2.6 times greater after cannabis exposure than without it [112]. Moreover, if one assigns a median age of 20 years to cannabis exposure, and the known median age of testicular cancer is 33 years, then cannabis exposure abbreviates the usual oncogenic incubation period from 33 to just 13 years which is a 2.5-fold acceleration. If one considers the compound index of incidence-rapidity it is noted that the oncogenic process following cannabis is (2.6 × 2.5 =) 6.5 times faster.

Moreover, the biogenesis of testicular cancer is known to involve genome demethylation, one or two rounds of whole genome doubling events followed by the loss of (usually) 50–70 chromosomal arms [78]. This oncogenic scenario is thus intriguing as cannabis has been previously linked with disruption of the meiotic machinery [47], and is also known to cause widespread alteration in patterns of DNA methylation. Most curious of all however is the issue of losing so many chromosomal arms in such a short period.

It was noted in the Introduction that chromosomal breaks, fusion and bridges have been identified in cells following cannabis exposure. In 1938 the breakage-fusion-bridge cycle was identified by which broken ends of chromosomes became joined to other broken ends as fusions which became disrupted with cell division thereby perpetuating the cycle [113].

Indeed cannabis has been linked with inhibition of telomerase potentially leading to uncapped and unprotected chromosomal ends, and accelerated aging syndromes [47,114].

If the breakage-fusion-bridge cycle was operating in the testicular germ cell niche this would explain both the relatively rapid appearance of testicular carcinogenesis after young adult exposure and its requirement to take an average of 13 years to occur whilst the cycle turns, builds up genetically modified clones which become selected out and expanded and eventually achieve a growth advantage and become clinically revealed as overt malignant disease.

Whilst such oncogenic mechanisms are not relevant to congenital chromosome inherited disorders they do powerfully inform the broader subject of cannabis-induced chromosomal pathology more generally and expand the potential repertoire of human chromosomal pathophysiology which is of relevance to the broader subject.

In considering the spectrum of chromosomal pathology in relation to cannabis it should also be noted that cannabis has long been known to test positively in the micronucleus assay [115]. Micronuclei have been identified as a key engine for the formation of chromosomal shattering, so-called chromothriptic events, which are a key engine of genetic damage and implicated in cancerogenesis, congenital anomalies mental retardation and pregnancy loss [116,117,118,119,120,121,122,123,124,125,126,127,128].

#### 4.5.2. Chromosomal Detachment

Given that chromosomal mis-segregation is so a major part of cannabinoid teratogenesis a major outstanding question must relate to the mechanism whereby chromosomes detach from the mitotic half spindle. Why do the chromosomes detach? This is clearly the source of mis-segregations which lead to trisomies, monosomies, micronucleus formation and chromothripsis.

Whilst the exact answer is not known there are many possibilities owing to the impressive complexity of the system of cell division. Tubulin proteins are a complex family of proteins. Microtubules are made of α- and β- tubulin dimers which polymerize into high order elongated structures which form the mitotic spindle. There are 25–30 microtubules in each ray of the spindle. Cannabis was shown in a proteomic screen to reduce tubulin synthesis [38] so this would obviously impede the function of the whole structure.

As mentioned there are many different types of tubulins. They are all subject to a complex system of post-translational modifications (PTM’s) which control subcellular function, address and reactivities. These PTM’s include methylation, acetylation, tyrosination, removal or addition of terminal glutamates including polyglutamylation, glycation or polyglycation, polyamination, phosphorylation, ubiquinylation and palmitoylation [76]. Acetylation of α-tubulin on lysine 40 controls microtubule flexibility and microtubule “aging” [76].

Microtubules of the spindle pointing to the cell equator are enriched for detyrosination and polyglutamylation. Unaligned chromosomes are carried to the metaphase plate by the kinetochore associated protein kinesin-7 motor protein CENPE (centromere protein E) in a manner which is dependent on the detyrosination status of the microtubules implying that the motor protein can read the tyrosination state of the microtubules [76]. Such steps are clearly critical steps which either direct or epigenomic impact of cannabinoids may perturb.

PTM’s have interesting effects, as those near the centrioles encourage nucleation and the formation of the poles of the mitotic spindle by polyglutamylation. Importantly phosphorylation of serine 172 on β-tubulin by CDK1 (cyclin dependent kinase 1) prevents incorporation of the tubulin dimer into the growing microtubule and reduces the pool of tubulin available for polymeric assembly. As noted below CDK1 is perturbed epigenomically by cannabinoid withdrawal [47].

Microtubule dynamics were shown to be affected epigenomically by differential DNA methylation in cannabis withdrawal on functional annotation of a recent Ingenuity Pathway Analysis longitudinal whole epigenome screen (58 genes, Supplement S5 page 300, *p* = 0.000033; and again on page 352, *p* = 0.00445) [47].

The microtubules are bound to the spindle by a complex multiprotein assembly called the kinetochore which is a giant molecular complex comprising 90 proteins all highly regulated and highly post-translationally modified [77]. It has been argued that sumoylation is a particular key and foundational PTM placed on several of the key subunits of the kinetochore which control the assembly of the subsequent PTM’s [77]. Δ9THC has been shown to impair desumoylation thereby disrupting the PTM code and thus kinetochore control [129].

Clearly this would be a very fruitful area for active investigation.

#### 4.5.3. Epigenomic Mechanisms

An important recent whole genome DNA methylation screen identified 163 differentially methylated regions (DMR’s) affecting hundreds of genes across the genome during cannabis dependence, and then 127 DMR’s which appeared after 11 weeks of cannabis withdrawal [47]. Three functional annotations from the Ingenuity Pathway Analysis identified for chromosomal genes were found during this process involving homologous pairing of chromosomes (MSH5, RAD21L1, SMC1B and SYCP3), chromosomal assembly (CDK1) and Philadelphia-chromosome-negative acute lymphoblastic leukaemia (CD3D, EPHA2, FYN, KMT2A).

MSH5, MutS homolog 5 is involved in DNA mismatch repair and facilitates DNA crossing over during meiosis [130]. Abnormalities of it are implicated in azoospermia and premature ovarian failure and thus infertility in both sexes. RAD21L1, Double Strand Break Repair Protein RAD21-like is involved in male prophase I of meiosis, sister chromatid paring, crossing over, synaptonemal complex formation and synapsis initiation [131]. When mutated it causes spermatogenic failure. SMC1B, Structural Maintenance of Chromosomes forms a key part of the meiosis-specific cohesin complex with RAD21 which hold sister chromatids together and is involved in DNA recombination events [132]. It is expressed in brain, heart and testis. Mutations cause gonadal dysgenesis and corneal dystrophy [132]. SYCP3, Synaptonemal Complex Protein 3, is expressed in human testis germ cells and functions during meiosis I to control chromosome separation, recombination events and synaptonemal complex formation. When mutated it causes infertility, testicular degeneration, spermatogenic failure and azoospermia in males and pregnancy loss in females [133].

CDK1 is the serine/threonine cyclin dependent kinase I which stands at the very threshold of cell cycle control [134]. CDK1 phosphorylates over 75 binding partners which control cell cycle entry, mitotic spindle formation and degradation, centromere assembly and many features relating to cell cycle progression [135]. It is also highly regulated by multiple interacting control systems including positive feedback loops which operate in switch like fashion to suddenly activate or shut down its functions [135]. It controls entry into key cell cycle phases such as the G1/S transition and the G2/M transition.

CD3D (T-cell surface glycoprotein CD3) is a cell surface glycoprotein on T-cells encoding the CD3 receptor [136]. EPHA2 is the EPH Receptor 2A involved in CNS development and congenital cataract [137]. FYN is a protein tyrosine kinase involved intracellular signalling cascades in T-cells and neurons, cell growth, fertilization, mitosis, development and cancer [138]. KMT2A is a lysine specific demethylase 2A involved in gene silencing and heterochromatin maintenance including at centromeric chromatin [139]. It performs demethylation of dimethylated lysine-36 on histone 3 (H3K36me2) at CpG islands. It performs a vital role in the maintenance of the histone code and thus controlling chromatin availability to the transcription machinery [139].

The epigenomewide screen by Schrott and colleagues also identified 256 pathway hits on terms including DNA mainly during cannabis withdrawal [47]. Central DNA functions including transcription (60 genes), promoter activation (106 genes), DNA replication, recombination and repair (12 genes), DNA binding (24 genes), DNA synthesis, replication, recombination and repair (20 genes) and cell signalling, DNA replication, recombination and repair, nucleic acid metabolism and small molecular biochemistry were a few of the pathway annotations identified.

Interestingly it was recently shown that neural progenitor cells derived from induced pluripotent stem cells from patients with Down syndrome had global suppression of transcription related to major alterations in the epigenomic state including chromosomal “introversion”, destruction of the nuclear lamina, abrogation of lamina-associated chromosomal domains, and a reduction in topologically active transcriptional domains which collectively closely recapitulated changes of neural stem cell senescence [140]. This global transcriptional suppression was thought to underlie the many neurodevelopmental and morphogenic anomalies with which Down syndrome is associated. Importantly these changes could be collectively improved in vitro when senolytic drugs were administered to the growing neural stem cells which also had the effect of rescuing overall transcription rates [140]. Such findings clearly underscore the close link between CCAR’s and accelerated aging pathophenotypes.

#### 4.5.4. Epigenomic Centrosomal Mechanisms

A fascinating recent study described the manner in which centromeric chromatin is a hotspot for DNA breaks and chromosomal translocations [141]. Indeed centromeric instability is known to be linked with cancer, congenital abnormalities and premature aging [141]. Centromeres are hotspots for chromosomal breakage and their inherent fragility is linked to recurrent breakage-fusion-bridge cycles in solid tumours. Indeed spontaneous centromeric chromosomal breakages apparently for simply structural reasons are not uncommon [141].

Investigators revealed that the modified centrosomal histone 3 variant CENP-A together with its chaperone HJURP and dimethylated lysine 4 of histone 4 (H3K4me2) enable a succession of events to allow the recruitment of the RAD51-BRCA1-BRCA2 complex of the homologous recombination pathway to induce formation of a DNA-RNA hybrid and high fidelity end repair. Moreover, inhibition of RAD51 led to the involvement of RAD52 and the activation of the low fidelity mutagenic microhomology end joining pathway leading to centromeric instability and chromosomal translocations [141].

In this context some of the findings of the recent epigenomic screen of Schrott and colleagues is particularly relevant [47]. It has recently been shown that Nuclear Mitotic Apparatus 1 (NUMA1) and the centrosomal proteins (CEP) CEP152 and CEP 55 are key to centrosomal function. The motor proteins kinesin and dynein-dynactin preform key role not only moving chromosomes but also on collecting the ends of microtubules together into cohesive mitotic spindle poles in a bipolar orientation [74,75,142,143,144,145,146,147]. Table 9 lists the EWAS hits identified in the Schrott datasets for some of these proteins. Interestingly there were 218 hits for the kinesins which were some of the most strongly associated of all the hits identified in cannabis dependence in Schrott’s Tables S1 and S4 [47]. It is noted that KIF14 is an alternate nomenclature for KIFC3 which was shown in human and animals models to be particularly involved in these roles [147]. Table 10 lists further EWAS hits from kinesin family members from the Schrott database [47].

Considering the CENP proteins 105 hits were identified (Table 11) including 86 for CENPN alone (examples shown in Table 12) which is the second CENP to bind in the cascade suggesting global disruption of centromeric chromatin decoration induced by cannabinoids and necessarily downstream centrosomal-kinetochore dysfunction as is indeed observed so prominently epidemiologically. The high level of probability reached for the CENPN epimutations is notable from this study from *p* = 7.73 × 10^−20^. Indeed this group of cancer linked mutations is amongst the most highly significant of all the DNA methylation hits identified. Laboratory studies of CENP -A, -C and -N have shown that interference with all three key CENP proteins leads to chromosomal mis-segregation errors, aneuploidy, accelerated aging and cancer [73]. Highly penetrant mutations in all three genes individually results in early embryonic lethality [73].

Indeed it has been demonstrated that as many as 90% of solid organ and 50% of haemopoietic cancers are aneuploid which directly implicates epigenomic centromeric/kinetochore processes in tumourigensis [69].

As shown in Table 13 there were nine hits for RAD51 which occurred in both cannabis dependence and withdrawal (Bonferroni-adjusted *p*-values from 0.0009 to 0.0262) and only one for RAD52 indicating that cannabis blocks RAD51 and homologous recombination much more severely than lower fidelity pathways such as microhomology-mediated repair which forces errors into centrosomal replication and downstream chromosomal mis-segregation events [141].

##### Epigenomic Gene Activation Post-Human Fertilization

The patients described in this report were born non-mosaically aneuploid, that is uniformly aneuploid in all body cells. This important finding localizes the genotoxic lesion to either the pre-fertilization male or female gametes or the first cell of the embryo, the bipronuclear fertilized zygote.

A fascinating paper recently appeared which provided details on gene activation from the transcriptome analysis of the earliest stages of human life from the oocyte held at the second meiotic division (MII) across fertilization and into the first three zygotic cell divisions to the eight cell stage [148]. An intriguing group of 21 DNA damage repair, replication, cell growth and cancer related pathways were identified by Ingenuity Pathway Analysis. Pathways identified included particularly kinetochore metaphase signalling pathways and cell cycle control of chromosomal replication pathways which are of particular relevance to the present enquiry into the possible aetiopathogenesis of cannabinoid-induced chromosomal aneuploidy.

6 of the 15 (40%) genes identified in the kinetochore metaphase signalling pathway were also shown to be differentially methylated in the EWAS sperm study of Schrott [47] including AURK (aurora kinase, the main kinases controlling mitosis), CCNB1 (cyclin B1), CDC20 (cell division cycle 20), CDK1 (cyclin dependent kinase 1), KIF2C (kinesin family member 2C) and PPP1CC (protein phosphatase 1 catalytic subunit gamma) (sampled in Table 14, Table 15 and Table S27). There were 107 hits in the Schrott EWAS for CDK1 (Table 15) and 14 for PPP1CC (Supplementary Table S27).

6 of 9 (66.7%) of the genes identified in the cell cycle control of chromosomal replication pathways were similarly identified in the EWAS analysis of Schrott namely CDK1, MCM6 (minichromosome maintenance complex component 6, involved in the initiation of chromosomal replication), ORC2 (origin replication complex 2, initiates one round of chromosomal duplication per cell cycle), PCNA (proliferating cell nuclear antigen, increases processivity of DNA polymerase delta and high fidelity DNA damage repair), POLA1 (DNA polymerase 1, which replicates DNA), and TOP2A (topoisomerase 2A, a major topoisomerase which cuts and unwinds DNA for replication and transcription) (Table 16).

##### Epigenomic Actions of Extrachromosomal DNA

The implication of cannabis in the formation of double minute chromosomes, chromosomal rings and micronuclei was noted above [26,128]. It was recently shown that one or more of these extrachromosomal DNA (ecDNA) rings can form transcription hubs within the cell nucleus which form an oncogenic factory and lead to the expression of various oncogenes acting in *trans* as enhancers for multi-chromosomal transcription [149]. Such elements are well described in several cancers where they form a key driver of transformation by acting as promiscuous enhancers. Indeed oncogenic selection of ecDNA’s occurs and oncogenic enhancer-ecDNA co-selection has also been documented in these ecDNA oncogenic hubs [149].

Such mechanisms may also support the implication of cannabis in various cancer types [2,22,23,24,25,79,150,151,152,153,154,155,156,157,158,159,160,161,162,163,164,165].

## 5. Exponential Genotoxicity

Numerous laboratory studies document that cannabis genotoxicity and the processes on which genetic stability rests, exhibit an exponential dose–response relationship. Assays of cell growth and cell viability, mutagenesis, inhibition of tubulin, action, DNA and RNA synthesis, and inhibition of DNA methylation resulting in morphine self-administration in offspring all demonstrate exponential dose response relationships [12,13,14,15,16,17,18].

Moreover, this clear exponentiation of dose–response genetic damage in the laboratory is reflected in similar changes epidemiologically as has been well demonstrated in USA [2,22,23,24].

This now well documented exponentiation becomes a major issue downstream of the rising triple convergence of cannabis use prevalence, daily dosing intensity and cannabinoid potency which is widely reported on both sides of the Atlantic [10,11,166,167] and opens whole communities to sudden encounters with major genotoxic outcomes such as has recently been reported from France with limbless cows and babies occurring at 60 times the background rate [19,20,21] and in Mississippi and Kentucky where rate of atrial septal defect has suddenly shot up 20 times that of ten years ago [168].

## 6. Generalizability

A number of factors provide confidence that study results are robust and widely generalizable: 1. Data is based on one of the largest most comprehensive datasets globally; 2. Findings are strongly concordant with all of the other peer reviewed published data in the field internationally [1,2,3,4,5,6,7,8,9]; 3. Both the quantitative and qualitative epidemiological criteria of causality are fulfilled; 4. There is an abundance of causal mechanisms to explain this findings; 5. Results have both internally and external consistency; 6. Similar outcomes were derived by several different regression techniques; 7. Findings have been demonstrated in their native space-time context. Indeed the signals derived are some of the strongest statistical signals seen in all of the field of cannabis teratology.

## 7. Strengths and Limitations

The strengths of the present study are that it used one of the world’s largest most comprehensive datasets, used the database with information on the most number of genetic pathologies, employed both inverse probability weighting and E-values [99,100,104,169,170], the major techniques of causal inference [102,103,104,171], employed several different forms of regression including spatiotemporal regression [96,97,98], demonstrated both internal consistency and found external concordance with other published results in this field [1,2,3,4,5,6,7,8,9]. Random Forrest regression was used for formal variable selection [92]. Study limitations include that of many epidemiological studies that it did not have availability to individual participant level data. Incomplete data was also an issue both for daily cannabis exposure and Klinefelter’s syndrome. Nevertheless, we feel that the way we have addressed the issue of missing data is reasonable and has been openly transparent.

## 8. Conclusions

Data present strong evidence demonstrating in inverse probability weighted and causal inferential models that various metrics of cannabis use, especially daily use, are strongly associated with all seven of the chromosomal disorders studied. These findings confirm both many decades of in vitro laboratory work and also several recent epidemiological series from Hawaii, Colorado, Australia, Canada and the USA, as well as an earlier simpler analysis of this same dataset. Naturally these findings are of great concern in their own right. However, when considered in terms of the known exponential dose–response relationship of cannabis genotoxicity and the increasing contamination of the food chain in Europe and parts of the USA, public health concerns must be heightened. The present findings form part of the interface of cannabinoids with the broader spectrum of congenital anomalies which is also an area of great concern. Together with cannabinoid-related carcinogenesis and cannabinoid-potentiated aging syndromes cannabis-related congenital chromosomal anomalies, findings form one pivotal plank of overall cannabinoid genotoxicity. As has been shown elsewhere and also in the present results that cannabis is a much more potent genotoxic than tobacco and alcohol combined (see Table 1, Table 2, Table 3, Table 4, Table 5, Table 6, Table 7 and Table 8 and Tables S3–S17) it is clear that a rational evidence driven policy in relation to cannabis would restrict access to cannabinoids in the same way as for other powerful genotoxic environmental compounds. As discussed above the field is wide open for genotoxic and epigenotoxic mechanistic studies to investigate epigenetic mechanisms of chromosomal mis-segregation and mitotic spindle dysfunction, cannabinoid-induced chromosomal scission and the gross disorders of meiosis I which lead to increased whole genome doubling events and are part of the testicular cancer oncobiogenesis pathway and the cellular pathology of epigenomic aging. Because CCA’s are relatively common it would also be important for future research studies to focus on higher spatial and temporal resolution space-time studies. In time, as inverse probability weighting of geospatial models becomes available such new methodologies which directly interrogate causality could also be applied to these important datasets.

## Figures and Tables

**Table 1:** Positive and Significant Slopes from Bivariate Linear Regressions.

Anomaly	Substance	Mean Anomaly Rate	Estimate	Std. Error	Sigma	t_Statistic	*p*_Value	E-Value Estimate	E-Value Lower Bound
Genetic syndromes	Daily.Interpol.	5.9209	54.6837	9.3918	0.8815	5.8225	5.53 × 10^−8^	6.55 × 10^24^	3.81 × 10^16^
Edward syndrome	Daily.Interpol.	4.9688	39.6511	8.1134	0.7616	4.8871	3.41 × 10^−6^	7.55 × 10^20^	4.39 × 10^12^
Chromosomal	Daily.Interpol.	35.8047	23.0358	5.0643	0.4754	4.5487	1.37 × 10^−5^	2.84 × 10^19^	1.65 × 10^11^
Down Syndrome	Daily.Interpol.	20.8129	19.0578	4.4071	0.4137	4.3244	3.31 × 10^−5^	3.22 × 10^18^	1.87 × 10^10^
Turner syndrome	Daily.Interpol.	1.7968	27.0000	7.2926	0.6845	3.7024	3.32 × 10^−4^	7.75 × 10^15^	4.51 × 10^7^
Klinefelter	Daily.Interpol.	0.6084	18.2674	5.0311	0.4722	3.6309	4.26 × 10^−4^	3.87 × 10^15^	2.25 × 10^7^
Patau syndrome	Daily.Interpol.	1.7793	24.1206	7.0551	0.6622	3.4189	8.75 × 10^−4^	4.96 × 10^14^	2.89 × 10^6^
Genetic syndromes	LMCannabis_Herb	5.9209	15.6419	3.1034	0.8908	5.0402	1.66 × 10^−6^	1.74 × 10^7^	3.53 × 10^4^
Genetic syndromes	LM_Cannabis	5.9209	15.5798	3.7939	0.9182	4.1065	7.37 × 10^−5^	1.02 × 10^7^	6.50 × 10^3^
Genetic syndromes	Herb	5.9209	10.0478	2.3492	0.9135	4.2771	3.82 × 10^−5^	4.45 × 10^4^	456.72
Chromosomal	LMCannabis_Herb	35.8047	6.2955	1.7442	0.5007	3.6095	4.49 × 10^−4^	1.86 × 10^5^	377.43
Edward syndrome	LMCannabis_Herb	4.9688	9.7114	2.7935	0.8019	3.4764	7.08 × 10^−4^	1.22 × 10^5^	247.40
Down Syndrome	LMCannabis_Herb	20.8129	5.2012	1.5292	0.4390	3.4013	9.12 × 10^−4^	9.63 × 10^4^	194.83
Klinefelter	LMCannabis_Herb	0.6084	5.2172	1.6420	0.4713	3.1774	0.0019	4.74 × 10^4^	95.58
Down Syndrome	LM.Cannabis_x_Herb.THC_x_Daily.Interpol.	20.8129	2.3272	0.4751	0.4056	4.8985	3.25 × 10^−6^	369.89	45.54
Chromosomal	LM.Cannabis_x_Herb.THC_x_Daily.Interpol.	35.8047	2.6195	0.5532	0.4723	4.7349	6.41 × 10^−6^	310.61	38.17
Edward syndrome	LM_Cannabis	4.9688	9.1559	3.3741	0.8166	2.7136	0.0076	5.40 × 10^4^	34.01
Edward syndrome	LM.Cannabis_x_Herb.THC_x_Daily.Interpol.	4.9688	4.1359	0.9014	0.7696	4.5883	1.16 × 10^−5^	265.61	32.57
Klinefelter	LM_Cannabis	0.6084	5.2968	1.9692	0.4766	2.6898	0.0082	4.94 × 10^4^	31.06
Genetic syndromes	LM.Cannabis_x_Herb.THC_x_Daily.Interpol.	5.9209	4.8225	1.0864	0.9275	4.4389	2.11 × 10^−5^	226.42	27.70
Patau syndrome	LM.Cannabis_x_Herb.THC_x_Daily.Interpol.	1.7793	2.9541	0.7660	0.6539	3.8567	1.92 × 10^−4^	121.51	14.65
Turner syndrome	LMCannabis_Herb	1.7968	6.2946	2.4593	0.7060	2.5595	0.0117	6.68 × 10^3^	13.03
Klinefelter	LM.Cannabis_x_Herb.THC_x_Daily.Interpol.	0.6084	2.0130	0.5530	0.4721	3.6401	4.12 × 10^−4^	96.35	11.52
Chromosomal	LM.Cannabis_x_Resin.THC_x_Daily.Interpol.	35.8047	1.0375	0.1974	0.3694	5.2563	8.60 × 10^−7^	25.26	9.43
Edward syndrome	LM.Cannabis_x_Resin.THC_x_Daily.Interpol.	4.9688	1.7864	0.3612	0.6758	4.9465	3.13 × 10^−6^	21.66	8.03
Genetic syndromes	LMCannabis_Resin	5.9209	2.6736	0.7047	0.8438	3.7940	2.48 × 10^−4^	35.24	7.55
Turner syndrome	LM.Cannabis_x_Herb.THC_x_Daily.Interpol.	1.7968	2.6440	0.8118	0.6930	3.2570	0.0015	63.88	7.47
Down Syndrome	LM.Cannabis_x_Resin.THC_x_Daily.Interpol.	20.8129	0.8706	0.1835	0.3434	4.7435	7.14 × 10^−6^	19.57	7.22
Chromosomal	LMCannabis_Resin	35.8047	1.2280	0.3399	0.4070	3.6129	4.66 × 10^−4^	30.64	6.50
Edward syndrome	LMCannabis_Resin	4.9688	2.1258	0.5980	0.7160	3.5549	5.68 × 10^−4^	29.30	6.20
Chromosomal	Resin	35.8047	1.4387	0.4591	0.4127	3.1340	0.0022	47.23	6.05
Chromosomal	Herb	35.8047	3.2385	1.3231	0.5145	2.4478	0.0158	614.55	5.77
Genetic syndromes	LM.Cannabis_x_Resin.THC_x_Daily.Interpol.	5.9209	1.8703	0.4563	0.8539	4.0989	8.57 × 10^−5^	14.16	5.12
Patau syndrome	LMCannabis_Herb	1.7793	5.4238	2.4275	0.6968	2.2343	0.0273	2.38 × 10^3^	4.27
Patau syndrome	LM.Cannabis_x_Resin.THC_x_Daily.Interpol.	1.7793	1.2368	0.3458	0.6472	3.5761	5.44 × 10^−4^	10.86	3.82
Genetic syndromes	Cocaine	5.9209	0.7636	0.1002	0.8051	7.6174	6.61 × 10^−12^	4.17	3.21
Down Syndrome	LMCannabis_Resin	20.8129	0.8596	0.3201	0.3833	2.6853	0.0084	14.88	2.88
Turner syndrome	LM.Cannabis_x_Resin.THC_x_Daily.Interpol.	1.7968	1.1097	0.3598	0.6732	3.0845	0.0026	8.43	2.86
Edward syndrome	Cocaine	4.9688	0.5400	0.0924	0.7423	5.8418	4.52 × 10^−8^	3.29	2.48
Chromosomal	Cocaine	35.8047	0.3167	0.0589	0.4732	5.3748	3.83 × 10^−7^	3.08	2.31
Down Syndrome	Cocaine	20.8129	0.2455	0.0527	0.4229	4.6615	8.20 × 10^−6^	2.78	2.06
Klinefelter	LM.Cannabis_x_Resin.THC_x_Daily.Interpol.	0.6084	0.6802	0.2641	0.4941	2.5759	0.0115	6.46	2.04
Down Syndrome	Herb	20.8129	2.4157	1.1613	0.4516	2.0801	0.0396	259.68	2.01
Turner syndrome	Cocaine	1.7968	0.3768	0.0835	0.6703	4.5142	1.49 × 10^−5^	2.72	2.01
Klinefelter	Cocaine	0.6084	0.2203	0.0577	0.4634	3.8186	2.14 × 10^−4^	2.45	1.77
Genetic syndromes	Resin	5.9209	2.1135	0.9795	0.8805	2.1578	0.0332	17.26	1.75
Patau syndrome	Cocaine	1.7793	0.2952	0.0844	0.6774	3.4991	6.56 × 10^−4^	2.34	1.67
Turner syndrome	Daily.Interpol.	1.7968	3.7174	1.8333	0.7129	2.0277	0.0448	229.61	1.65
Down Syndrome	Resin	20.8129	0.8844	0.4322	0.3886	2.0460	0.0433	15.35	1.42
Edward syndrome	Resin	4.9688	1.6669	0.8273	0.7437	2.0149	0.0465	14.86	1.32
Turner syndrome	LMCannabis_Resin	1.7968	1.1661	0.5756	0.6892	2.0259	0.0453	8.80	1.29

**Table 2:** Multivariate Geospatial Regression for Chromosomal Disorders.

Parameter Values			Model Parameters		
Parameter	Estimate (C.I.)	*p*-Value	Parameter	Value	Significance
***Additive***					
*Rate ~ Tobacco + Alcohol + LM.Cannabis_x_Herb.THC_x_Daily.Interpol. + LM.Cannabis_x_Resin.THC_x_Daily.Interpol. + Daily.Interpol. + LM.Cannabis_x_Resin.THC + Amphetamines + Cocaine + Income)*					
Alcohol	0.08 (0.05, 0.12)	7.28 × 10^−7^	rho	0.5667	2.10 × 10^−8^
LM.Cannabis_x_Resin.THC	1.47 (0.83, 2.11)	6.13 × 10^−6^	lambda	−0.471	1.95 × 10^−6^
Amphetamines	−0.21 (−0.29, −0.13)	3.01 × 10^−7^			
Cocaine	0.28 (0.19, 0.37)	1.27 × 10^−9^			
Income	0 (0, 0)	1.07 × 10^−10^			
***Interactive***					
Rate ~ Tobacco * Daily.Interpol. + LM.Cannabis_x_Resin.THC * LM.Cannabis_x_Resin.THC_x_Daily.Interpol. + LM.Cannabis_x_Herb.THC_x_Daily.Interpol. + Alcohol + Amphetamines + Cocaine + Income					
Tobacco	0.08 (0.05, 0.11)	2.11 × 10^−7^	rho	0.2807	0.0626
Daily.Interpol.	81.6 (25.54, 137.66)	0.004384	lambda	−0.2844	0.0434
LM.Cannabis_x_Resin.THC	1.61 (0.94, 2.28)	2.66 × 10^−6^			
Amphetamines	−0.28 (−0.37, −0.19)	1.11 × 10^−9^			
Cocaine	0.28 (0.1, 0.46)	0.002204			
Income	0 (0, 0)	2.61 × 10^−10^			
Tobacco: Daily.Interpol.	−3.7 (−5.88, −1.52)	0.000847			
***1 Lag***					
Rate ~ Tobacco * Daily.Interpol. + LM.Cannabis_x_Resin.THC * LM.Cannabis_x_Resin.THC_x_Daily.Interpol. + LM.Cannabis_x_Herb.THC_x_Daily.Interpol. + Alcohol + Amphetamines + Cocaine + Income					
Tobacco	0.06 (0.02, 0.09)	0.003713	rho	0.4067	0.0137
Daily.Interpol.	67.7 (3.22, 132.18)	0.039664	lambda	−0.3299	0.0193
LM.Cannabis_x_Resin.THC	1.75 (0.94, 2.56)	2.45 × 10^−5^			
Alcohol	0.05 (0, 0.1)	0.05342			
Amphetamines	−0.3 (−0.4, −0.21)	1.33 × 10^−9^			
Cocaine	0.4 (0.18, 0.61)	0.000335			
Income	0 (0, 0)	2.12 × 10^−6^			
Tobacco: Daily.Interpol.	−3.27 (−5.72, −0.82)	0.009025			
***2 Lags***					
Rate ~ Tobacco * LM.Cannabis_x_Herb.THC + LM.Cannabis_x_Resin.THC_x_Daily.Interpol. + LM.Cannabis_x_Herb.THC_x_Daily.Interpol. + Alcohol + Daily.Interpol. + Amphetamines + Cocaine + Income					
Tobacco	0.12 (0.09, 0.16)	1.26 × 10^−10^	rho	0.5497	7.96 × 10^−6^
LM.Cannabis_x_Herb.THC	40.1 (20.52, 59.68)	5.89 × 10^−5^	lambda	−0.3245	0.0144
LM.Cannabis_x_Resin.THC_x_Daily.Interpol.	3.1 (1.78, 4.42)	4.28 × 10^−6^			
LM.Cannabis_x_Herb.THC_x_Daily.Interpol.	−4 (−7.55, −0.45)	0.02668			
Amphetamines	−0.33 (−0.45, −0.21)	9.89 × 10^−8^			
Cocaine	0.26 (0.08, 0.44)	0.00442			
Income	0 (0, 0)	3.58 × 10^−13^			
Tobacco: LM.Cannabis_x_Herb.THC	−1.79 (−2.54, −1.04)	3.25 × 10^−6^			

*: signifies interactive terms.

**Table 3:** Multivariate Geospatial Regression for Genetic Disorders.

Parameter Values			Model Parameters		
Parameter	Estimate (C.I.)	*p*-Value	Parameter	Value	Significance
***Additive***					
*Rate ~ Tobacco + Alcohol + LM.Cannabis_x_Resin.THC_x_Daily.Interpol. + LM.Cannabis_x_Herb.THC + Daily.Interpol. + LM.Cannabis_x_Herb.THC_x_Daily.Interpol. + Amphetamines + Cocaine + Income*					
Alcohol	0.08 (0.01, 0.15)	0.029861	rho	0.45269	1.85 × 10^−7^
Daily.Interpol.	45.7 (32.69, 58.71)	5.79 × 10^−12^			
Income	0 (0, 0)	0.000306			
***Interactive***					
Rate ~ Tobacco * Daily.Interpol. + LM.Cannabis_x_Herb.THC + LM.Cannabis_x_Herb.THC_x_Daily.Interpol. + Alcohol + Amphetamines + Cocaine + Income					
Tobacco	0.12 (0.06, 0.17)	3.24 × 10^−5^	Least Squares		
Daily.Interpol.	299 (185.32, 412.68)	2.58 × 10^−7^	S.D.	0.6888	
Income	0 (0, 0)	5.93 × 10^−7^			
Tobacco: Daily.Interpol.	−10.6 (−15.23, −5.97)	7.79 × 10^−6^			
***1 Lag***					
Rate ~ Tobacco * LM.Cannabis_x_Herb.THC +Daily.Interpol. + LM.Cannabis_x_Resin.THC_x_Daily.Interpol. + LM.Cannabis_x_Herb.THC_x_Daily.Interpol. + Alcohol + Amphetamines + Cocaine + Income					
LM.Cannabis_x_Herb.THC	38.26 (16.43, 60.1)	0.0006	rho	0.4767	1.00 × 10^−7^
LM.Cannabis_x_Resin.THC_x_Daily.Interpol.	3.53 (1.66, 5.4)	0.0002			
LM.Cannabis_x_Herb.THC_x_Daily.Interpol.	−7.65 (−13.21, −2.1)	0.0069			
Alcohol	0.16 (0.09, 0.24)	3.27 × 10^−5^			
Cocaine	0.79 (0.54, 1.04)	4.18 × 10^−10^			
LM.Cannabis_x_Herb.THC: LM.Cannabis_x_Resin.THC_x_Daily.Interpol.	−1.34 (−2.09, −0.58)	0.000494			
***2 Lags***					
Rate ~ Tobacco * Daily.Interpol. + LM.Cannabis_x_Herb.THC + LM.Cannabis_x_Herb.THC_x_Daily.Interpol. + Alcohol + Amphetamines + Cocaine + Income					
Tobacco	0.1 (0.02, 0.18)	0.0111	psi	0.3373	0.00123
Daily.Interpol.	278 (111.01, 444.99)	0.0011	rho	0.3269	0.00149
Income	0 (0, 0)	0.0086			
Resin	−9.72 (−16.54, −2.9)	0.0052			

*: signifies interactive terms.

**Table 4:** Multivariate Geospatial Regression for Down Syndrome (Trisomy 21).

Parameter Values			Model Parameters		
Parameter	Estimate (C.I.)	*p*-Value	Parameter	Value	Significance
***Additive***					
*Rate ~ Tobacco + Alcohol + LM.Cannabis_x_Resin.THC_x_Daily.Interpol. + LM.Cannabis_x_Herb.THC_x_Daily.Interpol. + LM.Cannabis_x_Herb.THC + LM.Cannabis_x_Herb.THC + Amphetamines + Cocaine + Income*					
Tobacco	0.05 (0.03, 0.07)	1.80 × 10^−8^	rho	−0.3628	0.0628
Alcohol	−0.07 (−0.1, −0.04)	2.28 × 10^−5^	lambda	0.2088	0.261
LM.Cannabis_x_Herb.THC	6.89 (4.89, 8.89)	1.26 × 10^−11^			
Amphetamines	−0.09 (−0.17, −0.02)	0.0138			
Income	0 (0, 0)	1.02 × 10^−12^			
***Interactive***					
Rate ~ Tobacco * LM.Cannabis_x_Herb.THC + LM.Cannabis_x_Herb.THC_x_Daily.Interpol. * LM.Cannabis_x_Resin.THC_x_Daily.Interpol. + LM.Cannabis_x_Resin.THC + Alcohol + Herb + Amphetamines + Cocaine + Income					
Tobacco	0.03 (0.02, 0.05)	0.0001	rho	0.3593	0.0319
LM.Cannabis_x_Resin.THC	1.18 (0.57, 1.79)	0.0002	lambda	−0.3577	0.0334
Amphetamines	−0.2 (−0.29, −0.12)	2.24 × 10^−6^			
Cocaine	0.2 (0.11, 0.29)	2.29 × 10^−5^			
Income	0 (0, 0)	1.43 × 10^−6^			
***2 Lags***					
Rate ~ Tobacco * LM.Cannabis_x_Herb.THC_x_Daily.Interpol. + LM.Cannabis_x_Herb.THC * LM.Cannabis_x_Resin.THC_x_Daily.Interpol. + Alcohol + Amphetamines + Cocaine + Income					
Tobacco	0.05 (0.03, 0.08)	1.86 × 10^−5^	rho	−0.1896	0.435
LM.Cannabis_x_Resin.THC_x_Daily.Interpol.	1.24 (0.7, 1.78)	6.56 × 10^−6^	lambda	0.1934	0.402
Alcohol	−0.06 (−0.1, −0.02)	0.0052			
Amphetamines	−0.17 (−0.27, −0.07)	0.0008			
Income	0 (0, 0)	1.64 × 10^−7^			

*: signifies interactive terms.

**Table 5:** Multivariate Geospatial Regression for Edwards Syndrome (Trisomy 18).

Parameter Values			Model Parameters		
Parameter	Estimate (C.I.)	*p*-Value	Parameter	Value	Significance
***Additive***					
*Rate ~ Tobacco + Alcohol + LM.Cannabis_x_Resin.THC + Daily.Interpol. + LM.Cannabis_x_Herb.THC_x_Daily.Interpol. + Herb + Amphetamines + Cocaine + Income*					
Tobacco	0.05 (0.02, 0.08)	0.0012	rho	−0.5792	1.18 × 10^−5^
Alcohol	−0.13 (−0.18, −0.07)	2.96 × 10^−6^	lambda	0.5089	4.74 × 10^−5^
LM.Cannabis_x_Resin.THC	1.02 (0.29, 1.75)	0.0058			
Daily.Interpol.	33.8 (20.82, 46.78)	3.35 × 10^−7^			
Income	0 (0, 0)	1.24 × 10^−10^			
***Interactive***					
Rate ~ Tobacco * LM.Cannabis_x_Resin.THC_x_Daily.Interpol. * LM.Cannabis_x_Resin.THC + Daily.Interpol. + LM.Cannabis_x_Herb.THC_x_Daily.Interpol. + Alcohol + Amphetamines + Cocaine + Income					
Tobacco	0.04 (0.01, 0.07)	0.0112	rho	−0.5461	3.84 × 10^−5^
Alcohol	−0.07 (−0.12, −0.02)	0.0093	lambda	0.4785	8.76 × 10^−5^
Amphetamines	−0.17 (−0.29, −0.05)	0.0067			
Cocaine	0.37 (0.18, 0.55)	0.0001			
Income	0 (0, 0)	4.26 × 10^−6^			
Tobacco: LM.Cannabis_x_Resin.THC_x_Daily.Interpol.	0.09 (0.03, 0.14)	0.0020			
Tobacco: LM.Cannabis_x_Resin.THC_x_Daily.Interpol.: LM.Cannabis_x_Resin.THC	−0.12 (−0.23, 0)	0.049823			
***2 Lags***					
*Rate ~ Tobacco + Daily.Interpol. + LM.Cannabis_x_Resin.THC + LM.Cannabis_x_Resin.THC_x_Daily.Interpol. + LM.Cannabis_x_Herb.THC_x_Daily.Interpol. + Alcohol + Amphetamines + Cocaine + Income*					
Tobacco	0.04 (0.01, 0.07)	0.0185	rho	−0.6449	6.04 × 10^−8^
Daily.Interpol.	67.2 (42.31, 92.09)	1.12 × 10^−7^	lambda	0.4422	0.0014
LM.Cannabis_x_Resin.THC	3.86 (2.27, 5.45)	1.90 × 10^−6^			
LM.Cannabis_x_Herb.THC_x_Daily.Interpol.	−6.68 (−10.85, −2.51)	0.0017			
Alcohol	−0.11 (−0.17, −0.05)	0.0004			
Amphetamines	−0.17 (−0.32, −0.02)	0.0267			
Income	0 (0, 0)	4.29 × 10^−7^			

*: signifies interactive terms.

**Table 6:** Multivariate Geospatial Regression for Patau Syndrome (Trisomy 13).

Parameter Values			Model Parameters		
Parameter	Estimate (C.I.)	*p*-Value	Parameter	Value	Significance
***Additive***					
*Rate ~ Tobacco + Alcohol + Daily.Interpol. + LM.Cannabis_x_Resin.THC_x_Daily.Interpol. + LM.Cannabis_x_Herb.THC + LM.Cannabis_x_Herb.THC_x_Daily.Interpol. + Herb + Amphetamines + Cocaine + Income*					
Alcohol	0.07 (0.02, 0.13)	0.00819	rho	0.5696	4.39 × 10^−8^
LM.Cannabis_x_Herb.THC	14.6 (9.64, 19.56)	7.52 × 10^−9^	lambda	−0.6085	1.48 × 10^−9^
Herb	−5.15 (−9.44, −0.86)	0.0189			
Amphetamines	−0.14 (−0.26, −0.02)	0.0174			
Income	0 (0, 0)	2.94 × 10^−12^			
***Interactive***					
Rate ~ Tobacco * Daily.Interpol. + LM.Cannabis_x_Herb.THC * LM.Cannabis_x_Resin.THC_x_Daily.Interpol. + LM.Cannabis_x_Herb.THC_x_Daily.Interpol. + Alcohol + Amphetamines + Cocaine + Income					
Tobacco	0.04 (0.01, 0.07)	0.0226	rho	0.5546	3.44 × 10^−6^
LM.Cannabis_x_Herb.THC	9.82 (5.04, 14.6)	5.79 × 10^−5^	lambda	−0.589	4.27 × 10^−7^
Amphetamines	−0.16 (−0.28, −0.03)	0.0169			
Income	0 (0, 0)	4.81 × 10^−9^			
***2 Lags***					
Rate ~ Tobacco * LM.Cannabis_x_Herb.THC + Daily.Interpol. * LM.Cannabis_x_Resin.THC_x_Daily.Interpol. + Alcohol + Daily.Interpol. + Amphetamines + Cocaine + Income					
Tobacco	0.05 (0.01, 0.09)	0.0105	rho	0.1822	0.459
LM.Cannabis_x_Resin.THC_x_Daily.Interpol.	1.74 (0.8, 2.68)	0.0003	lambda	−0.1076	0.644
Amphetamines	−0.23 (−0.4, −0.07)	0.0060			
Income	0 (0, 0)	1.64 × 10^−7^			

*: signifies interactive terms.

**Table 7:** List of All E-Values.

No.	E-Value Estimate	Lower Bound E-Value
1	Infinity	Infinity
2	Infinity	Infinity
3	Infinity	Infinity
4	Infinity	Infinity
5	2.17 × 10^129^	2.02 × 10^71^
6	2.17 × 10^129^	2.02 × 10^71^
7	1.15 × 10^99^	2.21 × 10^54^
8	1.15 × 10^99^	2.21 × 10^54^
9	2.20 × 10^40^	2.16 × 10^34^
10	2.20 × 10^40^	2.16 × 10^34^
11	1.81 × 10^31^	2.97 × 10^25^
12	1.81 × 10^31^	2.97 × 10^25^
13	6.49 × 10^115^	2.92 × 10^25^
14	6.49 × 10^115^	2.92 × 10^25^
15	5.48 × 10^32^	1.91 × 10^25^
16	5.48 × 10^32^	1.91 × 10^25^
17	2.82 × 10^45^	2.23 × 10^20^
18	2.82 × 10^45^	2.23 × 10^20^
19	9.90 × 10^18^	4.11 × 10^15^
20	9.90 × 10^18^	4.11 × 10^15^
21	3.98 × 10^26^	2.11 × 10^14^
22	3.98 × 10^26^	2.11 × 10^14^
23	2.54 × 10^22^	1.13 × 10^14^
24	2.53 × 10^22^	1.13 × 10^14^
25	2.54 × 10^22^	1.13 × 10^14^
26	2.53 × 10^22^	1.13 × 10^14^
27	2.46 × 10^24^	2.67 × 10^13^
28	2.46 × 10^24^	2.67 × 10^13^
29	2.24 × 10^18^	2.18 × 10^13^
30	2.24 × 10^18^	2.18 × 10^13^
31	3.59 × 10^10^	5.29 × 10^8^
32	3.59 × 10^10^	5.29 × 10^8^
33	3.36 × 10^7^	2.59 × 10^5^
34	3.36 × 10^7^	2.59 × 10^5^
35	2.26 × 10^6^	1.07 × 10^5^
36	2.26 × 10^6^	1.07 × 10^5^
37	1.19 × 10^9^	1.06 × 10^5^
38	1.19 × 10^9^	1.06 × 10^5^
39	1.18 × 10^5^	1.49 × 10^3^
40	1.18 × 10^5^	1.49 × 10^3^
41	1.84 × 10^3^	510.05
42	1.84 × 10^3^	510.05
43	3.38 × 10^8^	194.54
44	3.38 × 10^8^	194.54
45	3.38 × 10^8^	194.54
46	3.38 × 10^8^	194.54
47	927.29	176.39
48	927.29	176.39
49	4.13 × 10^5^	61.12
50	4.13 × 10^5^	61.12
51	101.56	59.31
52	101.56	59.31
53	2.76 × 10^4^	58.55
54	2.76 × 10^4^	58.55
55	86.25	29.18
56	86.25	29.18
57	2.78 × 10^8^	25.51
58	2.78 × 10^8^	25.51
59	104.48	21.84
60	104.48	21.84
61	2.24 × 10^5^	17.73
62	2.24 × 10^5^	17.73
63	23.77	13.76
64	23.77	13.76
65	27.40	8.77
66	27.40	8.77
67	18.49	8.15
68	18.49	8.15
69	74.39	4.94
70	74.39	4.94
71	14.37	4.18
72	14.37	4.18
73	8.08	3.58
74	8.08	3.58
75	4.11 × 10^12^	3.51
76	4.11 × 10^12^	3.51
77	7.89	2.56
78	7.89	2.56
79	18.43	2.31
80	18.43	2.31
81	3.40	1.91
82	3.40	1.91
83	2.18	1.90
84	2.18	1.90
85	3.14	1.79
86	3.14	1.79

**Table 8:** Summary of E-Values by Anomaly.

Anomaly	Number	Mean Minimum E-Value	Median Minimum E-Value	Min Minimum E-Value	Max Minimum E-Value	Mean E-Value Estimate	Median E-Value Estimate	Min E-Value Estimate	Max E-Value Estimate
Trisomy 13	10	4.04 × 10^70^	1.91 × 10^25^	4.18	2.02 × 10^71^	4.34 × 10^128^	5.48 × 10^32^	14.37	2.17 × 10^129^
Trisomy 21	12	3.72 × 10^19^	2.65 × 10^8^	1.9	2.23 × 10^20^	4.70 × 10^44^	1.80 × 10^10^	2.18	2.82 × 10^45^
Klinefelters	10	8.446 × 10^14^	2.59 × 10^5^	3.51	4.11 × 10^15^	5.06 × 10^21^	4.11 × 10^12^	3.36 × 10^7^	2.53 × 10^22^
Chromosomes	20	1.10 × 10^306^	5.38 × 10^4^	2.31	1.10 × 10^307^	1.10 × 10^306^	1,130,920	8.08	1.10 × 10^307^
Turners	6	7.37 × 10^53^	1.49 × 10^3^	1.79	2.21 × 10^54^	3.83 × 10^98^	118,000	3.14	1.15 × 10^99^
Genetic	18	1.22 × 10^306^	61.12	1.91	1.10 × 10^307^	1.22 × 10^306^	413,000	3.4	1.10 × 10^307^
Trisomy 18	10	21,254.994	59.31	13.76	106,000	2.94 × 10^8^	927.29	23.77	1.19 × 10^9^

**Table 9:** EWAS Hits on Major Centromere Proteins; Data from Schrott [47].

Nearest Gene Name	Chromosome Number	Nearest Gene Number	Dependency Status	Functional Annotation	Page	Distance from Nearest Gene	Relative Position	*p*-Value	Bonferroni Adjusted *p*-Value
**Centrosomal Organizers**									
NUMA1	11	ENSG00000137497	Withdrawal	Nuclear Mitotic Apparatuis Protein 1	212	0	Exon	1.13 × 10^−05^	0.024154
CEP152	15	ENSG00000103995	Withdrawal	Centrosomal protein 152	195	0	Intron	7.64 × 10^−6^	0.020250
CEP55	10	ENSG00000138180	Dependence	Centrosomal protein 55	27	13,562	Downstream	6.71 × 10^−7^	0.005734
**Motor Proteins**									
**Dynein–Dynactin**									
DYNC1H1	14	ENSG00000197102	Withdrawal	Dynein cytoplasmic 1 heavy chain 1	135	0	Exon	2.69 × 10^−7^	0.003989
DYNC1H1	14	ENSG00000197102	Withdrawal	Dynein cytoplasmic 1 heavy chain 1	150	0	Intron	1.33 × 10^−6^	0.008933
DYNC1H1	14	ENSG00000197102	Withdrawal	Dynein cytoplasmic 1 heavy chain 1	186	0	Intron	6.04 × 10^−6^	0.018039
DCTN4	5	ENSG00000132912	Dependence	Dynactin subunit 4	37	0	Intron	1.38 × 10^−6^	0.008195
DCTN4	5	ENSG00000132912	Withdrawal	Dynactin subunit 4	131	327	Downstream	1.25 × 10^−7^	0.002634
DCTN4	5	ENSG00000132912	Withdrawal	Dynactin subunit 4	151	0	Intron	1.40 × 10^−6^	0.009142
DCTN4	5	ENSG00000132912	Withdrawal	Dynactin subunit 4	232	0	Intron	1.63 × 10^−5^	0.028618
**Kinesin**									
KIFAP3	1	ENSG00000075945	Dependence	Kinesin Associated protein 3	21	0	Intron	3.63 × 10^−7^	0.004266
KIF26B	1	ENSG00000162849	Dependence	Kinesin family member 26B	41	0	Intron	1.69 × 10^−6^	0.009091
KIF2A	5	ENSG00000068796	Dependence	Kinesin family member 2A	46	0	Intron	2.20 × 10^−6^	0.010274
KIF26B	1	ENSG00000162849	Dependence	Kinesin family member 26B	47	0	Intron	2.26 × 10^−6^	0.010440
KIF27	1	ENSG00000165115	Dependence	Kinesin family member 27	56	0	Exon	3.24 × 10^−6^	0.012349
KIFC3	16	ENSG00000140859	Withdrawal	Kinesin family member C3	173	0	3 Untranslated region	3.87 × 10^−6^	0.014555
KIF14	1	ENSG00000118193	Withdrawal	Kinesin family member 14	200	33,155	Downstream	8.63 × 10^−6^	0.021429
KIF13B	1	ENSG00000197892	Dependence	Kinesin family member 13B	60	0	Intron	3.76 × 10^−6^	0.013269
KIF13B	6	ENSG00000197892	Dependence	Kinesin family member 13B	77	0	Intron	6.16 × 10^−6^	0.016839
KIF3C	2	ENSG00000137497	Dependence	Kinesin family member 3C	79	0	Intron	6.44 × 10^−6^	0.017205
KIF26B	1	ENSG00000162849	Dependence	Kinesin family member 26B	83	16,530	Downstream	7.21 × 10^−6^	0.018129
KIFC2	8	ENSG00000167702	Withdrawal	Kinesin family member C2	221	0	Exon	1.35 × 10^−5^	0.026224

**Table 10:** Functional Annotations on Most Significant EWAS Hits on Kinesins from Schrott Datasets [47].

Nearest Gene Name	Page	Functional Annotation	Number Genes Identified	*p*-Value
KIF13A	239	Head and neck cancer	356	7.73 × 10^−20^
KIF26A	239	Head and neck cancer	356	7.73 × 10^−20^
KIF13A	239	Head and neck cancer	342	7.74 × 10^−20^
KIF26A	239	Head and neck cancer	342	7.74 × 10^−20^
KIF13A	240	Head and neck cancer	333	3.34 × 10^−19^
KIF26A	240	Head and neck cancer	333	3.34 × 10^−19^
KIF13A	240	Abdominal carcinoma	362	3.64 × 10^−19^
KIF26A	240	Abdominal carcinoma	362	3.64 × 10^−19^
KIF13A	241	Solid cancer	381	6.93 × 10^−18^
KIF26A	241	Solid cancer	381	6.93 × 10^−18^
KIF13A	241	Thyroid cancer	318	1.21 × 10^−17^
KIF26A	241	Thyroid cancer	318	1.21 × 10^−17^
KIF13A	242	Thyroid cancer	319	1.26 × 10^−17^
KIF26A	242	Thyroid cancer	319	1.26 × 10^−17^
KIF13A	242	Endocrine tumour	321	1.44 × 10^−17^
KIF26A	242	Endocrine tumour	321	1.44 × 10^−17^
KIF13A	243	Adenocarcinoma	351	1.91 × 10^−17^
KIF26A	243	Adenocarcinoma	351	1.91 × 10^−17^
KIF13A	243	Secretory Structure carcinoma	339	1.92 × 10^−17^
KIF26A	243	Secretory Structure carcinoma	339	1.92 × 10^−17^
KIF13A	244	Abdominal carcinoma	346	3.72 × 10^−17^
KIF26A	244	Abdominal carcinoma	346	3.72 × 10^−17^
KIF13A	244	Malignant cancer	313	3.76 × 10^−17^
KIF26A	244	Malignant cancer	313	3.76 × 10^−17^
KIF13A	245	Carcinoma	376	6.96 × 10^−17^
KIF26A	245	Carcinoma	376	6.96 × 10^−17^
KIF13A	245	Non-melanoma solid carcinoma	379	7.21 × 10^−17^
KIF26A	245	Non-melanoma solid carcinoma	379	7.21 × 10^−17^

**Table 11:** Selected EWAS Hits on Centromere Proteins (CENPs); Data from Schrott [47].

Nearest Gene Name	Chromosome	Nearest Gene	Distance to Nearest Gene	Number of Annotations	Relative Location	*p*-Value	*p*-Adjust
CENPIP1	13	ENSG00000224778	1100	1	Upstream	2.38 × 10^−9^	0.000279
CENPF	1	ENSG00000117724	72,569	3	Downstream	2.98 × 10^−8^	0.001109
CENPVL3	X	ENSG00000224109	2146	1	Downstream	2.80 × 10^−6^	0.001153
CENPK	5	ENSG00000123219	0	1	Intron	8.01 × 10^−6^	0.019098
CENPP	9	ENSG00000188312	0	2	Intron	8.26 × 10^−6^	0.019330
CENPJ	13	ENSG00000151849	0	1	Exon	4.66 × 10^−7^	0.005279
CENPUP1	11	ENSG00000255075	8401	1	Upstream	2.81 × 10^−6^	0.012567
INCENP	11	ENSG00000149503	0	1	Intron	3.07 × 10^−6^	0.013077
CENPO	2	ENSG00000138092	0	1	Exon	6.25 × 10^−6^	0.018393
CENPI	X	ENSG00000102384	0	2	Intron	7.54 × 10^−6^	0.020123
CENPL	1	ENSG00000120334	0	1	Intron	8.22 × 10^−6^	0.020943
CENPX	17	ENSG00000169689	0	1	Exon	9.35 × 10^−6^	0.022176
CENPC	4	ENSG00000145241	0	1	Intron	9.60 × 10^−6^	0.002248
CENPV	17	ENSG00000166582	13,237	2	Upstream	1.63 × 10^−5^	0.002861
CENPN	16	ENSG00000166451		86		7.73 × 10^−20^	

**Table 12:** Selected EWAS Hits on Centromere Protein N (CENPN); Data from Schrott [47].

Nearest Gene Name	Chromosome Number	Nearest Gene Number	Dependency Status	Functional Annotation	Page	Number Genes Identified	*p*-Value
CENPN	16	ENSG00000166451	Dependence	Head and Neck Cancer	239	356	7.73 × 10^−20^
CENPN	16	ENSG00000166451	Dependence	Head and Neck Cancer	239	342	7.74 × 10^−20^
CENPN	16	ENSG00000166451	Dependence	Head and Neck Cancer	240	333	3.34 × 10^−19^
CENPN	16	ENSG00000166451	Dependence	Abdominal carcinoma	240	362	3.64 × 10^−19^
CENPN	16	ENSG00000166451	Dependence	Cancer	241	381	6.93 × 10^−18^
CENPN	16	ENSG00000166451	Dependence	Thyroid carcinoma	241	318	1.21 × 10^−17^
CENPN	16	ENSG00000166451	Dependence	Secretory structure cancer	243	339	1.92 × 10^−17^
CENPN	16	ENSG00000166451	Dependence	Malignant Cancer	244	313	5.67 × 10^−17^
CENPN	16	ENSG00000166451	Dependence	Carcinoma	245	376	6.96 × 10^−17^
CENPN	16	ENSG00000166451	Dependence	Non-melanoma solid cancer	245	379	7.21 × 10^−17^
CENPN	16	ENSG00000166451	Dependence	Frequency of Tumour	246	315	7.73 × 10^−17^
CENPN	16	ENSG00000166451	Dependence	Abdominal cancer	246	364	8.91 × 10^−17^
CENPN	16	ENSG00000166451	Dependence	Endocrine gland cancer	247	327	1.38 × 10^−16^
CENPN	16	ENSG00000166451	Dependence	Cancer development	247	310	1.72 × 10^−16^
CENPN	16	ENSG00000166451	Dependence	Epithelial Tumourigenesis	248	312	1.74 × 10^−16^
CENPN	16	ENSG00000166451	Dependence	Endocrine cancer	249	323	3.74 × 10^−16^
CENPN	16	ENSG00000166451	Dependence	Gastrointestinal cancer	249	325	4.60 × 10^−16^
CENPN	16	ENSG00000166451	Dependence	Abdominal cancer	250	365	5.17 × 10^−16^
CENPN	16	ENSG00000166451	Dependence	Digestive system cancer	250	349	7.78 × 10^−16^
CENPN	16	ENSG00000166451	Dependence	Digestive system cancer	251	382	8.26 × 10^−16^

**Table 13:** EWAS Hits on RAD51/52 Recombinase; Data from Schrott [47].

Nearest Gene Name	Page	Chromosome	Status	Nearest Gene	Distance to Nearest Gene	Relative Location	*p*-Value	*p*-Adjust
**RAD51–Homologous Recombination**								
RAD51B	28	14	Dependence	ENSG00000182185	0	Intron	7.33 × 10^−7^	0.006027
RAD51B	94	14	Dependence	ENSG00000182185	0	Intron	9.09 × 10^−6^	0.020192
RAD51B	131	14	Withdrawal	ENSG00000182185	0	Intron	1.33 × 10^−7^	0.002722
RAD51B	135	14	Withdrawal	ENSG00000182185	0	Intron	2.98 × 10^−7^	0.004182
RAD51B	154	14	Withdrawal	ENSG00000182185	0	Intron	1.68 × 10^−6^	0.009923
RAD51B	158	14	Withdrawal	ENSG00000182185	0	Intron	2.13 × 10^−6^	0.000899
RAD51B	174	14	Withdrawal	ENSG00000182185	0	Intron	4.02 × 10^−6^	0.014760
RAD51B	188	14	Withdrawal	ENSG00000182185	0	Intron	6.38 × 10^−6^	0.018632
RAD51B	222	14	Withdrawal	ENSG00000182185	0	Intron	1.36 × 10^−5^	0.026286
**RAD52–Microhomology-Mediated End Joining**								
RAD52	102	12	Dependence	ENSG00000002016	0	Intron	1.07 × 10^−5^	0.021847

**Table 14:** EWAS Hits on Kinetochore Metaphase Signaling Pathway; Data from Schrott [47].

Nearest Gene Name	Page	Chromosome	Status	Nearest Gene	Distance to Nearest Gene	Relative Location	*p*-Value	*p*-Adjust
AURKAIP1	29	1	Dependence	ENSG00000175756	84	Downstream	8.14 × 10^−7^	0.006318
CCNB3	110	X	Dependence	ENSG00000175756	0	Intron	1.23 × 10^−5^	0.023286
CCNB2	154	15	Withdrawal	ENSG00000175756	0	Intron	1.60 × 10^−6^	0.009713
CCNB1IP1	159	14	Withdrawal	ENSG00000175756	0	Intron	2.13 × 10^−6^	0.011189
CDC20B	46	5	Dependence	ENSG00000164287	0	Intron	2.14 × 10^−6^	0.010145
CDC200	70	17	Dependence	ENSG00000236383	0	Intron	5.13 × 10^−6^	0.015449
CDC200	123	17	Dependence	ENSG00000236383	0	Intron	1.12 × 10^−9^	0.000216
CDC201	136	7	Withdrawal	ENSG00000283247	0	Intron	3.47 × 10^−7^	0.004589
CDC20B	213	5	Withdrawal	ENSG00000164287	18781	Upstream	1.14 × 10^−5^	0.024227
CDC200	213	17	Withdrawal	ENSG00000236383	0	Intron	1.38 × 10^−5^	0.026395
CDK1	169	10	Withdrawal	ENSG00000170312	7683	Downstream	3.41 × 10^−6^	0.013821
PPP1CC	30	12	Dependence	ENSG00000186298	0	Intron	8.18 × 10^−7^	0.006609

**Table 15:** Selected EWAS Hits on Cyclin Dependent Kinase 1; Data from Schrott [47].

Nearest Gene Name	Page	Functional Annotation	Number Genes Identified	*p*-Value
CDK1	169	Skin lesion	115	1.65 × 10^−6^
CDK1	239	Head and neck cancer	356	7.73 × 10^−20^
CDK1	325	Skin cancer	113	4.79 × 10^−6^
CDK1	325	Lung adenocarcinoma	42	5.84 × 10^−6^
CDK1	325	Tumourigenesis	149	7.17 × 10^−6^
CDK1	326	Large bowel cancer	120	7.45 × 10^−6^
CDK1	326	Cutaneous melanoma	110	7.71 × 10^−6^
CDK1	326	Abdominal adenocarcinoma	135	8.46 × 10^−6^
CDK1	327	Solid organ cancer	150	9.16 × 10^−6^
CDK1	327	Head and neck tumour	137	9.54 × 10^−6^
CDK1	327	Carcinoma	148	1.38 × 10^−5^
CDK1	328	CNS solid cancer	101	2.06 × 10^−5^
CDK1	328	Cancer	119	2.47 × 10^−5^
CDK1	328	Gastrointestinal adenocarcinoma	121	3.56 × 10^−5^

**Table 16:** Selected EWAS Hits on Cell Cycle Control of Chromosomal Replication Pathways; Data from Schrott [47].

Nearest Gene Name	Page	Chromosome	Status	Nearest Gene	Distance to Nearest Gene	Relative Location	*p*-Value	*p*-Adjust
CDK1	169	10	Withdrawal	ENSG00000170312	7683	Downstream	3.41 × 10^−6^	0.013821
CDK1	See also Table 15							
MCM6	166	2	Withdrawal	ENSG00000076003	0	Intron	2.91 × 10^−6^	0.012759
PCNA	46	20	Dependence	ENSG00000132646	0	Intron	2.11 × 10^−6^	0.012759
POLA1	121	x	Dependence	ENSG00000101868	0	Intron	1.51 × 10^−5^	0.010067
POLA1	174	x	Withdrawal	ENSG00000101868	0	Intron	3.92 × 10^−6^	0.025791
POLA1	190	x	Withdrawal	ENSG00000101868	0	Intron	6.68 × 10^−6^	0.019066
TOP2A	189	17	Withdrawal	ENSG00000131747	0	Intron	6.53 × 10^−6^	0.018855
ORC2	137	2	Withdrawal	ENSG00000170312	0	Intron	3.82 × 10^−7^	0.004841
POLA1	174	X	Withdrawal	ENSG00000170312	0	Intron	3.92 × 10^−6^	0.014634
POLA1	190	X	Withdrawal	ENSG00000170312	0	Intron	6.68 × 10^−6^	0.019066
POLA1	121	X	Dependence	ENSG00000170312	0	Intron	1.15 × 10^−4^	0.025791
TOP2A	189	17	Withdrawal	ENSG00000170312	6115	Upstream	6.53 × 10^−6^	0.018855

## Data Availability

All data generated or analyzed during this study are included in this published article and its supplementary information files.

## Supplementary Materials

The following supporting information can be downloaded at: https://www.mdpi.com/article/10.3390/ijerph191811208/s1, Table S1: Social, Demographic and Drug Use Covariates; Table S2: Daily Cannabis Use Data; Table S3: Daily Cannabis Use Data—Interpolated; Table S4: Bivariate Linear Regression Slopes; Table S5: Variable Importance Tables from Random Forrest Regression—Chromosomal Disorders; Table S6: Variable Importance Tables from Random Forrest Regression—Trisomy 21; Table S7: Variable Importance Tables from Random Forrest Regression—Trisomy 18; Table S8: Variable Importance Tables from Random Forrest Regression—Trisomy 13; Table S9: Variable Importance Tables from Random Forrest Regression—Turners syndrome; Table S10: Variable Importance Tables from Random Forrest Regression—Klinefelters syndrome; Table S10: Variable Importance Tables from Random Forrest Regression—Klinefelters syndrome; Table S12: IPW Panel Regression Models—Chromosomal Disorders; Table S13: IPW Panel Regression Models—Genetic syndromes; Table S14: IPW Panel Regression Models—Trisomy 21; Table S15: IPW Panel Regression Models—Trisomy 18; Table S16: IPW Panel Regression Models—Trisomy 13; Table S17: IPW Panel Regression Models—Turners syndrome; Table S18: IPW Panel Regression Models—Klinefelters syndrome; Table S19: Geospatial Models—Turners syndrome; Table S20: Geospatial Models—Klinefelters syndrome; Table S21: E-Values from Panel Models; Table S22: E-Values from Geospatial Models; Table S23: E-Values by Anomaly; Table S24: E-Values by Group; Table S25: Summary of E-Values by Group; Table S26: Wilcoxson Tests by Group; Table S27: Epigenetic Signals for PPP1CC; Figure S1: International geospatial links used for computing the sparse spatial weights matrix for use in the geospatial regressions (A) edited and (B) final links. Data along with the relevant R code has been made publicly available on the Mendeley Database Repository and can be accessed from these URL’s: 10.17632/mvwvxk756z.1 (accessed on 20 January 2022) and 10.17632/vd6mt5r5jm.1 (accessed on 10 January 2022).
