# Supplementary material for: Cannabis- and Substance-Related Epidemiological Patterns of Chromosomal Congenital Anomalies in Europe: Geospatiotemporal and Causal Inferential Study
Source: Int J Environ Res Public Health. 2022 Sep 6;19(18):11208. doi: 10.3390/ijerph191811208 (PMC9517644; doi:10.3390/ijerph191811208)
Supplement: Supplementary file 1 [file ijerph-19-11208-s001.zip › ijerph-1861445-supplementary.pdf]

# Supplementary Tables

## Table of Contents


| Supplementary Table Number | Table Title |
|---|---|
| | |
| S1 | Population, Drug Use and Demographic Data |
| S2 | Daily Cannabis Use - Raw Data |
| S3 | Daily Cannabis Use - Interpolated Data |
| S4 | Regression Line slopes |
| S5 | Variable Importance Tables from Ranger Random Forrest Regression - Chromosomal Disorders |
| S6 | Variable Importance Tables from Ranger Random Forrest Regression - Trisomy 21 |
| S7 | Variable Importance Tables from Ranger Random Forrest Regression - Trisomy 18 |
| S8 | Variable Importance Tables from Ranger Random Forrest Regression - Trisomy 13 |
| S9 | Variable Importance Tables from Ranger Random Forrest Regression - Turner syndrome |
| S10 | Variable Importance Tables from Ranger Random Forrest Regression - Klinefeleters Disorders |
| S11 | Variable Importance Tables from Ranger Random Forrest Regression - Genetic disorders |
| S12 | Inverse Probability Weighted Panel Regression Models - Chromosomal Disorders |
| S13 | Inverse Probability Weighted Panel Regression Models - Genetic Disorders |
| S14 | Inverse Probability Weighted Panel Regression Models - Trisomy 21 |
| S15 | Inverse Probability Weighted Panel Regression Models - Trisomy 18 |
| S16 | Inverse Probability Weighted Panel Regression Models - Trisomy 13 |
| S17 | Inverse Probability Weighted Panel Regression Models - Turner syndrome |
| S18 | Inverse Probability Weighted Panel Regression Models - Klinefelter syndrome |
| S19 | Geospatial Models - Turner syndrome |
| S20 | Geospatial Models - Klinefelter syndrome |
| S21 | E-Values from Panel Models |
| S22 | E-Values from Geospatial Models |
| S23 | E-Values ordered by anomaly |
| S24 | E-Values ordered by group of covariates |
| S25 | Summary of E-Values by group of covariates |
| S26 | Wilcoxson tests for intergroup comparison of grouped covariates |

| | |
|---|---|
| S27 | Epigenomic hits for PPP1CC from Schrott data 2021 |

**Table S1**. Population, Drug Use and Demographic Data

| Parameter | Value |
|---|---|
| | |
| Sample Size | 854 |
| Country (%) | |
| Belgium | 70 ( 8.2) |
| Bulgaria | 70 ( 8.2) |
| Croatia | 56 ( 6.6) |
| Finland | 35 ( 4.1) |
| France | 70 ( 8.2) |
| Germany | 70 ( 8.2) |
| Hungary | 21 ( 2.5) |
| Italy | 70 ( 8.2) |
| Netherlands | 63 ( 7.4) |
| Norway | 70 ( 8.2) |
| Poland | 70 ( 8.2) |
| Portugal | 70 ( 8.2) |
| Spain | 70 ( 8.2) |
| Sweden | 49 ( 5.7) |
| Year (Range) | 2000:2019 |
| Anomaly (%) | |
| Chromosomal | 122 (14.3) |
| Down Syndrome | 122 (14.3) |
| Edward syndrome/trisomy 18 | 122 (14.3) |
| Genetic syndromes + microdeletions | 122 (14.3) |
| Klinefelter syndrome | 122 (14.3) |
| Patau syndrome/trisomy 13 | 122 (14.3) |
| Turner syndrome | 122 (14.3) |

| | |
|---|---|
| Anomaly_Rate (mean (SD)) | 10.24 (14.03) |
| Tobacco (mean (SD)) | 22.76 (5.45) |
| Alcohol (p.c.L/Yr) (mean (SD)) | 10.77 (1.82) |
| Amphetamines (% Use) (median [IQR]) | 0.60 [0.30, 0.80] |
| Cocaine (% Use) (median [IQR]) | 0.71 [0.40, 1.20] |
| Last_Month_Cannabis (% Use) (mean (SD)) | 0.04 (0.02) |
| Cannabis_Herb_THC_Content (%) (mean (SD)) | 0.09 (0.04) |
| Cannabis_Resin_THC_Content (%) (mean (SD)) | 0.16 (0.09) |
| %_Daily_Cannabis_ Use (mean (SD)) | 0.01 (0.01) |
| %_Daily_Cannabis_Use_Interpolated (mean (SD)) | 0.01 (0.01) |
| Last_Month_Cannabis_x_Herb_THC_Content (mean (SD)) | 0.03 (0.03) |
| Last_Month_Cannabis_x_Resin_THC_Content (mean (SD)) | 0.13 (0.12) |
| Last_Month_Cannabis_x_Herb_THC_Content_x_Daily_Use_Interpolated (median [IQR]) | 0.02 [0.00, 0.06] |
| Last_Month_Cannabis_x_Resin_THC_Content_x_Daily_Use_Interpolated (median [IQR]) | 0.03 [0.01, 0.13] |
| Trend_in_Daily_Cannabis_Use = Increasing (%) | 609 (71.3) |
| Mean_Annual_Household_Income (mean (SD)) | 30298.66 (17315.75) |

**Table S2**. Daily Cannabis Use - Raw Data

| Country | 2010 | 2011 | 2012 | 2013 | 2014 | 2015 | 2016 | 2017 | 2018 | 2019 |
|---|---|---|---|---|---|---|---|---|---|---|
| | | | | | | | | | | |
| Belgium | - | - | - | 0.0054 | - | - | - | - | 0.0132 | - |
| Bulgaria | - | - | 0.0020 | - | - | - | 0.0016 | - | - | - |
| Croatia | - | - | 0.0050 | - | - | 0.0078 | - | - | - | - |
| Finland | 0.0020 | - | - | - | 0.0031 | - | - | - | - | - |
| France | 0.0150 | - | - | - | 0.0217 | - | - | 0.0199 | - | - |
| Germany | - | - | 0.0060 | - | - | 0.0067 | - | - | 0.0084 | - |
| Hungary | - | - | - | - | - | - | - | - | - | - |
| Italy | - | - | 0.0030 | - | 0.0094 | - | - | 0.0108 | - | - |
| Netherlands | - | 0.0146 | 0.0146 | 0.0146 | 0.0146 | 0.0146 | 0.0133 | 0.0126 | 0.0190 | 0.0200 |
| Norway | 0.0030 | 0.0030 | 0.0030 | 0.0030 | 0.0037 | 0.0044 | 0.0053 | 0.0016 | 0.0068 | 0.0038 |
| Poland | 0.0040 | - | 0.0010 | - | 0.0018 | - | - | - | - | - |
| Portugal | - | - | 0.0050 | - | - | - | 0.0002 | - | - | - |
| Spain | - | 0.0250 | - | 0.0260 | - | 0.0285 | - | 0.0372 | - | - |
| Sweden | - | - | - | - | - | - | - | - | - | - |

**Table S3**. Daily Cannabis Use - Interpolated Data

| Country | 2010 | 2011 | 2012 | 2013 | 2014 | 2015 | 2016 | 2017 | 2018 | 2019 |
|---|---|---|---|---|---|---|---|---|---|---|
| | | | | | | | | | | |
| Belgium | 0.0054 | 0.0054 | 0.0054 | 0.0054 | 0.0070 | 0.0085 | 0.0101 | 0.0116 | 0.0132 | 0.0132 |
| Bulgaria | 0.0020 | 0.0020 | 0.0020 | 0.0019 | 0.0018 | 0.0017 | 0.0016 | 0.0016 | 0.0016 | 0.0016 |
| Croatia | 0.0050 | 0.0050 | 0.0050 | 0.0059 | 0.0069 | 0.0078 | 0.0092 | 0.0107 | - | - |
| Finland | 0.0020 | 0.0023 | 0.0026 | 0.0028 | 0.0031 | - | - | - | - | - |
| France | 0.0150 | 0.0167 | 0.0184 | 0.0200 | 0.0217 | 0.0211 | 0.0205 | 0.0199 | 0.0199 | 0.0199 |
| Germany | 0.0060 | 0.0060 | 0.0060 | 0.0062 | 0.0065 | 0.0067 | 0.0073 | 0.0078 | 0.0084 | 0.0084 |
| Hungary | 0.0007 | 0.0007 | 0.0007 | - | - | - | - | - | - | - |
| Italy | 0.0030 | 0.0030 | 0.0030 | 0.0062 | 0.0094 | 0.0099 | 0.0103 | 0.0108 | 0.0108 | 0.0108 |
| Netherlands | - | 0.0146 | 0.0146 | 0.0146 | 0.0146 | 0.0146 | 0.0133 | 0.0126 | 0.0190 | 0.0200 |
| Norway | 0.0030 | 0.0030 | 0.0030 | 0.0030 | 0.0037 | 0.0044 | 0.0053 | 0.0016 | 0.0068 | 0.0038 |
| Poland | 0.0040 | 0.0025 | 0.0010 | 0.0014 | 0.0018 | 0.0018 | 0.0018 | 0.0018 | 0.0018 | 0.0018 |
| Portugal | 0.0050 | 0.0050 | 0.0050 | 0.0038 | 0.0026 | 0.0014 | 0.0002 | 0.0002 | 0.0002 | 0.0002 |
| Spain | 0.0250 | 0.0250 | 0.0255 | 0.0260 | 0.0273 | 0.0285 | 0.0329 | 0.0372 | 0.0372 | 0.0372 |
| Sweden | - | - | - | - | - | - | - | - | - | - |

**Table S4**. Regression Line Slopes

| Anomaly | Substance | Mean Anomaly Rate | Estimate | Std.Error | Sigma | t_statistic | *p*_Value | E-Value Estimate | E-Value Lower Bound |
|---|---|---|---|---|---|---|---|---|---|
| | | | | | | | | | |
| Genetic syndromes | Daily.Interpol. | 5.9209 | 54.6837 | 9.3918 | 0.8815 | 5.8225 | 5.53E-08 | 6.55E+24 | 3.80642E+16 |
| Edward syndrome | Daily.Interpol. | 4.9688 | 39.6511 | 8.1134 | 0.7616 | 4.8871 | 3.41E-06 | 7.55E+20 | 4.38575E+12 |
| Chromosomal | Daily.Interpol. | 35.8047 | 23.0358 | 5.0643 | 0.4754 | 4.5487 | 1.37E-05 | 2.84E+19 | 1.64891E+11 |
| Down Syndrome | Daily.Interpol. | 20.8129 | 19.0578 | 4.4071 | 0.4137 | 4.3244 | 3.31E-05 | 3.23E+18 | 1.87E+10 |
| Turner syndrome | Daily.Interpol. | 1.7968 | 27.0000 | 7.2926 | 0.6845 | 3.7024 | 3.32E-04 | 7.76E+15 | 4.51E+07 |
| Klinefelter | Daily.Interpol. | 0.6084 | 18.2674 | 5.0311 | 0.4722 | 3.6309 | 4.26E-04 | 3.88E+15 | 2.25E+07 |
| Patau syndrome | Daily.Interpol. | 1.7793 | 24.1206 | 7.0551 | 0.6622 | 3.4189 | 8.75E-04 | 4.97E+14 | 2.89E+06 |
| Genetic syndromes | LMCannabis_Herb | 5.9209 | 15.6419 | 3.1034 | 0.8908 | 5.0402 | 1.66E-06 | 1.74E+07 | 3.53E+04 |
| Genetic syndromes | LM_Cannabis | 5.9209 | 15.5798 | 3.7939 | 0.9182 | 4.1065 | 7.37E-05 | 1.02E+07 | 6.50E+03 |
| Genetic syndromes | Herb | 5.9209 | 10.0478 | 2.3492 | 0.9135 | 4.2771 | 3.82E-05 | 4.45E+04 | 456.72 |
| Chromosomal | LMCannabis_Herb | 35.8047 | 6.2955 | 1.7442 | 0.5007 | 3.6095 | 4.49E-04 | 1.86E+05 | 377.43 |
| Edward syndrome | LMCannabis_Herb | 4.9688 | 9.7114 | 2.7935 | 0.8019 | 3.4764 | 7.08E-04 | 1.22E+05 | 247.40 |
| Down Syndrome | LMCannabis_Herb | 20.8129 | 5.2012 | 1.5292 | 0.4390 | 3.4013 | 9.12E-04 | 9.63E+04 | 194.83 |
| Klinefelter | LMCannabis_Herb | 0.6084 | 5.2172 | 1.6420 | 0.4713 | 3.1774 | 0.0019 | 4.74E+04 | 95.58 |
| Down Syndrome | LM.Cannabis_x_Herb.THC_x_Daily.Interpol. | 20.8129 | 2.3272 | 0.4751 | 0.4056 | 4.8985 | 3.25E-06 | 369.89 | 45.54 |
| Chromosomal | LM.Cannabis_x_Herb.THC_x_Daily.Interpol. | 35.8047 | 2.6195 | 0.5532 | 0.4723 | 4.7349 | 6.41E-06 | 310.61 | 38.17 |
| Edward syndrome | LM_Cannabis | 4.9688 | 9.1559 | 3.3741 | 0.8166 | 2.7136 | 0.0076 | 5.40E+04 | 34.01 |
| Edward syndrome | LM.Cannabis_x_Herb.THC_x_Daily.Interpol. | 4.9688 | 4.1359 | 0.9014 | 0.7696 | 4.5883 | 1.16E-05 | 265.61 | 32.57 |
| Klinefelter | LM_Cannabis | 0.6084 | 5.2968 | 1.9692 | 0.4766 | 2.6898 | 0.0082 | 4.94E+04 | 31.06 |
| Genetic syndromes | LM.Cannabis_x_Herb.THC_x_Daily.Interpol. | 5.9209 | 4.8225 | 1.0864 | 0.9275 | 4.4389 | 2.11E-05 | 226.42 | 27.70 |

| | | | | | | | | | |
|---|---|---|---|---|---|---|---|---|---|
| Patau syndrome | LM.Cannabis_x_Herb.THC_x_Daily.Interpol. | 1.7793 | 2.9541 | 0.7660 | 0.6539 | 3.8567 | 1.92E-04 | 121.51 | 14.65 |
| Turner syndrome | LMCannabis_Herb | 1.7968 | 6.2946 | 2.4593 | 0.7060 | 2.5595 | 0.0117 | 6.68E+03 | 13.03 |
| Klinefelter | LM.Cannabis_x_Herb.THC_x_Daily.Interpol. | 0.6084 | 2.0130 | 0.5530 | 0.4721 | 3.6401 | 4.12E-04 | 96.35 | 11.52 |
| Chromosomal | LM.Cannabis_x_Resin.THC_x_Daily.Interpol. | 35.8047 | 1.0375 | 0.1974 | 0.3694 | 5.2563 | 8.60E-07 | 25.26 | 9.43 |
| Edward syndrome | LM.Cannabis_x_Resin.THC_x_Daily.Interpol. | 4.9688 | 1.7864 | 0.3612 | 0.6758 | 4.9465 | 3.13E-06 | 21.66 | 8.03 |
| Genetic syndromes | LMCannabis_Resin | 5.9209 | 2.6736 | 0.7047 | 0.8438 | 3.7940 | 2.48E-04 | 35.24 | 7.55 |
| Turner syndrome | LM.Cannabis_x_Herb.THC_x_Daily.Interpol. | 1.7968 | 2.6440 | 0.8118 | 0.6930 | 3.2570 | 0.0015 | 63.88 | 7.47 |
| Down Syndrome | LM.Cannabis_x_Resin.THC_x_Daily.Interpol. | 20.8129 | 0.8706 | 0.1835 | 0.3434 | 4.7435 | 7.14E-06 | 19.57 | 7.22 |
| Chromosomal | LMCannabis_Resin | 35.8047 | 1.2280 | 0.3399 | 0.4070 | 3.6129 | 4.66E-04 | 30.64 | 6.50 |
| Edward syndrome | LMCannabis_Resin | 4.9688 | 2.1258 | 0.5980 | 0.7160 | 3.5549 | 5.68E-04 | 29.30 | 6.20 |
| Chromosomal | Resin | 35.8047 | 1.4387 | 0.4591 | 0.4127 | 3.1340 | 0.0022 | 47.23 | 6.05 |
| Chromosomal | Herb | 35.8047 | 3.2385 | 1.3231 | 0.5145 | 2.4478 | 0.0158 | 614.55 | 5.77 |
| Genetic syndromes | LM.Cannabis_x_Resin.THC_x_Daily.Interpol. | 5.9209 | 1.8703 | 0.4563 | 0.8539 | 4.0989 | 8.57E-05 | 14.16 | 5.12 |
| Patau syndrome | LMCannabis_Herb | 1.7793 | 5.4238 | 2.4275 | 0.6968 | 2.2343 | 0.0273 | 2.38E+03 | 4.27 |
| Patau syndrome | LM.Cannabis_x_Resin.THC_x_Daily.Interpol. | 1.7793 | 1.2368 | 0.3458 | 0.6472 | 3.5761 | 5.44E-04 | 10.86 | 3.82 |
| Genetic syndromes | Log(Cocaine) | 5.9209 | 0.7636 | 0.1002 | 0.8051 | 7.6174 | 6.61E-12 | 4.17 | 3.21 |
| Down Syndrome | LMCannabis_Resin | 20.8129 | 0.8596 | 0.3201 | 0.3833 | 2.6853 | 0.0084 | 14.88 | 2.88 |
| Turner syndrome | LM.Cannabis_x_Resin.THC_x_Daily.Interpol. | 1.7968 | 1.1097 | 0.3598 | 0.6732 | 3.0845 | 0.0026 | 8.43 | 2.86 |
| Edward syndrome | Log(Cocaine) | 4.9688 | 0.5400 | 0.0924 | 0.7423 | 5.8418 | 4.52E-08 | 3.29 | 2.48 |
| Chromosomal | Log(Cocaine) | 35.8047 | 0.3167 | 0.0589 | 0.4732 | 5.3748 | 3.83E-07 | 3.08 | 2.31 |
| Down Syndrome | Log(Cocaine) | 20.8129 | 0.2455 | 0.0527 | 0.4229 | 4.6615 | 8.20E-06 | 2.78 | 2.06 |
| Klinefelter | LM.Cannabis_x_Resin.THC_x_Daily.Interpol. | 0.6084 | 0.6802 | 0.2641 | 0.4941 | 2.5759 | 0.0115 | 6.46 | 2.04 |

| | | | | | | | | | |
|---|---|---|---|---|---|---|---|---|---|
| Turner syndrome | LM.Cannabis_x_Herb.THC: LM.Cannabis_x_Resin.THC_x_Daily.Interpol. | 1.7968 | 0.3768 | 0.0835 | 0.6703 | 4.5142 | 1.49E-05 | 2.72 | 2.01 |
| Down Syndrome | Herb | 20.8129 | 2.4157 | 1.1613 | 0.4516 | 2.0801 | 0.0396 | 259.68 | 2.01 |
| Klinefelter | Log(Cocaine) | 0.6084 | 0.2203 | 0.0577 | 0.4634 | 3.8186 | 2.14E-04 | 2.45 | 1.77 |
| Genetic syndromes | Resin | 5.9209 | 2.1135 | 0.9795 | 0.8805 | 2.1578 | 0.0332 | 17.26 | 1.75 |
| Patau syndrome | Log(Cocaine) | 1.7793 | 0.2952 | 0.0844 | 0.6774 | 3.4991 | 6.56E-04 | 2.34 | 1.67 |
| Turner syndrome | Herb | 1.7968 | 3.7174 | 1.8333 | 0.7129 | 2.0277 | 0.0448 | 229.61 | 1.65 |
| Down Syndrome | Resin | 20.8129 | 0.8844 | 0.4322 | 0.3886 | 2.0460 | 0.0433 | 15.35 | 1.42 |
| Edward syndrome | Resin | 4.9688 | 1.6669 | 0.8273 | 0.7437 | 2.0149 | 0.0465 | 14.86 | 1.32 |
| Turner syndrome | Resin | 1.7968 | 1.1661 | 0.5756 | 0.6892 | 2.0259 | 0.0453 | 8.80 | 1.29 |
| Patau syndrome | Log(Amphetamine) | 1.7793 | 0.0005 | 0.0892 | 0.7112 | 0.0052 | 0.9959 | 1.02 | 1 |
| Genetic syndromes | Log(Amphetamine) | 5.9209 | 0.0486 | 0.1229 | 0.9800 | 0.3954 | 0.6933 | 1.27 | 1 |
| Klinefelter | LMCannabis_Resin | 0.6084 | 0.3446 | 0.4157 | 0.4977 | 0.8289 | 0.4090 | 3.16 | 1 |
| Patau syndrome | Resin | 1.7793 | 0.8450 | 0.7766 | 0.6981 | 1.0881 | 0.2790 | 5.47 | 1 |
| Patau syndrome | LMCannabis_Resin | 1.7793 | 0.9364 | 0.5792 | 0.6935 | 1.6168 | 0.1089 | 6.29 | 1 |
| Klinefelter | Herb | 0.6084 | 0.7205 | 1.2604 | 0.4901 | 0.5717 | 0.5686 | 7.08 | 1 |
| Turner syndrome | Resin | 1.7968 | 1.1884 | 0.7729 | 0.6948 | 1.5376 | 0.1272 | 8.96 | 1 |
| Edward syndrome | Herb | 4.9688 | 2.7375 | 2.1491 | 0.8357 | 1.2737 | 0.2052 | 38.91 | 1 |
| Patau syndrome | LM_Cannabis | 1.7793 | 2.4006 | 2.9302 | 0.7092 | 0.8192 | 0.4143 | 43.03 | 1 |
| Patau syndrome | Herb | 1.7793 | 2.5272 | 1.8143 | 0.7055 | 1.3929 | 0.1662 | 51.59 | 1 |
| Turner syndrome | LM_Cannabis | 1.7968 | 4.5985 | 2.9659 | 0.7178 | 1.5505 | 0.1237 | 679.99 | 1 |
| Down Syndrome | LM_Cannabis | 20.8129 | 3.2556 | 1.8757 | 0.4540 | 1.7356 | 0.0852 | 1.36E+03 | 1 |
| Chromosomal | LM_Cannabis | 35.8047 | 3.9780 | 2.1476 | 0.5198 | 1.8523 | 0.0664 | 2.12E+03 | 1 |
| Genetic syndromes | Annual_Alcohol | 5.9209 | -0.0140 | 0.0488 | 0.9803 | -0.2872 | 0.7745 | 1.13 | NA |
| Klinefelter | Tobacco | 0.6084 | -0.0073 | 0.0081 | 0.4891 | -0.8950 | 0.3726 | 1.13 | NA |
| Turner syndrome | Annual_Alcohol | 1.7968 | -0.0109 | 0.0360 | 0.7247 | -0.3027 | 0.7627 | 1.13 | NA |
| Genetic syndromes | Tobacco | 5.9209 | -0.0166 | 0.0162 | 0.9764 | -1.0225 | 0.3086 | 1.14 | NA |
| Down Syndrome | Tobacco | 20.8129 | -0.0116 | 0.0076 | 0.4552 | -1.5347 | 0.1275 | 1.18 | NA |

| | | | | | | | | | |
|---|---|---|---|---|---|---|---|---|---|
| Edward syndrome | Log(Amphetamine) | 4.9688 | -0.0244 | 0.1055 | 0.8411 | -0.2310 | 0.8177 | 1.19 | NA |
| Chromosomal | Tobacco | 35.8047 | -0.0198 | 0.0086 | 0.5159 | -2.3037 | 0.0230 | 1.23 | NA |
| Patau syndrome | Tobacco | 1.7793 | -0.0269 | 0.0116 | 0.6957 | -2.3209 | 0.0220 | 1.23 | NA |
| Turner syndrome | Tobacco | 1.7968 | -0.0377 | 0.0116 | 0.6949 | -3.2594 | 0.0015 | 1.28 | NA |
| Patau syndrome | Annual_Alcohol | 1.7793 | -0.0425 | 0.0352 | 0.7069 | -1.2083 | 0.2293 | 1.30 | NA |
| Edward syndrome | Tobacco | 4.9688 | -0.0504 | 0.0132 | 0.7945 | -3.8142 | 2.17E-04 | 1.31 | NA |
| Chromosomal | Log(Amphetamine) | 35.8047 | -0.0368 | 0.0660 | 0.5265 | -0.5574 | 0.5783 | 1.33 | NA |
| Chromosomal | Annual_Alcohol | 35.8047 | -0.0381 | 0.0260 | 0.5225 | -1.4672 | 0.1449 | 1.34 | NA |
| Down Syndrome | Log(Amphetamine) | 20.8129 | -0.0340 | 0.0576 | 0.4590 | -0.5908 | 0.5558 | 1.34 | NA |
| Klinefelter | Annual_Alcohol | 0.6084 | -0.0451 | 0.0241 | 0.4837 | -1.8751 | 0.0632 | 1.40 | NA |
| Edward syndrome | Daily.Interpol. | 4.9688 | -0.0784 | 0.0412 | 0.8289 | -1.9012 | 0.0597 | 1.40 | NA |
| Down Syndrome | Annual_Alcohol | 20.8129 | -0.0450 | 0.0225 | 0.4521 | -2.0023 | 0.0475 | 1.42 | NA |
| Turner syndrome | Log(Amphetamine) | 1.7968 | -0.1577 | 0.0898 | 0.7158 | -1.7572 | 0.0814 | 1.74 | NA |
| Klinefelter | Log(Amphetamine) | 0.6084 | -0.1347 | 0.0603 | 0.4809 | -2.2331 | 0.0274 | 1.90 | NA |
| Klinefelter | Resin | 0.6084 | -1.0190 | 0.5465 | 0.4913 | -1.8645 | 0.0650 | 12.68 | NA |

**Table S5**. Variable Importance Tables from Ranger Random Forrest Regression

- Chromosomal Disorders

| Variable | Importance |
|---|---|
| | |
| Income | 7.1881 |
| LM.Cannabis_x_Resin.THC_x_Daily.Interpol. | 3.5107 |
| LM.Cannabis_x_Resin.THC | 3.2384 |
| Daily.Interpol. | 2.6239 |
| LM.Cannabis_x_Herb.THC_x_Daily.Interpol. | 2.2387 |
| Tobacco | 2.2005 |
| Daily.Interpol. | 2.1287 |
| Resin | 1.9307 |
| LM.Cannabis_x_Herb.THC | 1.8777 |
| Herb | 1.7375 |
| LM.Cannabis | 1.1745 |
| Alcohol | 1.1534 |
| Amphetamines | 0.7594 |

**Table S6**. Variable Importance Tables from Ranger Random Forrest Regression

- Trisomy 21

| Variable | Importance |
|---|---|
| | |
| Income | 3.9949 |
| Cocaine | 2.3970 |
| LM.Cannabis_x_Resin.THC | 2.3596 |
| LM.Cannabis_x_Resin.THC_x_Daily.Interpol. | 2.2159 |
| LM.Cannabis_x_Herb.THC_x_Daily.Interpol. | 2.2156 |
| LM.Cannabis_x_Herb.THC | 1.9707 |
| Herb | 1.6507 |
| Daily.Interpol. | 1.6045 |
| Resin | 1.4312 |
| Tobacco | 1.3743 |
| Alcohol | 1.0998 |
| LM.Cannabis | 1.0771 |
| Amphetamines | 0.6674 |

**Table S7**. Variable Importance Tables from Ranger Random Forrest Regression

- Trisomy 18

| Variable | Importance |
|---|---|
| | |
| Income | 19.8726 |
| Tobacco | 7.9246 |
| Daily.Interpol. | 7.8947 |
| Cocaine | 7.5654 |
| LM.Cannabis_x_Resin.THC_x_Daily.Interpol. | 7.0033 |
| LM.Cannabis_x_Resin.THC | 5.8536 |
| LM.Cannabis_x_Herb.THC_x_Daily.Interpol. | 5.2141 |
| Resin | 4.9771 |
| LM.Cannabis_x_Herb.THC | 3.9041 |
| Alcohol | 3.8005 |
| Herb | 3.5019 |
| LM.Cannabis | 2.4866 |
| Amphetamines | 1.7892 |

**Table S8**. Variable Importance Tables from Ranger Random Forrest Regression

- Trisomy 13

| Variable | Importance |
|---|---|
| | |
| Income | 9.4436 |
| Tobacco | 5.6421 |
| LM.Cannabis_x_Herb.THC | 5.3712 |
| Daily.Interpol. | 4.9279 |
| LM.Cannabis_x_Resin.THC_x_Daily.Interpol. | 4.1905 |
| LM.Cannabis_x_Herb.THC_x_Daily.Interpol. | 4.0741 |
| LM.Cannabis | 3.9544 |
| Cocaine | 3.9335 |
| Herb | 3.7794 |
| Resin | 3.6753 |
| LM.Cannabis_x_Herb.THC: LM.Cannabis_x_Resin.THC_x_Daily.Interpol. | 3.5738 |
| Amphetamines | 2.3969 |
| Alcohol | 1.9779 |

**Table S9.** Variable Importance Tables from Ranger Random Forrest Regression

- Turner syndrome

| Variable | Importance |
| --- | --- |
| | |
| Resin | 12.5864 |
| Tobacco | 7.5239 |
| Daily.Interpol. | 5.3667 |
| Cocaine | 5.1068 |
| LM.Cannabis_x_Resin.THC_x_Daily.Interpol. | 4.8616 |
| LM.Cannabis_x_Resin.THC | 3.8699 |
| LM.Cannabis_x_Herb.THC_x_Daily.Interpol. | 3.4642 |
| Amphetamines | 3.4171 |
| Alcohol | 3.1727 |
| Herb | 2.8898 |
| Resin | 2.6318 |
| LM.Cannabis_x_Herb.THC | 2.3259 |
| LM.Cannabis | 1.9035 |

**Table S10**. Variable Importance Tables from Ranger Random Forrest Regression

- Klinefelters syndrome

| Variable | Importance |
|---|---|
| | |
| Income | 3.1401 |
| Tobacco | 2.6523 |
| Daily.Interpol. | 2.5976 |
| LM.Cannabis_x_Herb.THC | 2.4446 |
| LM.Cannabis_x_Herb.THC_x_Daily.Interpol. | 2.3847 |
| Amphetamines | 2.2584 |
| LM.Cannabis | 2.1803 |
| LM.Cannabis_x_Resin.THC_x_Daily.Interpol. | 1.9092 |
| Alcohol | 1.6953 |
| Cocaine | 1.6753 |
| Resin | 1.5971 |
| Herb | 1.2411 |
| LM.Cannabis_x_Resin.THC | 1.1098 |

**Table S11**. Variable Importance Tables from Ranger Random Forrest Regression

- Genetic disorders

| Variable | Importance |
| --- | --- |
| | |
| Income | 3.1503 |
| Tobacco | 2.5964 |
| Daily.Interpol. | 2.5912 |
| LM.Cannabis_x_Herb.THC_x_Daily.Interpol. | 2.5331 |
| LM.Cannabis_x_Herb.THC | 2.5068 |
| Amphetamines | 2.2876 |
| LM.Cannabis | 2.1333 |
| Cocaine | 1.8418 |
| Alcohol | 1.7553 |
| LM.Cannabis_x_Resin.THC_x_Daily.Interpol. | 1.7461 |
| Resin | 1.4309 |
| Herb | 1.1989 |
| LM.Cannabis_x_Resin.THC | 1.1962 |

**Table S12**. Inverse Probability Weighted Panel Regression Models

- Chromosomal Disorders

| Parameters | | | Model Parameters | |
|---|---|---|---|---|
| **Term** | **Estimate (C.I.)** | ***p*-Value** | **Parameter** | **Value** |
| | | | | |
| ***Additive*** | | | | |
| *(Rate ~ Tobacco + Alcohol + LM.Cannabis_x_Herb.THC_x_Daily.Interpol. + LM.Cannabis_x_Resin.THC_x_Daily.Interpol. + Daily.Interpol. + LM.Cannabis_x_Resin.THC + Amphetamines + Cocaine + Income)* | | | | |
| Tobacco | 0.04 (0.01, 0.07) | 0.0056 | Adj.R.Squared | 0.3619 |
| Alcohol | −0.07 (−0.14, −0.01) | 0.0258 | Statistic | 50.3509 |
| Daily.Interpol. | 29.9 (16.26, 43.54) | 3.61E-05 | Deg.Freedom | 6,115 |
| LM.Cannabis_x_Resin.THC | 1.22 (0.8, 1.64) | 1.36E-07 | *p*-Value | <2.2E-16 |
| Cocaine | −0.37 (−0.59, −0.16) | 0.0009 | | |
| Income | 0 (0, 0) | <2.2E-16 | | |
| | | | | |
| ***Interactive*** | | | | |
| *(Rate ~ Tobacco * Daily.Interpol. + LM.Cannabis_x_Resin.THC * LM.Cannabis_x_Resin.THC_x_Daily.Interpol. + LM.Cannabis_x_Herb.THC_x_Daily.Interpol. + Alcohol + Amphetamines + Cocaine + Income)* | | | | |
| Tobacco | 0.09 (0.06, 0.13) | 6.45E-07 | Adj.R.Squared | 0.3832 |
| Daily.Interpol. | 257 (179.58, 334.42) | 2.22E-09 | Statistic | 49.1932 |
| LM.Cannabis_x_Resin.THC | 1.7 (0.48, 2.92) | 0.0077 | Deg.Freedom | 9,112 |
| LM.Cannabis_x_Resin.THC_x_Daily.Interpol. | −1.27 (−2.28, −0.26) | 0.0147 | *p*-Value | <2.2E−16 |
| LM.Cannabis_x_Herb.THC_x_Daily.Interpol. | 1.05 (0.18, 1.92) | 0.0198 | | |
| Amphetamines | −0.25 (−0.38, −0.12) | 0.0003 | | |
| Cocaine | −0.27 (−0.47, −0.07) | 0.0090 | | |
| Income | 0 (0, 0) | 1.89E-10 | | |

| | | | | |
|---|---|---|---|---|
| Tobacco: Daily.Interpol. | −9.1 (−12.14, −6.06) | 4.36E-08 | | |
| | | | | |
| ***1 Lag*** | | | | |
| *(Rate ~ Tobacco,1) * Daily.Interpol.,1) + LM.Cannabis_x_Resin.THC,1) * LM.Cannabis_x_Resin.THC_x_Daily.Interpol. + LM.Cannabis_x_Herb.THC_x_Daily.Interpol.,1) + Alcohol,1) + Amphetamines,1) + Cocaine,1) + Income,1))* | | | | |
| Daily.Interpol. | 18.2 (13.14, 23.26) | 2.29E-10 | Adj.R.Squared | 0.4246 |
| LM.Cannabis_x_Resin.THC | 2.98 (2.42, 3.54) | <2.2E-16 | Statistic | 85.0993 |
| LM.Cannabis_x_Resin.THC_x_Daily.Interpol. | −0.86 (−1.24, −0.48) | 1.96E-05 | Deg.Freedom | 6,101 |
| LM.Cannabis_x_Herb.THC_x_Daily.Interpol. | 0.64 (0.32, 0.96) | 0.0002 | *p*-Value | <2.2E-16 |
| Amphetamines | −0.17 (−0.28, −0.05) | 0.0047 | | |
| Income | 0 (0, 0) | 4.07E-07 | | |
| | | | | |
| ***2 Lags*** | | | | |
| *(Rate ~ Tobacco + Daily.Interpol. * LM.Cannabis_x_Resin.THC * LM.Cannabis_x_Resin.THC_x_Daily.Interpol. + LM.Cannabis_x_Herb.THC_x_Daily.Interpol. + Alcohol + Amphetamines + Cocaine + Income)* | | | | |
| Daily.Interpol. | 34.6 (28.13, 41.07) | <2.2E-16 | Adj.R.Squared | 0.3362 |
| LM.Cannabis_x_Resin.THC | 6.76 (5.28, 8.24) | 5.57E-14 | Statistic | 64.3312 |
| LM.Cannabis_x_Resin.THC_x_Daily.Interpol. | −0.13 (−0.18, −0.08) | 9.90E-07 | Deg.Freedom | 6,87 |
| Amphetamines | −0.26 (−0.43, −0.1) | 0.0020 | *p*-Value | <2.2E-16 |
| Income | 0 (0, 0) | 0.0100 | | |
| Daily.Interpol.: LM.Cannabis_x_Resin.THC | −162 (−212.76, −111.24) | 1.38E-08 | | |

**Table S13**. Inverse Probability Weighted Panel Regression Models

- Genetic Disorders

| Parameters | | | Model Parameters | |
|---|---|---|---|---|
| **Term** | **Estimate (C.I.)** | ***p*-Value** | **Parameter** | **Value** |
| | | | | |
| ***Additive*** | | | | |
| *(Rate ~ Tobacco * LM.Cannabis_x_Herb.THC + Daily.Interpol. + LM.Cannabis + Alcohol + LM.Cannabis_x_Herb.THC_x_Daily.Interpol. + Amphetamines + Cocaine + Income)* | | | | |
| LM.Cannabis_x_Herb.THC | 24.9 (15.47, 34.32) | 9.55E-07 | Adj.R.Squared | 0.1412 |
| Daily.Interpol. | 9.27 (2.23, 16.31) | 0.0111 | Statistic | 73.6793 |
| LM.Cannabis_x_Herb.THC_x_Daily.Interpol. | −0.07 (−0.13, −0.02) | 0.0068 | Deg.Freedom | 5,116 |
| Amphetamines | −0.19 (−0.31, −0.08) | 0.0015 | *p*-Value | <2.2E-16 |
| Tobacco: LM.Cannabis_x_Herb.THC | −0.72 (−1.15, −0.28) | 0.0015 | | |
| | | | | |
| ***Interactive*** | | | | |
| *(Rate ~ Tobacco * Daily.Interpol. + LM.Cannabis_x_Herb.THC + LM.Cannabis_x_Resin.THC_x_Daily.Interpol. + LM.Cannabis_x_Herb.THC_x_Daily.Interpol. + Alcohol + Amphetamines + Cocaine + Income)* | | | | |
| Tobacco | 0.14 (0.09, 0.19) | 8.32E-08 | Adj.R.Squared | 0.2602 |
| Daily.Interpol. | 545 (421.13, 668.87) | 4.38E-14 | Statistic | 49.9543 |
| LM.Cannabis_x_Herb.THC | 9.5 (2.64, 16.36) | 0.0076 | Deg.Freedom | 8,113 |
| LM.Cannabis_x_Resin.THC_x_Daily.Interpol. | −2.34 (−3.32, −1.36) | 8.86E-06 | *p*-Value | <2.2E-16 |
| LM.Cannabis_x_Herb.THC_x_Daily.Interpol. | 2.05 (1.2, 2.9) | 7.63E-06 | | |
| Cocaine | −0.5 (−0.82, −0.18) | 0.0024 | | |
| Income | 0 (0, 0) | 4.16E-06 | | |
| Tobacco: Daily.Interpol. | −19.7 (−24.46, −14.94) | 7.56E-13 | | |
| | | | | |
| ***1 Lag*** | | | | |

| | | | | |
|---|---|---|---|---|
| *(Rate ~ Tobacco,1) * LM.Cannabis_x_Herb.THC ,1) +Daily.Interpol.,1) + LM.Cannabis_x_Resin.THC_x_Daily.Interpol. ,1) + LM.Cannabis_x_Herb.THC_x_Daily.Interpol. ,1) + Alcohol,1) + Amphetamines,1) + Cocaine,1) + Income,1))* | | | | |
| Daily.Interpol. | −34.09 (−60.17, −8.02) | 0.0119 | Adj.R.Squared | 0.2975 |
| LM.Cannabis_x_Resin.THC_x_Daily.Interpol. | 1.23 (0.39, 2.06) | 0.0047 | Statistic | 56.0412 |
| LM.Cannabis_x_Herb.THC_x_Daily.Interpol. | −1.1 (−1.83, −0.38) | 0.0036 | Deg.Freedom | 5,102 |
| Cocaine | 0.63 (0.31, 0.95) | 0.0002 | *p*-Value | <2.2E-16 |
| Tobacco: LM.Cannabis_x_Herb.THC | 0.59 (0.22, 0.96) | 0.0023 | | |
| | | | | |
| ***2 Lags*** | | | | |
| *(Rate ~ Tobacco * Daily.Interpol. + LM.Cannabis_x_Herb.THC +LM.Cannabis_x_Resin.THC_x_Daily.Interpol. + LM.Cannabis_x_Herb.THC_x_Daily.Interpol. + Alcohol + Amphetamines + Cocaine + Income)* | | | | |
| Tobacco | 0.07 (0.02, 0.12) | 0.0092 | Adj.R.Squared | 0.2558 |
| Daily.Interpol. | 271.11 (58.63, 483.59) | 0.0142 | Statistic | 30.9479 |
| LM.Cannabis_x_Herb.THC | 11.85 (2.23, 21.47) | 0.0178 | Deg.Freedom | 5,88 |
| Cocaine | 0.56 (0.12, 0.99) | 0.0133 | *p*-Value | <2.2E-16 |
| Tobacco: Daily.Interpol. | −11.57 (−19.62, −3.53) | 0.0060 | | |

**Table S14**. Inverse Probability Weighted Panel Regression Models

- Trisomy 21

| Parameters | | | Model Parameters | |
|---|---|---|---|---|
| **Term** | **Estimate (C.I.)** | ***p*-Value** | **Parameter** | **Value** |
| | | | | |
| ***Additive*** | | | | |
| *(Rate ~ Tobacco + Alcohol + LM.Cannabis_x_Resin.THC_x_Daily.Interpol. + LM.Cannabis_x_Herb.THC_x_Daily.Interpol. + LM.Cannabis_x_Herb.THC + LM.Cannabis_x_Herb.THC + Amphetamines + Cocaine + Income)* | | | | |
| Tobacco | 0.03 (0.01, 0.05) | 0.0121 | Adj.R.Squared | 0.3657 |
| Alcohol | −0.07 (−0.12, −0.01) | 0.016 | Statistic | 65.8289 |
| LM.Cannabis_x_Herb.THC_x_Daily.Interpol. | −0.06 (−0.11, −0.01) | 0.0175 | Deg.Freedom | 5,116 |
| LM.Cannabis_x_Herb.THC | 10.3 (9.12, 11.48) | <2.2E-16 | *p*-Value | <2.2E-16 |
| Income | 0 (0, 0) | 6.50E-05 | | |
| | | | | |
| ***Interactive*** | | | | |
| *(Rate ~ Tobacco * LM.Cannabis_x_Herb.THC_x_Daily.Interpol. * LM.Cannabis_x_Herb.THC + LM.Cannabis_x_Resin.THC_x_Daily.Interpol. * LM.Cannabis_x_Resin.THC + Alcohol + Herb + Amphetamines + Cocaine + Income)* | | | | |
| Tobacco | 0.11 (0.07, 0.15) | 7.82E-07 | Adj.R.Squared | 0.3011 |
| LM.Cannabis_x_Herb.THC_x_Daily.Interpol. | −1.56 (−2.13, −0.99) | 4.95E-07 | Statistic | 49.292 |
| LM.Cannabis_x_Herb.THC | 43.5 (19.2, 67.8) | 0.000628 | Deg.Freedom | 9,112 |
| LM.Cannabis_x_Resin.THC_x_Daily.Interpol. | 1.66 (1.01, 2.31) | 2.06E-06 | *p*-Value | <2.2E-16 |
| Amphetamines | −0.16 (−0.27, −0.05) | 0.005613 | | |
| Income | 0 (0, 0) | 3.91E-11 | | |
| Tobacco: LM.Cannabis_x_Herb.THC | −1.85 (−2.86, −0.84) | 0.000507 | | |
| LM.Cannabis_x_Herb.THC_x_Daily.Interpol.: LM.Cannabis_x_Herb.THC | 25.4 (13.54, 37.26) | 5.56E-05 | | |
| LM.Cannabis_x_Resin.THC_x_Daily.Interpol.: LM.Cannabis_x_Resin.THC | −3.09 (−4.18, −2) | 2.04E-07 | | |

| | | | | |
|---|---|---|---|---|
| | | | | |
| ***2 Lags*** | | | | |
| *(Rate ~ Tobacco + LM.Cannabis_x_Herb.THC_x_Daily.Interpol. * LM.Cannabis_x_Herb.THC * LM.Cannabis_x_Resin.THC_x_Daily.Interpol. + Alcohol + Amphetamines + Cocaine + Income)* | | | | |
| LM.Cannabis_x_Herb.THC: LM.Cannabis_x_Resin.THC_x_Daily.Interpol. | 1.93 (1.38, 2.48) | 8.48E-10 | Adj.R.Squared | 0.244 |
| Amphetamines | −0.22 (−0.37, −0.07) | 0.005674 | Statistic | 48.8577 |
| Cocaine | 0.21 (0.12, 0.29) | 5.29E-06 | Deg.Freedom | 5,88 |
| LM.Cannabis_x_Herb.THC_x_Daily.Interpol.: LM.Cannabis_x_Resin.THC_x_Daily.Interpol. | 0.18 (0.13, 0.23) | 1.02E-10 | *p*-Value | <2.2E-16 |
| LM.Cannabis_x_Herb.THC: LM.Cannabis_x_Resin.THC_x_Daily.Interpol. | −12.65 (−19.54, −5.76) | 0.000527 | | |

**Table S15**. Inverse Probability Weighted Panel Regression Models - Trisomy 18

| **Parameters** | | | **Model Parameters** | |
|---|---|---|---|---|
| **Term** | **Estimate (C.I.)** | ***p*-Value** | **Parameter** | **Value** |
| | | | | |
| ***Additive*** | | | | |
| *(Rate ~ Tobacco + Alcohol + LM.Cannabis_x_Resin.THC + Daily.Interpol. + LM.Cannabis_x_Herb.THC_x_Daily.Interpol. + Herb + Amphetamines + Cocaine + Income)* | | | | |
| LM.Cannabis_x_Resin.THC | 3.15 (2.3, 4) | 4.94E-11 | Adj.R.Squared | 0.4642 |
| Daily.Interpol. | 9.61 (1.3, 17.92) | 0.0253 | Statistic | 71.1521 |
| Herb | −7.03 (−11.62, −2.44) | 0.0033 | Deg.Freedom | 4,117 |
| Income | 0 (0, 0) | 4.46E-12 | *p*-Value | <2.2E-16 |
| | | | | |
| ***Interactive*** | | | | |
| *(Rate ~ Tobacco * LM.Cannabis_x_Resin.THC_x_Daily.Interpol. * LM.Cannabis_x_Resin.THC + Daily.Interpol. + LM.Cannabis_x_Herb.THC_x_Daily.Interpol. + Alcohol + Amphetamines + Cocaine + Income)* | | | | |
| LM.Cannabis_x_Resin.THC | 2.06 (1.62, 2.5) | 1.65E-15 | Adj.R.Squared | 0.4674 |
| Daily.Interpol. | 16.6 (8.94, 24.26) | 4.26E-05 | Statistic | 67.328 |
| LM.Cannabis_x_Herb.THC_x_Daily.Interpol. | −0.09 (−0.18, 0) | 0.0421 | Deg.Freedom | 4,117 |
| Income | 0 (0, 0) | 5.73E-10 | *p*-Value | <2.2E-16 |
| | | | | |
| ***2 Lags*** | | | | |
| *(Rate ~ Tobacco * Daily.Interpol. + LM.Cannabis_x_Resin.THC + LM.Cannabis_x_Resin.THC_x_Daily.Interpol. + Alcohol + Amphetamines + Cocaine + Income)* | | | | |
| LM.Cannabis_x_Resin.THC | 3.23 (2.79, 3.67) | <2.2E-16 | Adj.R.Squared | 0.3824 |
| Amphetamines | −0.26 (−0.5, −0.02) | 0.0353 | Statistic | 65.9798 |
| Cocaine | 0.39 (0.26, 0.52) | 7.82E-08 | Deg.Freedom | 4,89 |
| Income | 0 (0, 0) | 0.0004 | *p*-Value | <2.2E-16 |

**Table S16**. Inverse Probability Weighted Panel Regression Models

- Trisomy 13

| Parameters | | | Model Parameters | |
|---|---|---|---|---|
| **Term** | **Estimate (C.I.)** | ***p*-Value** | **Parameter** | **Value** |
| | | | | |
| ***Additive*** | | | | |
| *(Rate ~ Tobacco + Alcohol + Daily.Interpol. + LM.Cannabis_x_Resin.THC_x_Daily.Interpol. + LM.Cannabis_x_Herb.THC + LM.Cannabis_x_Herb.THC_x_Daily.Interpol. + Herb + Amphetamines + Cocaine + Income)* | | | | |
| Daily.Interpol. | 74.8 (63.55, 86.05) | < 2e-16 | Adj.R.Squared | 0.2135 |
| Cocaine | −0.93 (−1.15, −0.72) | 1.20E-13 | Statistic | 75.4717 |
| Income | 0 (0, 0) | < 2e-16 | Deg.Freedom | 3,118 |
| | | | *p*-Value | <2.2E-16 |
| | | | | |
| ***Interactive*** | | | | |
| *(Rate ~ Tobacco * Daily.Interpol. + LM.Cannabis_x_Herb.THC * LM.Cannabis_x_Resin.THC_x_Daily.Interpol. + LM.Cannabis_x_Herb.THC_x_Daily.Interpol. + Alcohol + Amphetamines + Cocaine + Income)* | | | | |
| Daily.Interpol. | 226 (124.08, 327.92) | 2.99E-05 | Adj.R.Squared | 0.1241 |
| LM.Cannabis_x_Resin.THC_x_Daily.Interpol. | −1.89 (−2.91, −0.87) | 0.0004 | Statistic | 39.6564 |
| LM.Cannabis_x_Herb.THC_x_Daily.Interpol. | 1.53 (0.66, 2.4) | 0.0008 | Deg.Freedom | 7,114 |
| Cocaine | −0.91 (−1.22, −0.61) | 5.03E-08 | *p*-Value | <2.2E-16 |
| Income | 0 (0, 0) | 5.64E-10 | | |
| Tobacco: Daily.Interpol. | −5.88 (−9.74, −2.02) | 0.0034 | | |
| LM.Cannabis_x_Herb.THC: LM.Cannabis_x_Resin.THC_x_Daily.Interpol. | 7.25 (2.57, 11.93) | 0.0030 | | |
| | | | | |
| ***2 Lags*** | | | | |

| | | | | |
|---|---|---|---|---|
| *(Rate ~ Tobacco * LM.Cannabis_x_Herb.THC + Daily.Interpol. * LM.Cannabis_x_Resin.THC_x_Daily.Interpol. + Alcohol + Daily.Interpol. + Amphetamines + Cocaine + Income)* | | | | |
| Daily.Interpol. | 82.69 (63.64, 101.73) | 3.63E-13 | Adj.R.Squared | −0.0035 |
| Alcohol | −0.3 (−0.39, −0.21) | 2.22E-09 | Statistic | 28.0323 |
| | | | Deg.Freedom | 3,90 |
| | | | *p*-Value | 6.83E-13 |

**Table S17**. Inverse Probability Weighted Panel Regression Models

- Turner syndrome

| Parameters | | | Model Parameters | |
|---|---|---|---|---|
| **Term** | **Estimate (C.I.)** | ***p*-Value** | **Parameter** | **Value** |
| | | | | |
| ***Additive*** | | | | |
| *(Rate ~ Tobacco + Alcohol + Daily.Interpol. + LM.Cannabis_x_Resin.THC_x_Daily.Interpol. + LM.Cannabis_x_Herb.THC_x_Daily.Interpol. + LM.Cannabis_x_Resin.THC + Amphetamines + Cocaine + Income)* | | | | |
| Alcohol | 0.14 (0.06, 0.22) | 0.0007 | Adj.R.Squared | 0.3223 |
| LM.Cannabis_x_Resin.THC | 0.47 (0.15, 0.8) | 0.0055 | Statistic | 10.5331 |
| Amphetamines | −0.26 (−0.45, −0.07) | 0.0098 | Deg.Freedom | 5,116 |
| Cocaine | 0.13 (0.02, 0.25) | 0.0230 | *p*-Value | 5.116.2.35E-08 |
| Income | 0 (0, 0) | 1.46E-06 | | |
| | | | | |
| ***Interactive*** | | | | |
| *(Rate ~ Tobacco * Daily.Interpol. * LM.Cannabis_x_Resin.THC_x_Daily.Interpol. + LM.Cannabis_x_Herb.THC_x_Daily.Interpol. + LM.Cannabis_x_Resin.THC + Alcohol + Amphetamines + Cocaine + Income)* | | | | |
| Tobacco | 0.07 (0.02, 0.12) | 0.0119 | Adj.R.Squared | 0.3333 |
| Daily.Interpol. | 165 (90.13, 239.87) | 3.35E-05 | Statistic | 9.9611 |
| Alcohol | 0.16 (0.07, 0.25) | 0.0006 | Deg.Freedom | 7,114 |
| Amphetamines | −0.42 (−0.63, −0.21) | 0.0002 | *p*-Value | 1.16E-09 |
| Cocaine | 0.31 (0.02, 0.6) | 0.036272 | | |
| Income | 0 (0, 0) | 2.52E-05 | | |
| Tobacco: Daily.Interpol. | −7.09 (−10.5, −3.68) | 8.76E-05 | | |
| | | | | |
| ***2 Lags*** | | | | |

| | | | | |
|---|---|---|---|---|
| *(Rate ~ Tobacco * Daily.Interpol. + LM.Cannabis_x_Resin.THC_x_Daily.Interpol. + LM.Cannabis_x_Resin.THC * LM.Cannabis_x_Herb.THC_x_Daily.Interpol. + Alcohol + Amphetamines + Cocaine + Income)* | | | | |
| LM.Cannabis_x_Resin.THC_x_Daily.Interpol. | −0.32 (−0.49, −0.14) | 0.0005 | Adj.R.Squared | 0.1893 |
| Alcohol | 0.13 (0.04, 0.23) | 0.0065 | Statistic | 5.9699 |
| Amphetamines | −0.35 (−0.61, −0.1) | 0.0083 | Deg.Freedom | 6,87 |
| Cocaine | 0.89 (0.47, 1.31) | 7.83E-05 | *p*-Value | 2.93E-05 |
| Tobacco: Daily.Interpol. | −2.14 (−3.32, −0.96) | 0.0006 | | |
| LM.Cannabis_x_Resin.THC: LM.Cannabis_x_Herb.THC_x_Daily.Interpol. | 8.82 (5.3, 12.34) | 4.16E-06 | | |

**Table S18**. Inverse Probability Weighted Panel Regression Models

- Klinefelter syndrome

| Parameters | | | Model Parameters | |
|---|---|---|---|---|
| **Term** | **Estimate (C.I.)** | ***p*-Value** | **Parameter** | **Value** |
| | | | | |
| ***Additive*** | | | | |
| *(Rate ~ Tobacco + Alcohol + Daily.Interpol. + LM.Cannabis_x_Herb.THC + LM.Cannabis_x_Herb.THC_x_Daily.Interpol. + LM.Cannabis + Amphetamines + Cocaine + Income)* | | | | |
| Alcohol | −0.06 (−0.11, −0.02) | 0.00667 | Adj.R.Squared | 0.1221 |
| Daily.Interpol. | 14.09 (0.33, 27.85) | 0.04712 | Statistic | 59.0404 |
| LM.Cannabis_x_Herb.THC | 8.27 (5.85, 10.69) | 8.40E-10 | Deg.Freedom | 6,115 |
| LM.Cannabis_x_Herb.THC_x_Daily.Interpol. | −0.06 (−0.12, −0.01) | 0.02155 | *p*-Value | <2.2E–16 |
| Amphetamines | −0.14 (−0.27, −0.01) | 0.03372 | | |
| | | | | |
| ***Interactive*** | | | | |
| *(Rate ~ Tobacco * LM.Cannabis_x_Herb.THC + Daily.Interpol. + LM.Cannabis + Alcohol + LM.Cannabis_x_Herb.THC_x_Daily.Interpol. + Amphetamines + Cocaine + Income)* | | | | |
| LM.Cannabis_x_Herb.THC | 24.9 (15.47, 34.32) | 9.55E-07 | Adj.R.Squared | 0.1412 |
| Daily.Interpol. | 9.27 (2.23, 16.31) | 0.01114 | Statistic | 73.6793 |
| LM.Cannabis_x_Herb.THC_x_Daily.Interpol. | −0.07 (−0.13, −0.02) | 0.00682 | Deg.Freedom | 5,116 |
| Amphetamines | −0.19 (−0.31, −0.08) | 0.00145 | *p*-Value | <2.2E-16 |
| Tobacco: LM.Cannabis_x_Herb.THC | −0.72 (−1.15, −0.28) | 0.00149 | | |
| | | | | |
| ***2 Lags*** | | | | |
| *(Rate ~ Tobacco * Daily.Interpol. + LM.Cannabis_x_Herb.THC + LM.Cannabis + LM.Cannabis_x_Herb.THC_x_Daily.Interpol. + Alcohol + Daily.Interpol. + Amphetamines + Cocaine + Income)* | | | | |

| | | | | |
|---|---|---|---|---|
| Tobacco | −0.03 (−0.06, 0) | 0.02575 | Adj.R.Squared | 0.1479 |
| LM.Cannabis_x_Herb.THC | 17.5 (14.32, 20.68) | <2.2E-16 | Statistic | 80.2694 |
| LM.Cannabis | −19.3 (−27.22, −11.38) | 6.97E-06 | Deg.Freedom | 7,86 |
| LM.Cannabis_x_Herb.THC_x_Daily.Interpol. | −0.06 (−0.12, 0) | 0.04447 | *p*-Value | <2.2E-16 |
| Amphetamines | −0.2 (−0.33, −0.06) | 0.00451 | | |
| Cocaine | 0.66 (0.46, 0.85) | 5.52E-09 | | |
| Income | 0 (0, 0) | 8.14E-06 | | |

**Table S19**. Geospatial Models - Turner syndrome

| **Parameter Values** | | | **Model Parameters** | | |
|---|---|---|---|---|---|
| **Parameter** | **Estimate (C.I.)** | ***p*-Value** | **Parameter** | **Value** | **Significance** |
| | | | | | |
| ***Additive*** | | | | | |
| *Rate ~ Tobacco + Alcohol + Daily.Interpol. + LM.Cannabis_x_Resin.THC_x_Daily.Interpol. + LM.Cannabis_x_Herb.THC_x_Daily.Interpol. + LM.Cannabis_x_Resin.THC + Amphetamines + Cocaine + Income* | | | | | |
| Alcohol | 0.16 (0.1, 0.22) | 1.26E-07 | rho | 0.2659 | 0.149 |
| LM.Cannabis_x_Resin.THC_x_Daily.Interpol. | 2.15 (0.67, 3.63) | 0.0044 | lambda | −0.3224 | 0.0369 |
| LM.Cannabis_x_Herb.THC_x_Daily.Interpol. | −4.77 (−8.69, −0.85) | 0.0170 | | | |
| Amphetamines | −0.42 (−0.57, −0.28) | 6.13E-09 | | | |
| Cocaine | 0.48 (0.24, 0.72) | 6.88E-05 | | | |
| Income | 0 (0, 0) | 3.79E-07 | | | |
| | | | | | |
| ***Interactive*** | | | | | |
| *Rate ~ Tobacco + Daily.Interpol. * LM.Cannabis_x_Resin.THC_x_Daily.Interpol. + LM.Cannabis_x_Resin.THC + Alcohol + Amphetamines + Cocaine + Income* | | | | | |
| Tobacco | −0.08 (−0.11, −0.05) | 2.11E-09 | rho | 0.4996 | 1.47E-06 |
| LM.Cannabis_x_Resin.THC_x_Daily.Interpol. | 4.26 (2.58, 5.94) | 7.19E-07 | lambda | −0.50739 | 1.53E-07 |
| Alcohol | 0.2 (0.14, 0.27) | 1.71E-09 | | | |
| Amphetamines | −0.26 (−0.4, −0.13) | 0.0001 | | | |
| Cocaine | 0.43 (0.28, 0.59) | 4.12E-08 | | | |
| Daily.Interpol.: LM.Cannabis_x_Resin.THC_x_Daily.Interpol. | −109.18 (−156.95, −61.41) | 7.48E-06 | | | |
| | | | | | |
| ***2 Lags*** | | | | | |

| | | | | | |
|---|---|---|---|---|---|
| *Rate ~ Tobacco * Daily.Interpol. + LM.Cannabis_x_Resin.THC_x_Daily.Interpol. + LM.Cannabis_x_Resin.THC * LM.Cannabis_x_Herb.THC_x_Daily.Interpol. + Alcohol + Amphetamines + Cocaine + Income* | | | | | |
| Daily.Interpol. | 127 (44.09, 209.91) | 0.0027 | rho | 0.3085 | 0.0385 |
| Alcohol | 0.14 (0.08, 0.21) | 2.80E-05 | lambda | −0.3886 | 0.00229 |
| Amphetamines | −0.28 (−0.43, −0.13) | 0.0002 | | | |
| Income | 0 (0, 0) | 8.16E-08 | | | |
| Tobacco: Daily.Interpol. | −3.77 (−6.95, −0.59) | 0.0199 | | | |

**Table S20**. Geospatial Models - Klinefelter syndrome

| Parameter Values | | | Model Parameters | | |
|---|---|---|---|---|---|
| **Parameter** | **Estimate (C.I.)** | ***p*-Value** | **Parameter** | **Value** | **Significance** |
| | | | | | |
| ***Additive*** | | | | | |
| *Rate ~ Tobacco + Alcohol + Daily.Interpol. + LM.Cannabis_x_Resin.THC_x_Daily.Interpol. + LM.Cannabis_x_Herb.THC + LM.Cannabis_x_Herb.THC_x_Daily.Interpol. + Herb + Amphetamines + Cocaine + Income* | | | | | |
| LM.Cannabis_x_Herb.THC | 11.9 (7.57, 16.23) | 7.27E-08 | rho | 0.5071 | 4.02E-05 |
| Income | 0 (0, 0) | 1.91E-10 | lambda | -0.5445 | 7.53E-06 |
| | | | | | |
| ***Interactive*** | | | | | |
| *Rate ~ Tobacco * LM.Cannabis_x_Herb.THC + Daily.Interpol. + LM.Cannabis + Alcohol + LM.Cannabis_x_Herb.THC_x_Daily.Interpol. + Amphetamines + Cocaine + Income* | | | | | |
| Amphetamines | -0.23 (-0.34, -0.12) | 3.41E-05 | Least Squares | | |
| Cocaine | 0.31 (0.2, 0.43) | 1.27E-07 | S.D. | 0.4299 | |
| | | | | | |
| ***1 Lag*** | | | | | |
| *Rate ~ Tobacco + Daily.Interpol. + LM.Cannabis_x_Herb.THC + LM.Cannabis + LM.Cannabis_x_Herb.THC_x_Daily.Interpol. + Alcohol + Daily.Interpol. + Amphetamines + Cocaine + Income* | | | | | |
| Amphetamines | -0.21 (-0.34, -0.09) | 0.0007 | Least Squares | | |
| Cocaine | 0.31 (0.18, 0.44) | 2.38E-06 | S.D. | 0.4299 | |

**Table S21**. E-Values from Panel Models

| Anomaly | Term | *p*-Value | E-Value Estimate | Lower Bound E-Value |
|---|---|---|---|---|
| | | | | |
| Chromosomes | ***Additive*** | | | |
| | Daily.Interpol. | 3.61E-05 | 2.46E+24 | 2.67E+13 |
| | LM.Cannabis_x_Resin.THC | 1.36E-07 | 18.49 | 8.15 |
| | ***Interactive*** | | | |
| | Daily.Interpol. | 2.22E-09 | Infinity | Infinity |
| | LM.Cannabis_x_Resin.THC | 0.0077 | 74.39 | 4.94 |
| | LM.Cannabis_x_Herb.THC_x_Daily.Interpol. | 0.0198 | 18.43 | 2.31 |
| | ***1 Lag*** | | | |
| | Daily.Interpol. | 2.29E-10 | 2.24E+18 | 2.18E+13 |
| | LM.Cannabis_x_Resin.THC | <2.2E-16 | 1.84E+03 | 510.05 |
| | LM.Cannabis_x_Herb.THC_x_Daily.Interpol. | 0.0002 | 8.08 | 3.58 |
| | ***2 Lags*** | | | |
| | Daily.Interpol. | <2.2E-16 | 1.81E+31 | 2.97E+25 |
| | LM.Cannabis_x_Resin.THC | 5.57E-14 | 2.26E+06 | 1.07E+05 |
| Genetic | ***Additive*** | | | |
| | LM.Cannabis_x_Herb.THC | 9.55E-07 | 2.54E+22 | 1.13E+14 |
| | Daily.Interpol. | 0.0111 | 3.38E+08 | 194.54 |
| | ***Interactive*** | | | |
| | Daily.Interpol. | 4.38E-14 | Infinity | Infinity |
| | LM.Cannabis_x_Herb.THC | 0.0076 | 4.13E+05 | 61.12 |
| | LM.Cannabis_x_Herb.THC_x_Daily.Interpol. | 7.63E-06 | 27.40 | 8.77 |
| | ***1 Lag*** | | | |

| | | | | |
|---|---|---|---|---|
| | LM.Cannabis_x_Resin.THC_x_Daily.Interpol. | 0.0047 | 7.89 | 2.56 |
| | Tobacco: LM.Cannabis_x_Herb.THC | 0.0023 | 3.40 | 1.91 |
| | ***2 Lags*** | | | |
| | Daily.Interpol. | 0.0142 | 6.49E+115 | 2.92E+25 |
| | LM.Cannabis_x_Herb.THC | 0.0178 | 2.24E+05 | 17.73 |
| Trisomy 21 | ***Additive*** | | | |
| | LM.Cannabis_x_Herb.THC | <2.2E-16 | 3.59E+10 | 5.29E+08 |
| | ***Interactive*** | | | |
| | LM.Cannabis_x_Herb.THC | 0.000628 | 2.82E+45 | 2.23E+20 |
| | LM.Cannabis_x_Resin.THC_x_Daily.Interpol. | 2.06E-06 | 104.48 | 21.84 |
| | LM.Cannabis_x_Herb.THC_x_Daily.Interpol.: LM.Cannabis_x_Herb.THC | 5.56E-05 | 3.98E+26 | 2.11E+14 |
| | ***2 Lags*** | | | |
| | LM.Cannabis_x_Herb.THC: LM.Cannabis_x_Resin.THC_x_Daily.Interpol. | 8.48E-10 | 86.25 | 29.18 |
| | LM.Cannabis_x_Herb.THC_x_Daily.Interpol.: LM.Cannabis_x_Resin.THC_x_Daily.Interpol. | 1.02E-10 | 2.18 | 1.90 |
| Trisomy 18 | ***Additive*** | | | |
| | LM.Cannabis_x_Resin.THC | 4.94E-11 | 927.29 | 176.39 |
| | Daily.Interpol. | 0.0253 | 2.78E+08 | 25.51 |
| | ***Interactive*** | | | |
| | LM.Cannabis_x_Resin.THC | 1.65E-15 | 23.77 | 13.76 |
| | Daily.Interpol. | 4.26E-05 | 1.19E+09 | 1.06E+05 |
| | ***2 Lags*** | | | |
| | LM.Cannabis_x_Resin.THC | <2.2E-16 | 101.56 | 59.31 |
| Trisomy 13 | ***Additive*** | | | |
| | Daily.Interpol. | < 2e-16 | 2.20E+40 | 2.16E+34 |
| | ***Interactive*** | | | |
| | Daily.Interpol. | 2.99E-05 | 2.17E+129 | 2.02E+71 |

| | | | | |
|---|---|---|---|---|
| | LM.Cannabis_x_Herb.THC_x_Daily.Interpol. | 0.0008 | 14.37 | 4.18 |
| | LM.Cannabis_x_Herb.THC: LM.Cannabis_x_Resin.THC_x_Daily.Interpol. | 0.0030 | 2.76E+04 | 58.55 |
| | ***2 Lags*** | | | |
| | Daily.Interpol. | 3.63E-13 | 5.48E+32 | 1.91E+25 |
| Turners Syn. | ***Additive*** | | | |
| | LM.Cannabis_x_Resin.THC | 0.0055 | 3.14 | 1.79 |
| | ***Interactive*** | | | |
| | Daily.Interpol. | 3.35E-05 | 1.15E+99 | 2.21E+54 |
| | ***2 Lags*** | | | |
| | LM.Cannabis_x_Resin.THC: LM.Cannabis_x_Herb.THC_x_Daily.Interpol. | 4.16E-06 | 1.18E+05 | 1.49E+03 |
| Klinefelters | ***Additive*** | | | |
| | Daily.Interpol. | 0.04712 | 4.11E+12 | 3.51 |
| | LM.Cannabis_x_Herb.THC | 8.40E-10 | 3.36E+07 | 2.59E+05 |
| | ***Interactive*** | | | |
| | LM.Cannabis_x_Herb.THC | 9.55E-07 | 2.53E+22 | 1.13E+14 |
| | Daily.Interpol. | 0.01114 | 3.38E+08 | 194.54 |
| | ***2 Lags*** | | | |
| | LM.Cannabis_x_Herb.THC | <2.2E-16 | 9.90E+18 | 4.11E+15 |

**Table S22**. E-Values from Geospatial Models

| Anomaly | Term | *p*-Value | E-Value Estimate | Lower Bound E-Value |
|---|---|---|---|---|
| | | | | |
| Chromosomes | ***Additive*** | | | |
| | Daily.Interpol. | 3.61E-05 | 2.46E+24 | 2.67E+13 |
| | LM.Cannabis_x_Resin.THC | 1.36E-07 | 18.49 | 8.15 |
| | ***Interactive*** | | | |
| | Daily.Interpol. | 2.22E-09 | Infinity | Infinity |
| | LM.Cannabis_x_Resin.THC | 0.0077 | 74.39 | 4.94 |
| | LM.Cannabis_x_Herb.THC_x_Daily.Interpol. | 0.0198 | 18.43 | 2.31 |
| | ***1 Lag*** | | | |
| | Daily.Interpol. | 2.29E-10 | 2.24E+18 | 2.18E+13 |
| | LM.Cannabis_x_Resin.THC | <2.2E-16 | 1.84E+03 | 510.05 |
| | LM.Cannabis_x_Herb.THC_x_Daily.Interpol. | 0.0002 | 8.08 | 3.58 |
| | ***2 Lags*** | | | |
| | Daily.Interpol. | <2.2E-16 | 1.81E+31 | 2.97E+25 |
| | LM.Cannabis_x_Resin.THC | 5.57E-14 | 2.26E+06 | 1.07E+05 |
| Genetic | ***Additive*** | | | |
| | LM.Cannabis_x_Herb.THC | 9.55E-07 | 2.54E+22 | 1.13E+14 |
| | Daily.Interpol. | 0.0111 | 3.38E+08 | 194.54 |
| | ***Interactive*** | | | |
| | Daily.Interpol. | 4.38E-14 | Infinity | Infinity |
| | LM.Cannabis_x_Herb.THC | 0.0076 | 4.13E+05 | 61.12 |
| | LM.Cannabis_x_Herb.THC_x_Daily.Interpol. | 7.63E-06 | 27.40 | 8.77 |
| | ***1 Lag*** | | | |

| | | | | |
|---|---|---|---|---|
| | LM.Cannabis_x_Resin.THC_x_Daily.Interpol. | 0.0047 | 7.89 | 2.56 |
| | Tobacco: LM.Cannabis_x_Herb.THC | 0.0023 | 3.40 | 1.91 |
| | ***2 Lags*** | | | |
| | Daily.Interpol. | 0.0142 | 6.49E+115 | 2.92E+25 |
| | LM.Cannabis_x_Herb.THC | 0.0178 | 2.24E+05 | 17.73 |
| Trisomy 21 | ***Additive*** | | | |
| | LM.Cannabis_x_Herb.THC | <2.2E-16 | 3.59E+10 | 5.29E+08 |
| | ***Interactive*** | | | |
| | LM.Cannabis_x_Herb.THC | 0.000628 | 2.82E+45 | 2.23E+20 |
| | LM.Cannabis_x_Resin.THC_x_Daily.Interpol. | 2.06E-06 | 104.48 | 21.84 |
| | LM.Cannabis_x_Herb.THC_x_Daily.Interpol.: LM.Cannabis_x_Herb.THC | 5.56E-05 | 3.98E+26 | 2.11E+14 |
| | ***2 Lags*** | | | |
| | LM.Cannabis_x_Herb.THC: LM.Cannabis_x_Resin.THC_x_Daily.Interpol. | 8.48E-10 | 86.25 | 29.18 |
| | LM.Cannabis_x_Herb.THC_x_Daily.Interpol.: LM.Cannabis_x_Resin.THC_x_Daily.Interpol. | 1.02E-10 | 2.18 | 1.90 |
| Trisomy 18 | ***Additive*** | | | |
| | LM.Cannabis_x_Resin.THC | 4.94E-11 | 927.29 | 176.39 |
| | Daily.Interpol. | 0.0253 | 2.78E+08 | 25.51 |
| | ***Interactive*** | | | |
| | LM.Cannabis_x_Resin.THC | 1.65E-15 | 23.77 | 13.76 |
| | Daily.Interpol. | 4.26E-05 | 1.19E+09 | 1.06E+05 |
| | ***2 Lags*** | | | |
| | LM.Cannabis_x_Resin.THC | <2.2E-16 | 101.56 | 59.31 |
| Trisomy 13 | ***Additive*** | | | |
| | Daily.Interpol. | < 2e-16 | 2.20E+40 | 2.16E+34 |
| | ***Interactive*** | | | |
| | Daily.Interpol. | 2.99E-05 | 2.17E+129 | 2.02E+71 |

| | | | | |
|---|---|---|---|---|
| | LM.Cannabis_x_Herb.THC_x_Daily.Interpol. | 0.0008 | 14.37 | 4.18 |
| | LM.Cannabis_x_Herb.THC: LM.Cannabis_x_Resin.THC_x_Daily.Interpol. | 0.0030 | 2.76E+04 | 58.55 |
| | ***2 Lags*** | | | |
| | Daily.Interpol. | 3.63E-13 | 5.48E+32 | 1.91E+25 |
| Turners Syn. | ***Additive*** | | | |
| | LM.Cannabis_x_Resin.THC | 0.0055 | 3.14 | 1.79 |
| | ***Interactive*** | | | |
| | Daily.Interpol. | 3.35E-05 | 1.15E+99 | 2.21E+54 |
| | ***2 Lags*** | | | |
| | LM.Cannabis_x_Resin.THC: LM.Cannabis_x_Herb.THC_x_Daily.Interpol. | 4.16E-06 | 1.18E+05 | 1.49E+03 |
| Klinefelters | ***Additive*** | | | |
| | Daily.Interpol. | 0.04712 | 4.11E+12 | 3.51 |
| | LM.Cannabis_x_Herb.THC | 8.40E-10 | 3.36E+07 | 2.59E+05 |
| | ***Interactive*** | | | |
| | LM.Cannabis_x_Herb.THC | 9.55E-07 | 2.53E+22 | 1.13E+14 |
| | Daily.Interpol. | 0.01114 | 3.38E+08 | 194.54 |
| | ***2 Lags*** | | | |
| | LM.Cannabis_x_Herb.THC | <2.2E-16 | 9.90E+18 | 4.11E+15 |

**Table S23**. E-Values Ordered by Anomaly

| Anomaly | Regression | Model Type | Term | Group | *p*-Value | E-Value Estimate | Lower Bound E-Value |
|---|---|---|---|---|---|---|---|
| | | | | | | | |
| Chromosomes | Panel | Interactive | Daily.Interpol. | Daily | 2.22E-09 | Infinity | Infinity |
| Chromosomes | Spatial | Interactive | Daily.Interpol. | Daily | 2.22E-09 | Infinity | Infinity |
| Chromosomes | Panel | 2 Lags | Daily.Interpol. | Daily | <2.2E-16 | 1.81E+31 | 2.97E+25 |
| Chromosomes | Spatial | 2 Lags | Daily.Interpol. | Daily | <2.2E-16 | 1.81E+31 | 2.97E+25 |
| Chromosomes | Panel | Additive | Daily.Interpol. | Daily | 3.61E-05 | 2.46E+24 | 2.67E+13 |
| Chromosomes | Spatial | Additive | Daily.Interpol. | Daily | 3.61E-05 | 2.46E+24 | 2.67E+13 |
| Chromosomes | Panel | 1 Lag | Daily.Interpol. | Daily | 2.29E-10 | 2.24E+18 | 2.18E+13 |
| Chromosomes | Spatial | 1 Lag | Daily.Interpol. | Daily | 2.29E-10 | 2.24E+18 | 2.18E+13 |
| Chromosomes | Panel | 2 Lags | LM.Cannabis_x_Resin.THC | Herb | 5.57E-14 | 2.26E+06 | 1.07E+05 |
| Chromosomes | Spatial | 2 Lags | LM.Cannabis_x_Resin.THC | Herb | 5.57E-14 | 2.26E+06 | 1.07E+05 |
| Chromosomes | Panel | 1 Lag | LM.Cannabis_x_Resin.THC | Daily | <2.2E-16 | 1.84E+03 | 510.05 |
| Chromosomes | Spatial | 1 Lag | LM.Cannabis_x_Resin.THC | Resin | <2.2E-16 | 1.84E+03 | 510.05 |
| Chromosomes | Panel | Additive | LM.Cannabis_x_Resin.THC | Resin | 1.36E-07 | 18.49 | 8.15 |
| Chromosomes | Spatial | Additive | LM.Cannabis_x_Resin.THC | Resin | 1.36E-07 | 18.49 | 8.15 |
| Chromosomes | Panel | Interactive | LM.Cannabis_x_Resin.THC | Resin | 0.0077 | 74.39 | 4.94 |
| Chromosomes | Spatial | Interactive | LM.Cannabis_x_Resin.THC | Resin | 0.0077 | 74.39 | 4.94 |
| Chromosomes | Panel | 1 Lag | LM.Cannabis_x_Herb.THC_x_Daily.Interpol. | Herb | 0.0002 | 8.08 | 3.58 |
| Chromosomes | Spatial | 1 Lag | LM.Cannabis_x_Herb.THC_x_Daily.Interpol. | Herb | 0.0002 | 8.08 | 3.58 |
| Chromosomes | Panel | Interactive | LM.Cannabis_x_Herb.THC_x_Daily.Interpol. | Daily | 0.0198 | 18.43 | 2.31 |

| | | | | | | | |
|---|---|---|---|---|---|---|---|
| Chromosomes | Spatial | Interactive | LM.Cannabis_x_Herb.THC_x_Daily.Interpol. | Daily | 0.0198 | 18.43 | 2.31 |
| Genetic | Panel | Interactive | Daily.Interpol. | Daily | 4.38E-14 | Infinity | Infinity |
| Genetic | Spatial | Interactive | Daily.Interpol. | Daily | 4.38E-14 | Infinity | Infinity |
| Genetic | Panel | 2 Lags | Daily.Interpol. | Daily | 0.0142 | 6.49E+115 | 2.92E+25 |
| Genetic | Spatial | 2 Lags | Daily.Interpol. | Daily | 0.0142 | 6.49E+115 | 2.92E+25 |
| Genetic | Panel | Additive | LM.Cannabis_x_Herb.THC | Herb | 9.55E-07 | 2.54E+22 | 1.13E+14 |
| Genetic | Spatial | Additive | LM.Cannabis_x_Herb.THC | Herb | 9.55E-07 | 2.54E+22 | 1.13E+14 |
| Genetic | Panel | Additive | Daily.Interpol. | Daily | 0.0111 | 3.38E+08 | 194.54 |
| Genetic | Spatial | Additive | Daily.Interpol. | Daily | 0.0111 | 3.38E+08 | 194.54 |
| Genetic | Panel | Interactive | LM.Cannabis_x_Herb.THC | Resin | 0.0076 | 4.13E+05 | 61.12 |
| Genetic | Spatial | Interactive | LM.Cannabis_x_Herb.THC | Resin | 0.0076 | 4.13E+05 | 61.12 |
| Genetic | Panel | 2 Lags | LM.Cannabis_x_Herb.THC | Herb | 0.0178 | 2.24E+05 | 17.73 |
| Genetic | Spatial | 2 Lags | LM.Cannabis_x_Herb.THC | Herb | 0.0178 | 2.24E+05 | 17.73 |
| Genetic | Panel | Interactive | LM.Cannabis_x_Herb.THC_x_Daily.Interpol. | Daily | 7.63E-06 | 27.40 | 8.77 |
| Genetic | Spatial | Interactive | LM.Cannabis_x_Herb.THC_x_Daily.Interpol. | Daily | 7.63E-06 | 27.40 | 8.77 |
| Genetic | Panel | 1 Lag | LM.Cannabis_x_Resin.THC_x_Daily.Interpol. | Daily | 0.0047 | 7.89 | 2.56 |
| Genetic | Spatial | 1 Lag | LM.Cannabis_x_Resin.THC_x_Daily.Interpol. | Daily | 0.0047 | 7.89 | 2.56 |
| Genetic | Panel | 1 Lag | Tobacco: LM.Cannabis_x_Herb.THC | Herb | 0.0023 | 3.40 | 1.91 |
| Genetic | Spatial | 1 Lag | Tobacco: LM.Cannabis_x_Herb.THC | Herb | 0.0023 | 3.40 | 1.91 |
| Klinefelters | Panel | 2 Lags | LM.Cannabis_x_Herb.THC | Herb | <2.2E-16 | 9.90E+18 | 4.11E+15 |
| Klinefelters | Spatial | 2 Lags | LM.Cannabis_x_Herb.THC | Herb | <2.2E-16 | 9.90E+18 | 4.11E+15 |
| Klinefelters | Panel | Interactive | LM.Cannabis_x_Herb.THC | Herb | 9.55E-07 | 2.53E+22 | 1.13E+14 |
| Klinefelters | Spatial | Interactive | LM.Cannabis_x_Herb.THC | Herb | 9.55E-07 | 2.53E+22 | 1.13E+14 |
| Klinefelters | Panel | Additive | LM.Cannabis_x_Herb.THC | Herb | 8.40E-10 | 3.36E+07 | 2.59E+05 |

| | | | | | | | |
|---|---|---|---|---|---|---|---|
| Klinefelters | Spatial | Additive | LM.Cannabis_x_Herb.THC | Herb | 8.40E-10 | 3.36E+07 | 2.59E+05 |
| Klinefelters | Panel | Interactive | Daily.Interpol. | Daily | 0.01114 | 3.38E+08 | 194.54 |
| Klinefelters | Spatial | Interactive | Daily.Interpol. | Daily | 0.01114 | 3.38E+08 | 194.54 |
| Klinefelters | Panel | Additive | Daily.Interpol. | Daily | 0.04712 | 4.11E+12 | 3.51 |
| Klinefelters | Spatial | Additive | Daily.Interpol. | Daily | 0.04712 | 4.11E+12 | 3.51 |
| Trisomy 13 | Panel | Interactive | Daily.Interpol. | Daily | 2.99E-05 | 2.17E+129 | 2.02E+71 |
| Trisomy 13 | Spatial | Interactive | Daily.Interpol. | Daily | 2.99E-05 | 2.17E+129 | 2.02E+71 |
| Trisomy 13 | Panel | Additive | Daily.Interpol. | Daily | < 2e-16 | 2.20E+40 | 2.16E+34 |
| Trisomy 13 | Spatial | Additive | Daily.Interpol. | Daily | < 2e-16 | 2.20E+40 | 2.16E+34 |
| Trisomy 13 | Panel | 2 Lags | Daily.Interpol. | Daily | 3.63E-13 | 5.48E+32 | 1.91E+25 |
| Trisomy 13 | Spatial | 2 Lags | Daily.Interpol. | Daily | 3.63E-13 | 5.48E+32 | 1.91E+25 |
| Trisomy 13 | Panel | Interactive | LM.Cannabis_x_Herb.THC: LM.Cannabis_x_Resin.THC_x_Daily.Interpol. | Daily | 0.0030 | 2.76E+04 | 58.55 |
| Trisomy 13 | Spatial | Interactive | LM.Cannabis_x_Herb.THC: LM.Cannabis_x_Resin.THC_x_Daily.Interpol. | Daily | 0.0030 | 2.76E+04 | 58.55 |
| Trisomy 13 | Panel | Interactive | LM.Cannabis_x_Herb.THC_x_Daily.Interpol. | Herb | 0.0008 | 14.37 | 4.18 |
| Trisomy 13 | Spatial | Interactive | LM.Cannabis_x_Herb.THC_x_Daily.Interpol. | Herb | 0.0008 | 14.37 | 4.18 |
| Trisomy 18 | Panel | Interactive | Daily.Interpol. | Daily | 4.26E-05 | 1.19E+09 | 1.06E+05 |
| Trisomy 18 | Spatial | Interactive | Daily.Interpol. | Daily | 4.26E-05 | 1.19E+09 | 1.06E+05 |
| Trisomy 18 | Panel | Additive | LM.Cannabis_x_Resin.THC | Resin | 4.94E-11 | 927.29 | 176.39 |
| Trisomy 18 | Spatial | Additive | LM.Cannabis_x_Resin.THC | Resin | 4.94E-11 | 927.29 | 176.39 |
| Trisomy 18 | Panel | 2 Lags | LM.Cannabis_x_Resin.THC | Resin | <2.2E-16 | 101.56 | 59.31 |
| Trisomy 18 | Spatial | 2 Lags | LM.Cannabis_x_Resin.THC | Resin | <2.2E-16 | 101.56 | 59.31 |
| Trisomy 18 | Panel | Additive | Daily.Interpol. | Daily | 0.0253 | 2.78E+08 | 25.51 |
| Trisomy 18 | Spatial | Additive | Daily.Interpol. | Daily | 0.0253 | 2.78E+08 | 25.51 |

| | | | | | | | |
|---|---|---|---|---|---|---|---|
| Trisomy 18 | Panel | Interactive | LM.Cannabis_x_Resin.THC | Herb | 1.65E-15 | 23.77 | 13.76 |
| Trisomy 18 | Spatial | Interactive | LM.Cannabis_x_Resin.THC | Herb | 1.65E-15 | 23.77 | 13.76 |
| Trisomy 21 | Panel | Interactive | LM.Cannabis_x_Herb.THC | Herb | 0.000628 | 2.82E+45 | 2.23E+20 |
| Trisomy 21 | Spatial | Interactive | LM.Cannabis_x_Herb.THC | Herb | 0.000628 | 2.82E+45 | 2.23E+20 |
| Trisomy 21 | Panel | Interactive | LM.Cannabis_x_Herb.THC_x_Daily.Interpol.: LM.Cannabis_x_Herb.THC | Daily | 5.56E-05 | 3.98E+26 | 2.11E+14 |
| Trisomy 21 | Spatial | Interactive | LM.Cannabis_x_Herb.THC_x_Daily.Interpol.: LM.Cannabis_x_Herb.THC | Daily | 5.56E-05 | 3.98E+26 | 2.11E+14 |
| Trisomy 21 | Panel | Additive | LM.Cannabis_x_Herb.THC | Herb | <2.2E-16 | 3.59E+10 | 5.29E+08 |
| Trisomy 21 | Spatial | Additive | LM.Cannabis_x_Herb.THC | Herb | <2.2E-16 | 3.59E+10 | 5.29E+08 |
| Trisomy 21 | Panel | 2 Lags | LM.Cannabis_x_Herb.THC: LM.Cannabis_x_Resin.THC_x_Daily.Interpol. | Daily | 8.48E-10 | 86.25 | 29.18 |
| Trisomy 21 | Spatial | 2 Lags | LM.Cannabis_x_Herb.THC: LM.Cannabis_x_Resin.THC_x_Daily.Interpol. | Daily | 8.48E-10 | 86.25 | 29.18 |
| Trisomy 21 | Panel | Interactive | LM.Cannabis_x_Resin.THC_x_Daily.Interpol. | Daily | 2.06E-06 | 104.48 | 21.84 |
| Trisomy 21 | Spatial | Interactive | LM.Cannabis_x_Resin.THC_x_Daily.Interpol. | Daily | 2.06E-06 | 104.48 | 21.84 |
| Trisomy 21 | Panel | 2 Lags | LM.Cannabis_x_Herb.THC_x_Daily.Interpol.: LM.Cannabis_x_Resin.THC_x_Daily.Interpol. | Daily | 1.02E-10 | 2.18 | 1.90 |
| Trisomy 21 | Spatial | 2 Lags | LM.Cannabis_x_Herb.THC_x_Daily.Interpol.: LM.Cannabis_x_Resin.THC_x_Daily.Interpol. | Daily | 1.02E-10 | 2.18 | 1.90 |
| Turners | Panel | Interactive | Daily.Interpol. | Daily | 3.35E-05 | 1.15E+99 | 2.21E+54 |

| | | | | | | | |
|---|---|---|---|---|---|---|---|
| Turners | Spatial | Interactive | Daily.Interpol. | Daily | 3.35E-05 | 1.15E+99 | 2.21E+54 |
| Turners | Panel | 2 Lags | LM.Cannabis_x_Resin.THC: LM.Cannabis_x_Herb.THC_x_Daily.Interpol. | Daily | 4.16E-06 | 1.18E+05 | 1.49E+03 |
| Turners | Spatial | 2 Lags | LM.Cannabis_x_Resin.THC: LM.Cannabis_x_Herb.THC_x_Daily.Interpol. | Daily | 4.16E-06 | 1.18E+05 | 1.49E+03 |
| Turners | Panel | Additive | LM.Cannabis_x_Resin.THC | Resin | 0.0055 | 3.14 | 1.79 |
| Turners | Spatial | Additive | LM.Cannabis_x_Resin.THC | Resin | 0.0055 | 3.14 | 1.79 |

**Table S24**. E-Values ordered by group of covariates

| Anomaly | Regression | Model Type | Term | Group | *p*-Value | E-Value Estimate | Lower Bound E-Value |
|---|---|---|---|---|---|---|---|
| | | | | | | | |
| Chromosomes | Panel | Interactive | Daily.Interpol. | Daily | 2.22E-09 | Infinity | Infinity |
| Genetic | Panel | Interactive | Daily.Interpol. | Daily | 4.38E-14 | Infinity | Infinity |
| Chromosomes | Spatial | Interactive | Daily.Interpol. | Daily | 2.22E-09 | Infinity | Infinity |
| Genetic | Spatial | Interactive | Daily.Interpol. | Daily | 4.38E-14 | Infinity | Infinity |
| Trisomy 13 | Panel | Interactive | Daily.Interpol. | Daily | 2.99E-05 | 2.17E+129 | 2.02E+71 |
| Trisomy 13 | Spatial | Interactive | Daily.Interpol. | Daily | 2.99E-05 | 2.17E+129 | 2.02E+71 |
| Turners | Panel | Interactive | Daily.Interpol. | Daily | 3.35E-05 | 1.15E+99 | 2.21E+54 |
| Turners | Spatial | Interactive | Daily.Interpol. | Daily | 3.35E-05 | 1.15E+99 | 2.21E+54 |
| Trisomy 13 | Panel | Additive | Daily.Interpol. | Daily | < 2e-16 | 2.20E+40 | 2.16E+34 |
| Trisomy 13 | Spatial | Additive | Daily.Interpol. | Daily | < 2e-16 | 2.20E+40 | 2.16E+34 |
| Chromosomes | Panel | 2 Lags | Daily.Interpol. | Daily | <2.2E-16 | 1.81E+31 | 2.97E+25 |
| Chromosomes | Spatial | 2 Lags | Daily.Interpol. | Daily | <2.2E-16 | 1.81E+31 | 2.97E+25 |
| Genetic | Panel | 2 Lags | Daily.Interpol. | Daily | 0.0142 | 6.49E+115 | 2.92E+25 |
| Genetic | Spatial | 2 Lags | Daily.Interpol. | Daily | 0.0142 | 6.49E+115 | 2.92E+25 |
| Trisomy 13 | Panel | 2 Lags | Daily.Interpol. | Daily | 3.63E-13 | 5.48E+32 | 1.91E+25 |
| Trisomy 13 | Spatial | 2 Lags | Daily.Interpol. | Daily | 3.63E-13 | 5.48E+32 | 1.91E+25 |
| Trisomy 21 | Panel | Interactive | LM.Cannabis_x_Herb.THC_x_Daily.Interpol.: LM.Cannabis_x_Herb.THC | Daily | 5.56E-05 | 3.98E+26 | 2.11E+14 |
| Trisomy 21 | Spatial | Interactive | LM.Cannabis_x_Herb.THC_x_Daily.Interpol.: LM.Cannabis_x_Herb.THC | Daily | 5.56E-05 | 3.98E+26 | 2.11E+14 |
| Chromosomes | Panel | Additive | Daily.Interpol. | Daily | 3.61E-05 | 2.46E+24 | 2.67E+13 |
| Chromosomes | Spatial | Additive | Daily.Interpol. | Daily | 3.61E-05 | 2.46E+24 | 2.67E+13 |

| | | | | | | | |
|---|---|---|---|---|---|---|---|
| Chromosomes | Panel | 1 Lag | Daily.Interpol. | Daily | 2.29E-10 | 2.24E+18 | 2.18E+13 |
| Chromosomes | Spatial | 1 Lag | Daily.Interpol. | Daily | 2.29E-10 | 2.24E+18 | 2.18E+13 |
| Trisomy 18 | Panel | Interactive | Daily.Interpol. | Daily | 4.26E-05 | 1.19E+09 | 1.06E+05 |
| Trisomy 18 | Spatial | Interactive | Daily.Interpol. | Daily | 4.26E-05 | 1.19E+09 | 1.06E+05 |
| Turners | Panel | 2 Lags | LM.Cannabis_x_Resin.THC: LM.Cannabis_x_Herb.THC_x_Daily.Interpol. | Daily | 4.16E-06 | 1.18E+05 | 1.49E+03 |
| Turners | Spatial | 2 Lags | LM.Cannabis_x_Resin.THC: LM.Cannabis_x_Herb.THC_x_Daily.Interpol. | Daily | 4.16E-06 | 1.18E+05 | 1.49E+03 |
| Chromosomes | Panel | 1 Lag | LM.Cannabis_x_Resin.THC | Daily | <2.2E-16 | 1.84E+03 | 510.05 |
| Genetic | Panel | Additive | Daily.Interpol. | Daily | 0.0111 | 3.38E+08 | 194.54 |
| Klinefelters | Panel | Interactive | Daily.Interpol. | Daily | 0.01114 | 3.38E+08 | 194.54 |
| Genetic | Spatial | Additive | Daily.Interpol. | Daily | 0.0111 | 3.38E+08 | 194.54 |
| Klinefelters | Spatial | Interactive | Daily.Interpol. | Daily | 0.01114 | 3.38E+08 | 194.54 |
| Trisomy 13 | Panel | Interactive | LM.Cannabis_x_Herb.THC: LM.Cannabis_x_Resin.THC_x_Daily.Interpol. | Daily | 0.0030 | 2.76E+04 | 58.55 |
| Trisomy 13 | Spatial | Interactive | LM.Cannabis_x_Herb.THC: LM.Cannabis_x_Resin.THC_x_Daily.Interpol. | Daily | 0.0030 | 2.76E+04 | 58.55 |
| Trisomy 21 | Panel | 2 Lags | LM.Cannabis_x_Herb.THC: LM.Cannabis_x_Resin.THC_x_Daily.Interpol. | Daily | 8.48E-10 | 86.25 | 29.18 |
| Trisomy 21 | Spatial | 2 Lags | LM.Cannabis_x_Herb.THC: LM.Cannabis_x_Resin.THC_x_Daily.Interpol. | Daily | 8.48E-10 | 86.25 | 29.18 |
| Trisomy 18 | Panel | Additive | Daily.Interpol. | Daily | 0.0253 | 2.78E+08 | 25.51 |
| Trisomy 18 | Spatial | Additive | Daily.Interpol. | Daily | 0.0253 | 2.78E+08 | 25.51 |
| Trisomy 21 | Panel | Interactive | LM.Cannabis_x_Resin.THC_x_Daily.Interpol. | Daily | 2.06E-06 | 104.48 | 21.84 |
| Trisomy 21 | Spatial | Interactive | LM.Cannabis_x_Resin.THC_x_Daily.Interpol. | Daily | 2.06E-06 | 104.48 | 21.84 |
| Genetic | Panel | Interactive | LM.Cannabis_x_Herb.THC_x_Daily.Interpol. | Daily | 7.63E-06 | 27.40 | 8.77 |
| Genetic | Spatial | Interactive | LM.Cannabis_x_Herb.THC_x_Daily.Interpol. | Daily | 7.63E-06 | 27.40 | 8.77 |
| Klinefelters | Panel | Additive | Daily.Interpol. | Daily | 0.04712 | 4.11E+12 | 3.51 |
| Klinefelters | Spatial | Additive | Daily.Interpol. | Daily | 0.04712 | 4.11E+12 | 3.51 |

| | | | | | | | |
|---|---|---|---|---|---|---|---|
| Genetic | Panel | 1 Lag | LM.Cannabis_x_Resin.THC_x_Daily.Interpol. | Daily | 0.0047 | 7.89 | 2.56 |
| Genetic | Spatial | 1 Lag | LM.Cannabis_x_Resin.THC_x_Daily.Interpol. | Daily | 0.0047 | 7.89 | 2.56 |
| Chromosomes | Panel | Interactive | LM.Cannabis_x_Herb.THC_x_Daily.Interpol. | Daily | 0.0198 | 18.43 | 2.31 |
| Chromosomes | Spatial | Interactive | LM.Cannabis_x_Herb.THC_x_Daily.Interpol. | Daily | 0.0198 | 18.43 | 2.31 |
| Trisomy 21 | Panel | 2 Lags | LM.Cannabis_x_Herb.THC_x_Daily.Interpol.: LM.Cannabis_x_Resin.THC_x_Daily.Interpol. | Daily | 1.02E-10 | 2.18 | 1.90 |
| Trisomy 21 | Spatial | 2 Lags | LM.Cannabis_x_Herb.THC_x_Daily.Interpol.: LM.Cannabis_x_Resin.THC_x_Daily.Interpol. | Daily | 1.02E-10 | 2.18 | 1.90 |
| Trisomy 21 | Panel | Interactive | LM.Cannabis_x_Herb.THC | Herb | 0.000628 | 2.82E+45 | 2.23E+20 |
| Trisomy 21 | Spatial | Interactive | LM.Cannabis_x_Herb.THC | Herb | 0.000628 | 2.82E+45 | 2.23E+20 |
| Klinefelters | Panel | 2 Lags | LM.Cannabis_x_Herb.THC | Herb | <2.2E-16 | 9.90E+18 | 4.11E+15 |
| Klinefelters | Spatial | 2 Lags | LM.Cannabis_x_Herb.THC | Herb | <2.2E-16 | 9.90E+18 | 4.11E+15 |
| Genetic | Panel | Additive | LM.Cannabis_x_Herb.THC | Herb | 9.55E-07 | 2.54E+22 | 1.13E+14 |
| Klinefelters | Panel | Interactive | LM.Cannabis_x_Herb.THC | Herb | 9.55E-07 | 2.53E+22 | 1.13E+14 |
| Genetic | Spatial | Additive | LM.Cannabis_x_Herb.THC | Herb | 9.55E-07 | 2.54E+22 | 1.13E+14 |
| Klinefelters | Spatial | Interactive | LM.Cannabis_x_Herb.THC | Herb | 9.55E-07 | 2.53E+22 | 1.13E+14 |
| Trisomy 21 | Panel | Additive | LM.Cannabis_x_Herb.THC | Herb | <2.2E-16 | 3.59E+10 | 5.29E+08 |
| Trisomy 21 | Spatial | Additive | LM.Cannabis_x_Herb.THC | Herb | <2.2E-16 | 3.59E+10 | 5.29E+08 |
| Klinefelters | Panel | Additive | LM.Cannabis_x_Herb.THC | Herb | 8.40E-10 | 3.36E+07 | 2.59E+05 |
| Klinefelters | Spatial | Additive | LM.Cannabis_x_Herb.THC | Herb | 8.40E-10 | 3.36E+07 | 2.59E+05 |
| Chromosomes | Panel | 2 Lags | LM.Cannabis_x_Resin.THC | Herb | 5.57E-14 | 2.26E+06 | 1.07E+05 |
| Chromosomes | Spatial | 2 Lags | LM.Cannabis_x_Resin.THC | Herb | 5.57E-14 | 2.26E+06 | 1.07E+05 |
| Genetic | Panel | 2 Lags | LM.Cannabis_x_Herb.THC | Herb | 0.0178 | 2.24E+05 | 17.73 |
| Genetic | Spatial | 2 Lags | LM.Cannabis_x_Herb.THC | Herb | 0.0178 | 2.24E+05 | 17.73 |
| Trisomy 18 | Panel | Interactive | LM.Cannabis_x_Resin.THC | Herb | 1.65E-15 | 23.77 | 13.76 |
| Trisomy 18 | Spatial | Interactive | LM.Cannabis_x_Resin.THC | Herb | 1.65E-15 | 23.77 | 13.76 |
| Trisomy 13 | Panel | Interactive | LM.Cannabis_x_Herb.THC_x_Daily.Interpol. | Herb | 0.0008 | 14.37 | 4.18 |
| Trisomy 13 | Spatial | Interactive | LM.Cannabis_x_Herb.THC_x_Daily.Interpol. | Herb | 0.0008 | 14.37 | 4.18 |
| Chromosomes | Panel | 1 Lag | LM.Cannabis_x_Herb.THC_x_Daily.Interpol. | Herb | 0.0002 | 8.08 | 3.58 |

| | | | | | | | |
|---|---|---|---|---|---|---|---|
| Chromosomes | Spatial | 1 Lag | LM.Cannabis_x_Herb.THC_x_Daily.Interpol. | Herb | 0.0002 | 8.08 | 3.58 |
| Genetic | Panel | 1 Lag | Tobacco: LM.Cannabis_x_Herb.THC | Herb | 0.0023 | 3.40 | 1.91 |
| Genetic | Spatial | 1 Lag | Tobacco: LM.Cannabis_x_Herb.THC | Herb | 0.0023 | 3.40 | 1.91 |
| Chromosomes | Spatial | 1 Lag | LM.Cannabis_x_Resin.THC | Resin | <2.2E-16 | 1.84E+03 | 510.05 |
| Trisomy 18 | Panel | Additive | LM.Cannabis_x_Resin.THC | Resin | 4.94E-11 | 927.29 | 176.39 |
| Trisomy 18 | Spatial | Additive | LM.Cannabis_x_Resin.THC | Resin | 4.94E-11 | 927.29 | 176.39 |
| Genetic | Panel | Interactive | LM.Cannabis_x_Herb.THC | Resin | 0.0076 | 4.13E+05 | 61.12 |
| Genetic | Spatial | Interactive | LM.Cannabis_x_Herb.THC | Resin | 0.0076 | 4.13E+05 | 61.12 |
| Trisomy 18 | Panel | 2 Lags | LM.Cannabis_x_Resin.THC | Resin | <2.2E-16 | 101.56 | 59.31 |
| Trisomy 18 | Spatial | 2 Lags | LM.Cannabis_x_Resin.THC | Resin | <2.2E-16 | 101.56 | 59.31 |
| Chromosomes | Panel | Additive | LM.Cannabis_x_Resin.THC | Resin | 1.36E-07 | 18.49 | 8.15 |
| Chromosomes | Spatial | Additive | LM.Cannabis_x_Resin.THC | Resin | 1.36E-07 | 18.49 | 8.15 |
| Chromosomes | Panel | Interactive | LM.Cannabis_x_Resin.THC | Resin | 0.0077 | 74.39 | 4.94 |
| Chromosomes | Spatial | Interactive | LM.Cannabis_x_Resin.THC | Resin | 0.0077 | 74.39 | 4.94 |
| Turners | Panel | Additive | LM.Cannabis_x_Resin.THC | Resin | 0.0055 | 3.14 | 1.79 |
| Turners | Spatial | Additive | LM.Cannabis_x_Resin.THC | Resin | 0.0055 | 3.14 | 1.79 |

**Table S25**. Summary of E-Values by Group of Covariates

| Group | Number | Mean Minimum E-Value | Median Minimum E-Value | Minimum Minimum E-Value | Maximum Minimum E-Value | Mean E-Value Estimate | Median E-Value Estimate | Minimum E-Value Estimate | Maximum E-Value Estimate |
|---|---|---|---|---|---|---|---|---|---|
| | | | | | | | | | |
| Daily | 30 | 1.47E+306 | 1.91E+25 | 3.51 | 1.10E+307 | 1.47E+306 | 1.81E+31 | 2.78E+08 | 1.10E+307 |
| Herb | 34 | 1.31E+19 | 58.55 | 1.90 | 2.23E+20 | 1.66E+44 | 2.24E+05 | 2.18 | 2.82E+45 |
| Resin | 22 | 213.63 | 21.84 | 1.79 | 1490.00 | 4.86E+04 | 101.56 | 3.14 | 4.13E+05 |

**Table S26**. Wilcoxson Tests for Intergroup Comparison of Grouped Covariates

| Comparison | W-Statistic | Alternative | *p*-Value |
|---|---|---|---|
| | | | |
| Lower E-Value, Daily_v_Herb | 788 | two.sided | 1.87E-04 |
| Lower E-Value, Daily_v_Resin | 588 | two.sided | 1.81E-06 |
| Lower E-Value, Herb_v_Resin | 464 | two.sided | 0.1330 |
| E-Value Estimate, Daily_v_Herb | 864 | two.sided | 1.95E-06 |
| E-Value Estimate, Daily_v_Resin | 660 | two.sided | 1.01E-09 |
| E-Value Estimate, Herb_v_Resin | 492 | two.sided | 0.0486 |

**Table S27**.Epigenomic Hits for PPP1CC from Schrott Data 2021

| Nearest Gene Name | Page | Functional Annotation | Number Genes Identified | *p*-Value | Bonferroni Adjusted *p*-Value |
|---|---|---|---|---|---|
| | | | | | |
| PPP1CC | 236 | Focal Adhesion | 39 | 0.000934 | 0.226633 |
| PPP1CC | 236 | Dopaminergic synapse | 27 | 0.001420 | 0.32431 |
| PPP1CC | 236 | Long term potentiation | 17 | 0.017678 | 0.385287 |
| PPP1CC | 236 | Vascular smooth muscle contraction | 25 | 0.001862 | 0.400987 |
| PPP1CC | 236 | Inflammation, TRP channel regulator | 22 | 0.001999 | 0.423304 |
| PPP1CC | 236 | cGMP-PKG signalling | 30 | 0.003895 | 0.658065 |
| PPP1CC | 236 | Actin cytoskeleton regulation | 37 | 0.004587 | 0.717564 |
| PPP1CC | 237 | Hippo signalling | 16 | 0.040247 | 1.000000 |
| PPP1CC | 237 | Oxytocin signalling | 31 | 0.044117 | 1.000000 |
| PPP1CC | 237 | cAMP signalling | 20 | 0.046734 | 1.000000 |
| PPP1CC | 237 | Insulin signalling | 54 | 0.067770 | 1.000000 |
| PPP1CC | 238 | Platelet Activation | 21 | 0.079093 | 1.000000 |
| PPP1CC | 238 | Cardiomyocyte adrenergic signalling | 22 | 0.079093 | 1.000000 |

# Supplementary Figure

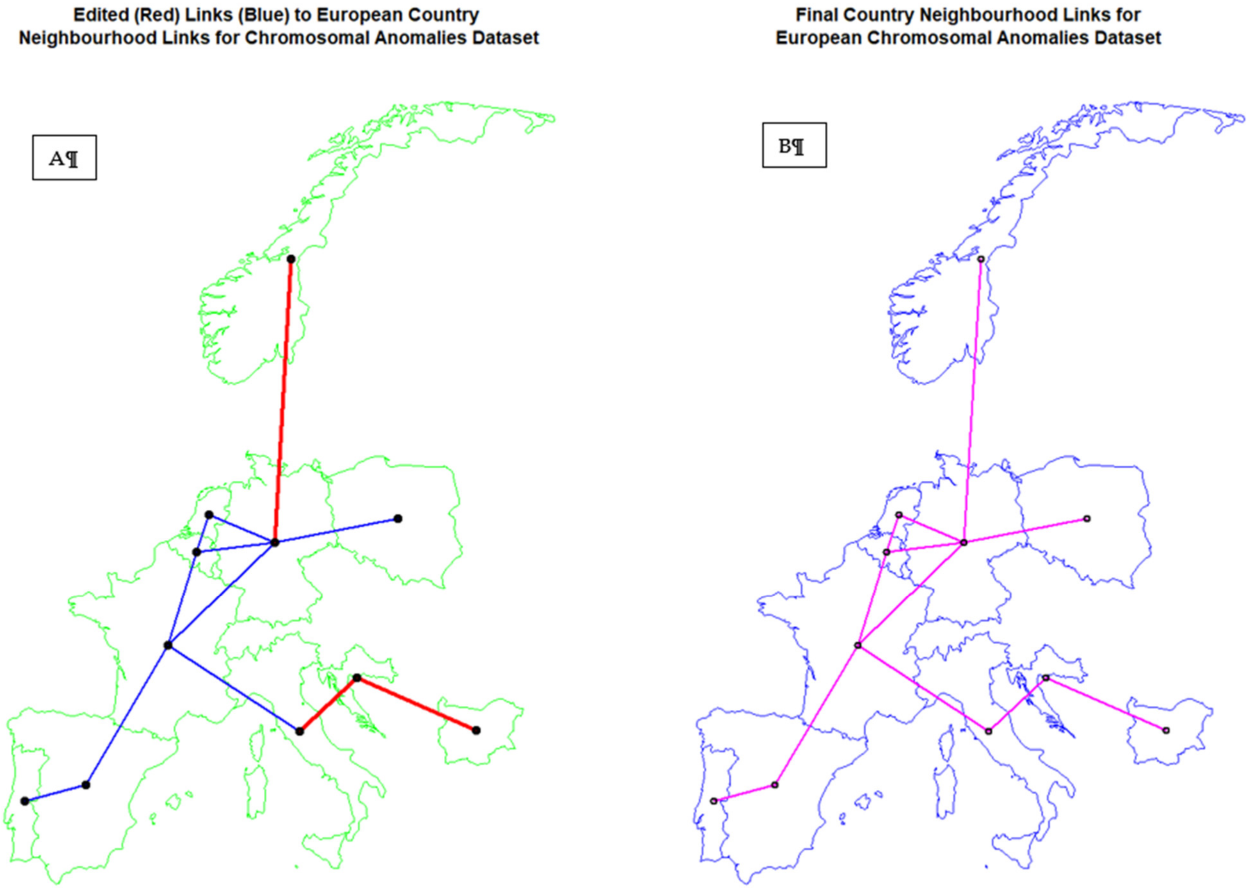


**Figure S1**. International spatial links for European Chromosomal dataset (**A**) edited and (**B**) final.
